# Prognostic impact of guideline-directed medical therapy after functionally complete revascularisation in patients with obstructive coronary artery diseases

**DOI:** 10.1136/heartjnl-2025-325670

**Published:** 2025-08-07

**Authors:** Yingyang Geng, Changdong Guan, Yao Jiang, WeiXian Yang, Bo Yu, Guosheng Fu, Jun Pu, Xinkai Qu, Qi Zhang, Yanyan Zhao, Lilei Yu, Yunfei Huang, Shengxian Tu, Shubin Qiao, Lei Song

**Affiliations:** 1 Department of Cardiology, National Clinical Research Center for Cardiovascular Diseases, Fuwai Hospital, National Center for Cardiovascular Diseases, Chinese Academy of Medical Sciences and Peking Union Medical College, Beijing, China; 2 Department of Cardiology, The Second Affiliated Hospital of Harbin Medical University, Harbin, Heilongjiang, China; 3 Department of Cardiology, Sir Run Run Shaw Hospital, Zhejiang University School of Medicine, Hangzhou, Zhejiang Province, China; 4 Department of Cardiology, Renji Hospital, Shanghai Jiao Tong University School of Medicine, Shanghai, China; 5 Department of Cardiology, Huadong Hospital Affiliated to Fudan University, Shanghai, China; 6 Department of Cardiology, Shanghai East Hospital, Tongji University School of Medicine, Shanghai, China; 7 Medical Research and Biometrics Center, National Clinical Research Center for Cardiovascular Diseases, Fuwai Hospital, National Center for Cardiovascular Diseases, Chinese Academy of Medical Sciences and Peking Union Medical College, Beijing, China; 8 Department of Cardiology, Renmin Hospital of Wuhan University; Cardiac Autonomic Nervous System Research Center of Wuhan University; Cardiovascular Research Institute, Wuhan University; Hubei Key Laboratory of Cardiology, Wuhan, Hubei Province, China; 9 Biomedical Instrument Institute, School of Biomedical Engineering, Shanghai Jiao Tong University, Shanghai, China

**Keywords:** Percutaneous Coronary Intervention, Pharmacology, Clinical, Treatment Outcome, Coronary Artery Disease

## Abstract

**Objective:**

Functional complete revascularisation (FCR) has been proven to be associated with superior prognosis following percutaneous coronary intervention. Whether guideline-directed medical therapy (GDMT) still impacts clinical outcomes in patients who have achieved FCR requires further evaluation.

**Methods:**

The study population was drawn from patients who achieved FCR in the FAVOR III China trial, defined as a quantitative flow ratio (QFR)-based residual functional Synergy between percutaneous coronary intervention with taxus and cardiac Surgery score of 0, measured only in vessels with QFR≤0.80. GDMT was defined as the combination of single or dual antiplatelet therapy, a beta-blocker and a statin, with or without an ACE inhibitor or angiotensin receptor blocker, according to contemporary guideline recommendations. Patients were categorised into the GDMT group (compliance with all 4 agents) or non-GDMT group (compliance with 0–3 agents). The primary endpoint was major adverse cardiac and cerebrovascular events (MACCE) at 3 years, a composite of death, myocardial infarction, stroke and ischaemia-driven revascularisation.

**Results:**

Among 3221 (85.2%) patients who achieved FCR, a total of 1964 (61.2%), 1919 (59.9%), 1545 (48.4%), 1483 (46.6%) and 1084 (35.3%) patients adhered to GDMT at 1 month, 6 months, 1 year, 2 years and 3 years, respectively. The MACCE occurred in 313 (10.2%) patients through 3 years. The rate of MACCE was similar between GDMT and non-GDMT groups within the first year, but significantly lower in the GDMT group from the second year (adjusted HR: 0.66, 95% CI: 0.51 to 0.85; p<0.01) and sustained until the third year (adjusted HR: 0.65, 95% CI: 0.50 to 0.85; p<0.01), compared with the non-GDMT group.

**Conclusions:**

In patients who achieved FCR, the benefit of good adherence to GDMT remained significant, starting from the second year and continuing up to 3 years.

**Trial registration number:**

NCT03656848.

**WHAT IS ALREADY KNOWN ON THIS TOPIC**

- Despite advances in percutaneous coronary intervention (PCI) techniques and devices for the treatment of obstructive coronary artery disease, guideline-directed medical therapy (GDMT) is still needed.
- Functional complete revascularisation (FCR) has been proven to be associated with superior prognosis following PCI.

**WHAT THIS STUDY ADDS**

- In patients who achieved FCR, consistent adherence to GDMT was associated with significant cardiovascular benefits beginning in the second year and persisting through the third year.

**HOW THIS STUDY MIGHT AFFECT RESEARCH, PRACTICE OR POLICY**

- Maintaining adherence to GDMT continues to confer cardiovascular advantages in patients achieving FCR.

## Introduction

 Coronary artery disease (CAD) remains the leading cause of mortality worldwide and contributes significantly to the global burden of cardiovascular diseases.^1^ Percutaneous coronary intervention (PCI) has become a widely adopted approach for managing CAD over recent decades.^2^,^4^ Despite considerable advancements in PCI techniques and devices, there remains a need for guideline-directed medical therapy (GDMT). Notably, the ISCHEMIA (International Study of Comparative Health Effectiveness with Medical and Invasive Approaches) trial demonstrated that among patients with stable coronary disease and moderate or severe ischaemia, optimal medical treatment achieved comparable outcomes with initial invasive strategy of revascularisation.^5^ Additionally, extensive evidence confirms that GDMT is the only treatment that actually demonstrated improved outcomes on hard clinical endpoints among patients with stable ischaemic heart disease.^6^,^8^

Although guidelines strongly recommend,^9 10^ adherence to GDMT is lower than expected in real-world practice due to many barriers. Recent reports indicate that only 50%–65% of patients receive optimal pharmacological secondary prevention following PCI,^11^,^13^ highlighting a gap between current guidelines recommendations and clinical practice. Meanwhile, recent research demonstrates that patients undergoing PCI with fractional flow reserve (FFR) or quantitative flow ratio (QFR) guided functional complete revascularisation (FCR) had better prognosis than those without FCR non-complete revascularisation.^14 15^ Furthermore, an increasing number of clinical trials support shorter durations of dual antiplatelet therapy (DAPT).^16 17^ These observations highlight an important clinical question: whether the proven benefits of GDMT extend equally to patients who achieve satisfactory results following FCR.

The FAVOR III China (Comparison of Quantitative Flow Ratio Guided and Angiography Guided Percutaneous Intervention in Patients with Coronary Artery Disease) is a randomised trial comparing the clinical prognosis of PCI guided by QFR, a novel computational approach for assessing FFR from standard angiograms, with conventional angiographic guidance among patients with CAD.^18^,^20^ In this post hoc analysis, we sought to evaluate the impact of GDMT adherence on long-term prognosis, in a special population achieving FCR.

## Method

### Population

FAVOR III China was a multicentre, investigator-initiated, randomised trial conducted between December 2018 and January 2020, involving 26 hospitals across China. In this study, 3825 patients were enrolled and randomly assigned to either QFR-guided lesion selection strategy or angiography-guided strategy. Details of the study design, inclusion/exclusion criteria and outcomes have been previously reported.^18^,^20^ In brief, the eligibility criteria included adult patients with stable or unstable angina, or post-myocardial infarction (MI) for at least 72 hours before screening, presenting with visually estimated angiographic evidence of at least one lesion with a percentage diameter stenosis of 50%–90% in a coronary artery with a reference vessel diameter of at least 2.5 mm. Patients in the QFR-guided group received treatment (either PCI or deferral) based on online QFR evaluation, while patients in the angiography-guided group were treated based on visual angiographic assessment in accordance with the standard practice at each participating centre. Optimal medical therapy was required in both groups during follow-up based on physician decision and local standard practice.^19^

Follow-up telephone or clinic visits were scheduled at 1 month, 6 months, 1 year and annually up to 5 years after the index procedure, with a comprehensive medication history obtained at each scheduled time point. The study was registered on ClinicalTrials.gov (NCT03656848).

### QFR assessment

Angiographic images from all FAVOR III China trial participants were collected and transmitted to a blinded independent angiographic core laboratory (Core Medical, Beijing, China) for offline QFR and quantitative coronary angiography analysis. Details of offline QFR measurement have been previously described.^15 21^ In brief, QFR was calculated using dedicated software (AngioPlus system, Pulse Medical, Shanghai, China) based on two ≥25° angiograms from pre-PCI and post-PCI, respectively. The lumen contour and reference vessel diameter were automatically delineated, with manual correction for incorrect vessel identification following a standard operating procedure. The analyst selected the angiographic sequence, the starting and the final frame (contrast entered and reached distal vessel). QFR accounted for the entire contoured segment from ostium to distal vessel. Contrast flow velocity was calculated using the Thrombolysis in Myocardial Infarction frame count. With these parameters, the QFR value was calculated. For bifurcation lesions, QFR assessed both the main vessel and side branch. Details of the QFR measurement process are described in the online supplemental Appendix.

### Functional complete revascularisation

The anatomic Synergy between percutaneous coronary intervention with taxus and cardiac Surgery (SYNTAX) score (SS) quantifies CAD complexity. Its calculation is based on lesion location, involved segments and lesion complexity as observed in coronary angiograms.^22^ The QFR-based functional SS (FSS) was calculated by summing the segmental anatomic SYNTAX scores only for vessels exhibiting physiological ischaemia, defined by an offline vessel QFR≤0.80. FCR was defined as a residual FSS (rFSS)=0 by the offline QFR analysis based on final angiograms. Participants in the FAVOR III China trial who achieved FCR were included in this post hoc analysis.

### Guideline-directed medical therapy

For the present analysis, patient adherence to GDMT following the procedure was primarily determined according to the guidelines of the European Society of Cardiology, American Heart Association and Chinese cardiology guidelines.^7 8 23 24^ According to the study protocol, we evaluated the utilisation of the four guideline-directed medications at each time point during 3-year follow-up, including antiplatelet therapy, ACE inhibitor (ACEI) or angiotensin receptor blocker (ARB), beta-blocker and statins. Patients’ medication adherence was determined based on actual medication usage collected during follow-up via clinical visits or telephone. Details of the GDMT recommendations in different clinical scenarios are listed in online supplemental eTable 1. In brief, the GDMT included: (1) DAPT with aspirin and a P2Y12 inhibitor (clopidogrel or ticagrelor) for at least 12 months in patients with ST-segment elevation MI (STEMI) or non-STEMI (NSTEMI), and for at least 6 months among chronic coronary syndrome (CCS) patients after stent implantation; followed by single antiplatelet therapy with aspirin or clopidogrel for long-term treatment. (2) Beta-blockers were used for patients with systolic left ventricular dysfunction or heart failure with reduced left ventricular ejection fraction (LVEF), as well as those with MI history; (3) ACEI or ARBs were recommended for patients with heart failure and reduced LVEF (<40%), diabetes or chronic kidney disease, unless contraindicated; (4) Statin therapy was recommended for all patients, unless contraindicated.

Patients were categorised into the GDMT group (adhere to all 4 agents) or the non-GDMT group (adhere to 0–3 agents). For each GDMT agent, medication adherence was tracked throughout the 3-year follow-up. If patients discontinued medication for over 1 month as recommended by guidelines at each follow-up visit, they were considered not to adhere to GDMT and classified as non-GDMT for this and subsequent follow-up periods.

### Outcomes

The primary endpoint of the present study was major adverse cardiac and cerebrovascular events (MACCE) at 3 years, defined as a composite of death, MI, stroke and ischaemia-driven revascularisation. Secondary endpoints included each individual component of MACCE, periprocedural and spontaneous MI, and MACCE excluding periprocedural MI (PMI). The definition of PMI was the same as the original study, creatine kinase myocardial band (CK-MB) rise to >3 times the upper limit of normal (ULN; or above the most recent level for acute coronary syndromes (ACS) patients), or a troponin elevation of >21 times ULN if baseline CK-MB was not available. Specific definitions of other clinical endpoints have been previously reported.^18^,^20^ All clinical outcomes were adjudicated by an independent clinical events committee that was masked to randomisation.

### Statistical analysis

Baseline characteristics of participants were described by GDMT and non-GDMT groups at the 3-year follow-up. Categorical variables are presented as numbers and percentages and were compared using the likelihood ratio χ² test or Fisher’s exact test, while continuous variables are presented as mean±SD or median (IQR), depending on distribution, and were compared using the two-sample t-test or Mann-Whitney U test.

The event rates of MACCE during the 3-year follow-up period in the GDMT and non-GDMT groups were estimated using Kaplan-Meier methods and compared using log-rank tests. Cox proportional hazards models or time-dependent Cox models were applied based on the results of Schoenfeld residual tests assessing the proportional hazards assumption. To address potential confounding, a stratified Cox regression model was constructed in the propensity score-matched population, with GDMT included as a time-dependent covariate. Propensity scores were estimated using a multivariable logistic regression model, incorporating the following variables: age, gender, diabetes mellitus, hypertension, hypercholesterolaemia, current smoking status, family history of CAD, previous MI, ACS and PCI treatment (online supplemental eTable 2). These covariates were selected based on standardised differences greater than 0.15—indicating imbalance^25^—and their clinical relevance as supported by prior literature.^1526^,^31^ Additionally, a multivariable Cox regression model adjusting for these variables was performed to further assess the association between GDMT use and MACCE outcomes. Between-group risks were reported as HRs with corresponding 95% CIs. We further performed a sensitivity analysis to evaluate the risks for the primary endpoint at each time point between different levels of GDMT adherence (adhere to 3–4 agents vs adhere to 0–2 agents, and adhere to 2–4 agents vs adhere to 0–1 agents), the impact of each prescribed medication on MACCE at each follow-up time point, the effect of QFR-guided versus angiography-guided treatment, and outcomes within the subgroup of patients who underwent PCI. At each follow-up timepoint, we only analysed patients with complete follow-up information and medication information. Those who withdrew from the trial, were lost to follow-up, or lacked medication information were excluded from the final assessment at each timepoint. A p<0.05 was considered statistically significant. All analyses were performed using SAS software, V.9.4 (SAS Institute).

## Results

### Baseline characteristics

**Figure 1:**
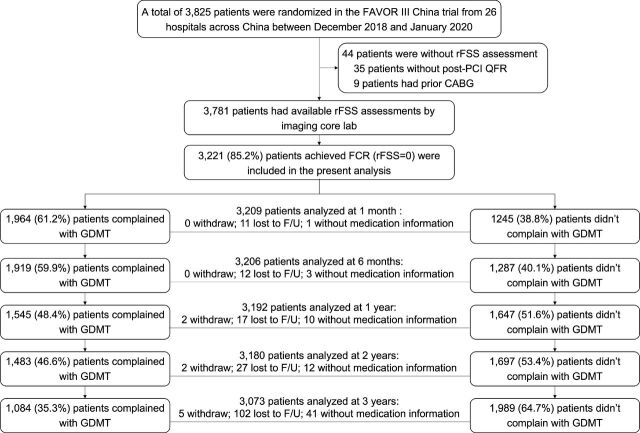
Patient flow chart. A total of 3209 (99.6%), 3206 (99.5%), 3192 (99.1%), 3180 (98.7%) and 3073 (95.4%) patients, who completed clinical follow-ups (FU) and had complete medication information, were included in the analyses at 1 month, 6 months, 1 year, 2 years and 3 years, respectively. CABG, coronary artery bypass grafting; FCR, functionally complete revascularisation; GDMT, guideline-directed medical therapy; QFR, quantitative flow ratio; rFSS, residual functional SYNTAX (Synergy between percutaneous coronary intervention with taxus and cardiac Surgery) score.

Among the 3781 patients with evaluable rFSS, 3221 (85.2%) achieved QFR-based FCR. Of these, 3209 (99.6%), 3206 (99.5%), 3192 (99.1%), 3180 (98.7%) and 3073 (95.4%) patients, who completed clinical follow-ups with complete medication data at 1 month, 6 months, 1 year, 2 years and 3 years, respectively, were included in the analyses. At 3 years, 1084 (35.3%) patients were categorised into the GDMT group, while the remaining 1989 (64.7%) patients, who withdrew from at least one agent, were categorised into the non-GDMT group (figure 1).

Of the 3221 FCR patients, the mean age was 62.7±10.1 years, 70% were male, 33% had diabetes mellitus, 65.1% had hypertension, 36.9% had hypercholesterolaemia, 8.6% had a prior MI and 63.7% presented with ACS, as shown in table 1. Younger patients, smokers, those with a family history of CAD, ACS and those undergoing PCI were more likely to adhere to GDMT for 3 years. In contrast, patients with concomitant diseases such as diabetes, hypertension and those with prior MI, PCI or stroke history tended to discontinue at least one agent over the 3 years.

**Table 1:** Baseline characteristics according to GDMT status at 3 years

	GDMT (n=1084）	Non-GDMT (n=1989）	P value
Age, years	61.5±10.3	63.2±9.9	<0.01
Female gender	325 (30.0)	603 (30.3)	0.85
Diabetes mellitus	146 (13.5)	870 (43.7)	<0.01
Hypertension	646 (59.6)	1357 (68.2)	<0.01
Hypercholesterolaemia	481 (44.4)	687 (34.5)	<0.01
Current smoker	366 (33.8)	562 (28.3)	<0.01
Family history of CAD	104 (9.6)	128 (6.4)	<0.01
Previous MI	59 (5.4)	207 (10.4)	<0.01
Previous PCI	216 (19.9)	507 (25.5)	<0.01
Previous stroke	82 (7.6)	195 (9.8)	0.04
Peripheral artery disease	32 (3.0)	61 (3.1)	0.86
Stable angina	242 (22.3)	537 (27.0)	<0.01
Acute coronary syndromes	760 (70.1)	1198 (60.2)	<0.01
Estimated glomerular filtration rate, mL/min/1.73 m^2^	69.8 (57.8, 57.4)	69.9 (83.1, 83.2)	0.91
Left ventricular ejection fraction, %	64±6.0	63±6.6	0.75
Number of diseased vessels			0.26
One-vessel disease	49.2 (533)	1040 (52.3)	
Two-vessel disease	383 (35.3)	646 (32.5)	
Three-vessel disease	150 (13.8)	261 (13.1)	
Left main disease	18 (1.7)	42 (2.1)	
PCI performed	1064 (98.2)	1837 (92.4)	<0.01

Values are n (%); Continuous data are expressed as mean±SD or median (IQR).

CAD, coronary artery disease; GDMT, guideline-directed medical therapy; MI, myocardial infarction; PCI, percutaneous coronary intervention.

### Guideline-directed medical therapy

A total of 1964 (61.2%), 1919 (59.9%), 1545 (48.4%), 1483 (46.6%) and 1084 (35.3%) patients adhered to four GDMT agents at 1 month, 6 months, 1 year, 2 years, and 3 years, respectively (online supplemental eFigure 1). As illustrated in online supplemental eFigure 3, over a period of 3 years, approximately 30%–40% of patients consistently adhered to 3 prescribed agents. Meanwhile, the proportion of patients adhering to only 0–2 agents showed a gradual increase, reaching up to 25% at the end of the period. The utilisation of each prescribed agent over 3 years is listed in online supplemental eFigure 4. The proportions of patients compliant with antiplatelet drugs, beta-blockers, ACEI or ARB, and statins at 3 years were 71.3%, 95.0%, 63.5% and 77.0%, respectively.

### Clinical outcomes

**Figure 2:**
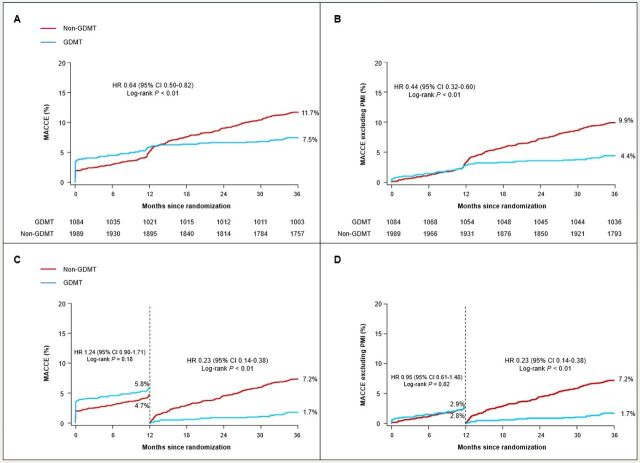
Kaplan-Meier curves for MACCE and MACCE excluding PMI over 3 years (**A**) Kaplan-Meier curves for MACCE; (**B**) Kaplan-Meier curves for MACCE excluding PMI; (**C**) land-mark analyses for MACCE; (**D**) land-mark analyses for MACCE excluding PMI. Adjusted HRs with 95% CIs are shown. MACCE is defined as a composite of death, all myocardial infarction, ischaemia-driven revascularisation or stroke. GMDT, guideline-directed medical therapy; MACCE, major adverse cardiac and cerebrovascular events; PMI, periprocedural myocardial infarction.

The MACCE occurred in 313 (10.2%) patients within 3 years, including 81 (7.5 %) patients in the GDMT group and 232 (11.7%) in the non-GDMT group (HR: 0.64; 95% CI: 0.50 to 0.82; p<0.01). Similarly, the 3-year MACCE rate excluding PMI was significantly lower in the GDMT group (4.4% vs 9.9%; HR: 0.44; 95% CI: 0.32 to 0.60; p<0.01) (figure 2, table 2). Both outcomes showed marked divergence primarily after the first year (HR: 0.23; 95% CI: 0.14 to 0.38; p<0.01), reflecting the identical event rates beyond the first year (figure 2). This reduction was driven mainly by fewer ischaemia-driven revascularisations (HR: 0.22; 95% CI: 0.13 to 0.35; p<0.01) (table 2). Additionally, the multivariable Cox regression analysis showed a benefit of GDMT on MACCE after 2 years (adjusted HR: 0.66, 95% CI: 0.51 to 0.85; p<0.01) and sustained at 3 years (adjusted HR: 0.65, 95% CI: 0.50 to 0.85; p<0.01) (online supplemental eFigure 2).

**Table 2:** A 3-year clinical outcomes

	Total population (n=3073)	GDMT (n=1084）	Non-GDMT (n=1989）	Unadjusted HR (95% CI)	P value
MACCE	313 (10.2)	81 (7.5)	232 (11.7)	0.64 (0.50 to 0.82)	<0.01
MACCE excluding PMI	244 (7.9)	48 (4.4)	196 (9.9)	0.44 (0.32 to 0.60)	<0.01
All-cause death	11 (0.4)	4 (0.4)	7 (0.4)	1.05 (0.31 to 3.58)	0.94
Myocardial infarction	146 (4.8)	59 (5.4)	87 (4.4)	1.26 (0.90 to 1.75)	0.18
Periprocedural MI	78 (2.5)	39 (3.6)	39 (2.0)	1.84 (1.18 to 2.87)	<0.01
Spontaneous MI	70 (2.3)	22 (2.0)	48 (2.4)	0.84 (0.51 to 1.40)	0.51
Ischaemia-driven revascularisation	167 (5.4)	18 (1.7)	149 (7.5)	0.22 (0.13 to 0.35)	<0.01
Stroke	21 (0.7)	5 (0.5)	16 (0.8)	0.57 (0.21 to 1.56)	0.28

Values are n (%). Percentages are Kaplan-Meier estimates.

GDMT, guideline-directed medical therapy; MACCE, major adverse cardiac and cerebrovascular events; MI, myocardial infarction; PMI, periprocedural myocardial infarction.

After propensity score matching, GDMT remained independently associated with significantly reduced in MACCE through 3 years (adjusted HR: 0.52, 95% CI: 0.36 to 0.75; p<0.01), as well as MACCE excluding PMI (adjusted HR: 0.38, 95% CI: 0.25 to 0.58; p<0.01), driven mainly by a reduced risk of ischaemia-driven revascularisation (adjusted HR: 0.14, 95% CI: 0.07 to 0.28; p<0.01) (table 3).

**Table 3:** Cox regression models for adverse events with GDMT as a time-dependent covariate

	Adjusted HR (95% CI)	P value
MACCE	0.52 (0.36 to 0.75)	<0.01
MACCE excluding PMI	0.38 (0.25 to 0.58)	<0.01
All-cause death	1.00 (0.25 to 4.00)	>0.99
Myocardial infarction	1.14 (0.69 to 1.90)	0.61
Spontaneous MI	0.80 (0.41 to 1.54)	0.51
Ischaemia-driven revascularisation	0.14 (0.07 to 0.28)	<0.01
Stroke	0.25 (0.03 to 2.24)	0.22

The propensity score model included the following variables: age, gender, diabetes mellitus, hypertension, hypercholesterolaemia, current smoking status, family history of coronary artery disease, previous myocardial infarction, acute coronary syndromes and PCI treatment.

GDMT, guideline-directed medical therapy; MACCE, major adverse cardiac and cerebrovascular events; MI, myocardial infarction; PCI, percutaneous coronary intervention; PMI, periprocedural myocardial infarction.

### Subgroup and sensitivity analyses

The results of the subgroup analysis show that in both the ACS and CCS, GDMT can reduce the incidence of MACCE. The main benefit is derived from ischaemia-driven revascularisation, which is consistent with the main findings of the study. No interaction was observed in terms of both the primary and secondary endpoints (online supplemental eTable 3). In sensitivity analyses, the benefit of reduced risk of MACCE was significant when patients adhered to at least three prescribed drugs for up to 2 years (adjusted HR: 0.62, 95% CI: 0.45 to 0.85; p<0.01) and was sustained for 3 years (adjusted HR: 0.63, 95% CI: 0.49 to 0.81; p<0.01). However, when patients adhered to only two prescribed drugs or fewer, there was no benefit in terms of MACCE events through 3 years (online supplemental eFigure 5). The benefit of GDMT on the risk reduction of MACCE events and MACCE events excluding PMI at 3 years did not differ between the angiography-guided and QFR-guided strategy groups (online supplemental eTable 4). Additionally, among the patients who received PCI, those who adhered to GDMT had a significant benefit compared with those who did not, which was consistent with the overall population (online supplemental eTable 5). Notably, among the four medications analysed, adherence to antiplatelet therapy demonstrated the strongest protective effect, becoming significant starting from 2 years (adjusted HR: 0.46, 95% CI: 0.36 to 0.60; p<0.01) and persisted through 3 years (adjusted HR: 0.37, 95% CI: 0.29 to 0.46; p<0.01) (online supplemental eFigure 6).

## Discussion

The present study is a post hoc analysis of the randomised FAVOR III China trial focusing on the long-term impact of GDMT adherence among patients who achieved QFR-based functionally complete revascularisation. The main findings of the current study were: (1) the adherence rate of GDMT started from 61.2% at 1 month and decreased to 35.3% by 3 years, indicating a relatively lower level than expected over time in this cohort; (2) even after optimal FCR, adherence to GDMT remained associated with a significant reduction of MACCE, particularly for ischaemia-driven revascularisation; (3) the benefit was also significant when patients adhered to at least three prescribed drugs and (4) among the four medications analysed, adherence to antiplatelet therapy demonstrated the strongest protective effect.

The major objective of the FAVOR III China trial is to investigate whether a QFR-guided PCI strategy is superior to a traditional angiography-guided strategy. Consistent with previous studies using pressure wire-based physiological assessment,^1432^,^36^ a strategy of QFR-based lesion selection is superior to angiography guidance.^19 20^ Although there was no functional guidance in the control group, over 80% of patients achieved functionally complete revascularisation. In terms of secondary prevention by postprocedure medical advice, there were no differences between the groups. In the present study, we combined patients who achieved functionally complete revascularisation from both groups. Limited by the data collected, our study focused on four common medications used by CAD patients following PCI. Our results indicate that adherence to the guidelines, particularly for more than three agents, can significantly improve prognosis in patients who already achieved functionally complete revascularisation. Additionally, the beneficial effects of GDMT are not immediate but accumulate over time, becoming statistically significant starting from approximately 2 years of optimal adherence. This delayed benefit may partially reflect similar medication adherence rates between groups early in follow-up, with differences becoming more pronounced as adherence levels decline over time.

Consistent with previous studies,^37^,^39^ despite strong evidence supporting cardiovascular drug therapies for risk reduction in CAD, a significant gap persists in China between guideline recommendations and secondary prevention. This gap is exacerbated by the high prevalence and poor management of major risk factors, such as hypertension, dyslipidaemia and diabetes mellitus, especially in elderly, rural populations.^8^ In our study, only 61.2% of patients persist with GDMT at 1 month, and eventually, fewer than one-third of individuals with CVDs were routinely treated with any proven secondary preventive drugs at 3 years of follow-up. Among the four medications (antiplatelet drugs, beta-blockers, ACEI/ARBs and statins), beta-blockers had the highest adherence, with over 95% adherence at 3 years. Adherence to the other three medications was relatively low, especially ACEI (or ARBs), with less than 65% of patients maintaining adherence over 3 years, notably among those with NSTEMI combined with DM and LVEF <40% (76.5%), or CCS patients with hypertension not taking ACEI/ARBs (75.3%). Less than 71.3% of antiplatelet medications were administered in accordance with GDMT and over 25% of patients with CCS were not receiving statins at 3 years, which is far more than expected. These findings align with a recent a recent post hoc analysis of the J-CONFIRM registry, which demonstrated that adherence to GDMT over a 2-year period significantly reduced the 5-year MACE in patients who deferred PCI after FFR assessment in a CCS population. Furthermore, the analysis revealed that only 12.5% of CCS patients adhered to GDMT for 2 years.^39^ Possible explanations included lack of medication adherence awareness, adverse drug reactions and weaknesses in long-term chronic disease management in the current medical care system. Furthermore, our findings demonstrated that antiplatelet therapy had the strongest association with improved clinical outcomes, re-emphasising the importance of antiplatelet medications, particularly given recent trends towards shorter DAPT durations.^40^ However, these results may be limited by sample size and inconsistent adherence rates, particularly with beta-blockers. Further prospective studies are warranted to provide more robust evidence.

Atherosclerosis is a complex process involving multiple mechanisms.^41 42^ Despite the high immediate success rate of contemporary PCI procedures, the long-term revascularisation rate remains significant, ranging from about 3%–10% within the first year.^43^,^45^ While studies focusing on PCI techniques, intravascular imaging or physiological guidance aim to achieve better acute results, we should not neglect the importance of GDMT as optimal secondary prevention. The present results demonstrate that standardised GDMT can improve prognosis even among patients who achieve functionally complete revascularisation.

### Limitations

The trial has several limitations. First, the study population was derived from the FAVOR III China study, which excluded patients with moderate to severe chronic kidney disease, acute and certain complex lesions. As a result, the enrolled patients might represent a lower-risk population compared with those typically encountered in routine clinical practice. Second, we did not collect socioeconomic or psychological factors, which may introduce unmeasured confounding and affect the observed associations. As prior studies^46^,^48^ have shown that low income, limited access to healthcare in underdeveloped regions, low medical insurance reimbursement, depressive symptoms and reduced health-related quality of life (eg, lower EuroQol 5-Dimension Questionnaire scores) can negatively impact medication adherence and increase the risk of MACCE. The absence of these data might limit our ability to evaluate the influence of these factors and may contribute to selection bias. Third, the study classified patients who did not regularly take guideline-recommended drugs at any follow-up time point as the non-GDMT group. These patients might resume GDMT for various reasons, potentially affecting the research results. Fourth, a significant proportion of the study population (51.2%) had single-vessel disease, which may attenuate the benefit of FCR typically seen in multivessel disease. Fifth, although multivariate regression modelling was used to adjust for potential confounders, the possibility of residual confounding cannot be completely ruled out. Sixth, the 3-year follow-up duration may not be sufficient to capture long-term clinical outcomes and the sustained impact of GDMT adherence over time. Finally, missing data were handled using a complete-case analysis approach, which may introduce bias if the missingness is not completely at random. This limitation might affect the internal validity and generalisability of our findings, as patients with incomplete data may systematically differ in important ways from those with complete follow-up and medication information.

## Conclusions

In patients with obstructive CAD who have undergone FCR, continued secondary prevention with guideline-recommended drug treatment was associated with a beneficial prognosis. The reduced risk of MACCE began at 2 years and increased up to 3 years.

## Supplementary material

10.1136/heartjnl-2025-325670 online supplemental file 1

10.1136/heartjnl-2025-325670 online supplemental file 2

## Data Availability

All data relevant to the study are included in the article or uploaded as supplementary information.
