# Supplementary material for: Prognostic impact of guideline-directed medical therapy after functionally complete revascularisation in patients with obstructive coronary artery diseases
Source: Heart. 2025 Aug 7;112(5):e325670. doi: 10.1136/heartjnl-2025-325670 (PMC13271866; doi:10.1136/heartjnl-2025-325670)
Supplement: online supplemental file 1 [file heartjnl-112-5-s001.pdf]

Protocol

This trial protocol has been provided by the authors to give readers additional information about their work.

Protocol for:

Content

1. FAVOR III China trial Protocol Version 1.0 Sep.03.2018

2. FAVOR III China trial Protocol Version 1.3 Dec.05.2019

3. Detailed Summary of Protocol Changes and Rationale

4. Statistical Analysis Plan

FAVOR III China trial Protocol Version 1.3 Dec.05.2019

Comparison of Quantitative **F**low Ratio Guided and **A**ngiography Guided Percutaneous Inter**V**ention in Patients with C**OR**onary Artery Disease: FAVOR III China

Protocol Code：CRFH20180065

Version：V 1.3

Data：2019/12/05

Sponsor: Fuwai hospital, Chinese Academy of Medical Sciences

Address: No. 167, Beilishi Road, West district, Beijing

Primary investigator: Dr. Qiao Shubin, Dr. Xu Bo

Cooperation agency：Shanghai Jiaotong University-Pulse Medical Imaging Technology Joint Laboratory

Health Economics Analysis: School of Public Health, Fudan University

Statistic management: Medical Research and Biometrics Center, National Center for Cardiovascular Diseases (NCCD)

The information contained in this document is confidential and the proprietary property of the sponsor. Unauthorized use or disclosure of the information without the prior written consent of the sponsor is prohibited.

Protocol Signature Page

The signature below constitutes the approval of this protocol and the attachments and provides the necessary assurances that this trial will be conducted according to all stipulations of the protocol, including all statements regarding confidentiality, and according to local legal and regulatory requirements and applicable regulations and ICH guidelines.

Version Date: December 05.2019

Printed Name of Principal Investigator.

Bo Xu, Shubin Qiao

Name of Facility

Fu Wai Hospital, Chinese Academy of Medical Sciences

National Center for Cardiovascular Diseases

Location of Facility (City, Country)

A 167, Beilishi Road, Xicheng District, Beijing, 100037, China

Signature of Principal Investigator

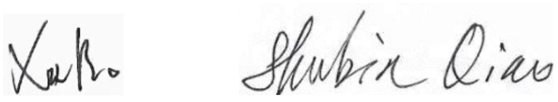

Table of Content

Protocol Signature Page 4

Table of Content 5

Clinical Trial Summary 9

1. List of the Abbreviations 13

2. Study Organization and Committee Members 15

3. Study Sites and Principal Investigators 16

4. Background and Rationale 18

5. Study Objective 21

6. Study Design 21

6.1 Study Flow 22

6.2 Study Population 23

6.3 Screen 23

6.4 Inclusion/Exclusion Criteria 24

6.4.1 Criteria Prior to Informed Consent (General criteria) 24

6.4.2 Criteria After Enrollment (Informed Consent) and Prior to Randomization 25

6.5 Randomization 25

6.6 Measures Taken to Avoid and Minimize Bias 26

6.7 Enrollment Time 26

6.8 The Expected Duration of the Trial 26

6.9 Follow-up 26

6.10 Subjects Number 27

7. Study Endpoints 27

7.1 Primary Endpoint 27

7.2 Major Secondary Endpoint 27

7.3 Other Secondary Endpoints 28

7.4 Endpoint Definitions 28

7.4.1 Acute Success 28

7.4.2 Death 29
7.4.3 Myocardial Infarction 30
7.4.4 Repeat Revascularization 32
7.4.5 Stent Thrombosis 33
7.4.6 PCI Strategy Changes After 3D-QCA...34
7.4.7 Health Economics Assessment Endpoints 34
8. Angiography and QFR Measurements 37
8.1 Angiography 37
8.2 QFR System 37
8.3 QFR Measurements 38
8.3.1 Measurement Principle 38
8.3.2 Analysis Procedure...38
9. Management Strategies...38
9.1 QFR-guided strategy (test) 38
9.2 Angiography-guided strategy (control)...39
9.3 Medical Therapy 39
9.3.1 Before PCI 39
9.3.2 During PCI 39
9.3.3 After PCI 40
9.4 PCI Procedure 41
10. Blinding 42
11. Monitoring 43
12. Statistical Consideration...44
12.1 Statistics Design (Study Hypothesis)...44
12.2 Sample Size 44
12.3 Number of Enrollments for Each Site...45
12.4 Dropout Rate 45
12.5 Analysis Population..46
12.6 Indicators and Methods of the Statistical Analysis 46
12.6.1 Demographic and Baseline Characteristics 46
12.6.2 Lesion and Treatment Information 47
12.6.3 Primary Endpoint 47

12.6.4 Secondary Endpoints 48
12.7 Missing, Unused or Erroneous Data 48
12.8 Statistic Analysis Plan 49
13. Prespecified Subgroups 49
14. Data Management 51
14.1 Fill-in of eCRF 51
14.2 Data Entry and Modification 51
14.3 Database Lock-up 51
15. Trial Termination or Withdrawal 51
16. Quality Control of the Trial 52
17. Ethical Consideration and Informed Consent 53
17.1 Ethical Consideration 53
17.2 Informed Consent Process 53
17.3 Responsibilities of the Investigator and IRB/EC 54
18. Regulations for Adverse Events 55
18.1 Adverse Events 55
18.2 Serious Adverse Events 55
18.3 Severity of Adverse Events 55
18.4 Records of Adverse Events 56
18.5 Adverse Events Reporting 56
19. Protocol Amendments 57
20. Direct access to source data and files 57
21. Insurance 58
22. Confidentiality Principle 58
23. Publication Policy 58
24. Responsibilities 58
25. Funding Source 59
References 60
Appendix 1. QFR Standard Operating Procedure 62
1. Coronary angiography acquisition 62
2. Angiographic image transfer 64
3. Angiographic run selection 64

4. Frame selection 66
5. Offset correction 67
6. Defining interrogated vessel segment 68
7. Checking the lumen contour 69
8. Defining corresponding points (optional) 70
9. Reference vessel 72
10. QFR computation 74
11. Analysis check 77
12. Documentation and report 78
Appendix 2. Blinding and Perception Analysis Questionnaire 80
Appendix 3. Investigator's Agreement 82

Clinical Trial Summary

**Title**	Comparison of Quantitative Flow Ratio Guided and Angiography Guided Percutaneous Intervention in Patients with Coronary Artery Disease: FAVOR III China
**Study Objectives**	The objective of the FAVOR III China trial is to assess the clinical outcomes and cost-effectiveness of a QFR-guided PCI strategy versus an angiography-guided PCI strategy in patients with coronary artery disease.
**Study Design**	FAVOR III China is a prospective, multi-center, subjects and clinical assessors blinded, sham controlled, superiority test, randomized trial. Participants meeting all the inclusion and exclusion criteria will be recruited and randomized in a 1:1 ratio to a QFR-augmented angiography-guided (QFR-guided) PCI strategy or a standard angiography-guided PCI strategy.
**Number of Subjects**	Approximately 3,830 subjects will be randomized
**Number of Sites**	Approximately 26 sites in China
**Study Population**	Adult patients scheduled for coronary angiography and are amenable to PCI treatment.
**Inclusion Criteria**	**Patients enrolled should meet all the following criteria:**<br>• **General inclusion criteria:**<br>− Age ≥ 18 years<br>− Stable, unstable angina pectoris, or post-acute MI (≥72 h)<br>− Able to understand the trial design and provide written informed consent<br>− Eligible for PCI by the operator assessment<br>• **Angiographic inclusion criteria:**<br>At least 1 lesion of 50%-90% (including) diameter stenosis in a coronary artery with ≥ 2.5mm reference vessel diameter by visual assessment
**Exclusion Criteria**	**Patients meeting any of the following criteria are excluded:**

	• **General exclusion criteria:**<br>− Cardiogenic shock or severe heart failure (NYHA ≥III)<br>− Severely impaired renal function: creatinine > 150 μmol/L or Cockcroft-Gault calculated GFR < 45 ml/kg/1.73 m^2<br>− Allergy to iodine-containing contrast agents which cannot be adequately premedicated<br>− Pregnancy or intention to become pregnant during the course of the trial<br>− Life expectancy less than 1 year<br>• **Angiographic exclusion criteria:**<br>− Patients with only 1 coronary artery lesion (DS%>90%) with TIMI flow <3<br>− An interrogated lesion is at the site of a myocardial bridge<br>− An interrogated lesion is a culprit lesion related to acute MI<br>− An interrogated lesion is in a bypass graft<br>− Poor angiographic image quality precluding vessel contour detection or with suboptimal contrast opacification<br>− Severe overlap in the stenosed segment or severe tortuosity of any interrogated vessel deemed not amenable to QFR measurement
**Primary Endpoint**	1-year rate of major adverse cardiovascular events (MACE), defined as a composite of all-cause mortality, any myocardial infarction (MI), or any ischemia-driven revascularization.
**Major Secondary Endpoint**	1-year rate of MACE excluding periprocedural MI, defined as a composite of all-cause mortality, spontaneous MI, or any ischemia-driven revascularization.
**Other Secondary Endpoints**	• The rate of MACE at 1 month, 6 months, 2 years, and 3 years.<br>• The rate of death (cardiovascular, non-cardiovascular and undetermined) at 1 month, 6 months, 1 year, 2 years, and 3 years.

	• The rate of MI (target vessel related and non-target vessel related) at 1 month, 6 months, 1 year, 2 years, and 3 years.<br>• The rate of target vessel revascularization (TVR) (ischemia driven and non-ischemia driven) at 1 month, 6 months,1 year, 2 years, and 3 years.<br>• The rate of any coronary artery revascularization (ischemia driven and non-ischemia driven) at 1 month, 6 months,1 year, 2 years, and 3 years.<br>• The rate of definite / probable stent thrombosis during acute, sub-acute, late, and very late phase according to the Academic Research Consortium (ARC)-2 at 1 month, 6 months,1 year, 2 years and 3 years.<br>• PCI strategy changes following QFR and three-dimension quantitative coronary angiography (3D-QCA).<br>• Cost-effectiveness endpoints at 1 month, 6 months, and 1 year.
**Technology of Interest**	We hypothesize that QFR (a simple-to-implement computational coronary physiology technique) guided PCI strategy, as compared with a standard angiography-guided PCI strategy, will:<br>• Avoids unnecessary intervention, reducing periprocedural MI and subsequent stent-related adverse events.<br>• Identifies angiographic borderline lesions that are functionally significant, the treatment of which reduces long-term ischemic events (spontaneous MI and ischemia driven revascularization).
**Technology of Reference**	Standard angiography guided PCI
**Study Period**	All subjects are planned to be enrolled within 1 year with a 3-year follow-up.
**Statistical Consideration**	A sample size of approximately 3,830 randomized subjects (1,915 in each group) is expected to provide more than 85% power to detect the superiority in the primary endpoint of 1-year MACE in QFR-guided group as compared with angiography-guided group. Meanwhile, 3,830 subjects would provide more than 80% power to demonstrate superiority in the major secondary endpoint as MACE excluding periprocedural MI.

	Primary and major secondary endpoints will be analyzed in intention to treat (ITT), per protocol set (PPS), and as treated set (ATS) (if applicable). The principal analyses of the primary and secondary endpoints will be performed by ITT.

1. List of the Abbreviations

Abbreviations	Definition
ACL	Angiographic core laboratory
ACS	Acute coronary syndrome
AE	Adverse event
ATS	As treated set
CABG	Coronary artery bypass graft
CAD	Coronary artery disease
CEC	Clinical event committee
eCRF	Electronic case report form
DSMB	Data safety monitoring board
EDC	Electronic data capture
eGFR	Estimated glomerular filtration rate
EQ-5D	Self-reported generic preference-based measure of health, developed by the EuroQol Group
EQOL	Economic and quality of life
FFR	Fractional flow reserve
FPFV	First visit for the first patient
LVEF	Left ventricular ejection fraction
MACE	Major adverse cardiovascular events
MI	Myocardial infarction

ICH	International Conference on Harmonization
ITT	Intention-to-treat set
IVUS	Intravascular ultrasound
OMT	Optimal medical therapy
PCI	Percutaneous coronary intervention
PI	Principal Investigator
PPS	Per-protocol set
QCA	Quantitative coronary angiography
QFR	Quantitative flow ratio
QFR-guided	QFR-augmented angiography-guided
SAE	Serious adverse events
SOP	Standard operation procedure
TLR	Target Lesion Revascularization
TVR	Target Vessel Revascularization

2. Study Organization and Committee Members

Primary Investigator	Shubin Qiao, MD; Bo Xu, MBBS
Steering Committee	Martin B. Leon, MD (Co-Chair); Shubin Qiao, MD; Lei Song, MD; Shengxian Tu, PhD; Bo Xu, MBBS (Co-Chair)
International Advisory Board	Javier Escaned, MD; William F. Fearon, MD; Ajay J. Kirtane, MD; Patrick W. Serruys, MD; Gregg W. Stone, MD (Chair); William Wijns, MD; Stephan Windecker, MD
Technological Cooperative Organization	Shanghai Jiao Tong University-Pulse Medical Imaging Technology Joint Laboratory; Shengxian Tu, PhD (Chair)
Health Economics Center	School of Public Health, Fudan University, Shanghai, China; Di Xue, PhD (Chair)
Clinical Events Committee	Jing Li, MD; Jian Liu, MD (Chair); Zhujun Shen, MD; Yingjia Xu, MD; Lilei Yu, MD
Data Safety and Monitoring Board	Xianghua Fu, MD; Jianping Li, MD; Wei Li, PhD; Yue Li, MD; Changsheng Ma, MD (Chair)
Angiographic core Laboratory	Core Medical (Beijing, China) Co., Ltd
Data Monitoring	Core Medical (Beijing, China) Co., Ltd
Data Management Center	IWRSMED Science and Technology (Beijing, China) Co., Ltd
Statistical Analysis Center	Medical Research and Biometrics Center, National Center for Cardiovascular Diseases, Beijing, China; Yang Wang, MSc (Chair)

3. Study Sites and Principal Investigators

No	Institute	City	Site Principal Investigators
1	Fu Wai Hospital, Chinese Academy of Medical Sciences	Beijing	Shubin Qiao, MD
2	Peking University Third Hospital	Beijing	Lijun Guo, MD
3	Chinese PLA General Hospital	Beijing	Yundai Chen, MD
4	Beijing Anzhen Hospital, Capital Medical University	Beijing	Yujie Zhou, MD
5	Beijing Tian Tan Hospital, Capital Medical University	Beijing	Zening Jin, MD
6	Affiliated Tongji Hospital of Tongji University	Shanghai	Xuebo Liu, MD
7	Affiliated Renji Hospital of Shanghai Jiao Tong University School of Medicine	Shanghai	Jun Pu, MD
8	Shanghai Sixth People's Hospital, Shanghai Jiao Tong University	Shanghai	Chengxing Shen, MD
9	Huadong Hospital, Fudan University	Shanghai	Xinkai Qu, MD
10	Affiliated East Hospital of Tongji University	Shanghai	Qi Zhang, MD
11	The Second Affiliated Hospital of Zhejiang University School of Medicine	Hangzhou	Jian’an Wang, MD
12	Affiliated Sir Run Run Shaw Hospital, Zhejiang University School of Medicine	Hangzhou	Guosheng Fu, MD
13	Nanjing First Hospital, Nanjing Medical University	Nanjing	Fei Ye, MD
14	Xuzhou Third People's Hospital	Xuzhou	Yaojun Zhang, MD

15	Fujian Medical University Union Hospital	Fuzhou	Lianglong Chen, MD
16	Guangdong General Hospital	Guangzhou	Junqing Yang, MD
17	The First Affiliated Hospital of Xi’an Jiaotong University	Xi’an	Ning Guo, MD
18	The Second Affiliated Hospital of Harbin Medical University	Harbin	Bo Yu, MD
19	Beijing Friendship Hospital, Capital Medical University	Beijing	Rongchong Huang, MD
20	Tianjin Medical University General Hospital	Tianjin	Qing Yang, MD
21	Hunan Provincial People's Hospital	Changsha	Hongwei Pan, MD
22	Jiangsu Provincial People's Hospital	Nanjing	Xiangqing Kong, MD
23	The Fourth Affiliated Hospital of China Medical University	Shenyang	Yuanzhe Jin, MD
24	The First Hospital of Shijiazhuang	Shijiazhuang	Jie Mi, MD
25	Union Hospital, Tongji Medical College, Huazhong University of Science and Technology	Wuhan	Xiang Cheng, MD
26	Wuhan Asia Heart Hospital	Wuhan	Xi Su, MD

4. Background and Rationale

For patients with coronary artery disease (CAD) showing symptoms of myocardial ischemia, coronary revascularization can eliminate myocardial ischemia and improve patient outcomes. Accurate identification of ischemia-related vessels or lesions is critical for patients undergoing percutaneous coronary intervention (PCI). Although routine non-invasive examination techniques including exercise electrocardiography test, stress echocardiography, and myocardial perfusion imaging are capable of identifying myocardial ischemia, precise localization of culprit lesions is still limited. Therefore, standard coronary angiography remains the widely used guidance for PCI in clinical routine. The main limitation of angiography is that it projects the 3-dimension vessel onto a 2-dimension plane, which can result in underestimation or overestimation of coronary stenosis. In addition, it cannot identify the stenosis that actually leads to ischemia. Intracoronary optical coherent topology (OCT) and intravascular ultrasound (IVUS) present a precise analysis of plaque characteristics, vulnerability, and lesion severity, thus improving the accuracy in identifying stenosis in combination with angiography, however, the identification of ischemia-related lesion remain limited.

Fractional flow reserve (FFR) is an invasive examination technique that measured via an intracoronary pressure wire placed in the distal end of the stenosis, defined as the mean distal coronary pressure divided by the mean proximal coronary pressure during hyperemia. DEFER study (Determine the Appropriateness of Angioplasty in Moderate Coronary Stenosis) investigated 352 patients of single-vessel intermediate coronary artery stenosis and demonstrated that coronary pressure-derived FFR could identifies those who will benefit from interventional revascularization [1]. The FAME study (Fractional Flow Reserve Versus Angiography for Guiding Percutaneous Coronary Intervention) enrolled 1,005 cases showing that routine measurement of FFR in patients with multivessel CAD who are undergoing PCI with drug-eluting stents (DES) significantly reduces the rate of the composite endpoint of death, nonfatal MI, and repeat revascularization at 1 year, with fewer stents and contrast usage, lower cost and less in-hospital time [2]. The FAME 2 study (Fractional Flow Reserve-Guided PCI Versus Medical Therapy in Stable Coronary

Disease) reported that in patients with stable CAD and functionally significant stenoses, FFR-guided PCI plus the best available medical therapy, as compared with the best available medical therapy alone, decreased the need for urgent revascularization. In patients without ischemia, the outcome appeared to be favorable with the best available medical therapy alone [3]. Based on these randomized clinical trials, European Society of Cardiology stated that FFR guided PCI as a class I recommendation (evidence level A) for myocardial revascularization[4].

Serial studies published in EuroPCR 2018 further demonstrated the value of coronary physiology. ORBITA study (Objective Randomized Blinded Investigation With Optimal Medical Therapy of Angioplasty in Stable Angina) randomized 196 patients who had stable angina with angiographically severe single-vessel coronary disease, and demonstrated that patients in PCI group, lower FFR / iFR before PCI had greater stress echocardiographic improvement [5]. The 5-year results of FAME 2 study further confirmed that for patients with stable CAD, an initial FFR-guided PCI strategy was associated with a significantly lower rate of the primary composite endpoint of death, myocardial infarction (MI), or urgent revascularization at 5 years than medical therapy alone. Patients without hemodynamically significant stenoses had a favorable long-term outcome with medical therapy alone [6]. The 10-year follow-up results of SCAAR study (Swedish Coronary Angiography and Angioplasty Registry) demonstrated that FFR-guided PCI showed superiority in long-term death, restenosis, and stent thrombosis compared with angiography-guided PCI [7]. Meta-analysis of three available randomized trials including FAME 2, DANAMI-3-PRIMULTI and COMPARE-ACUTE, demonstrated that FFR-guided PCI resulted in a reduction of the composite of cardiac death or MI compared with medical therapy, which was driven by a decreased risk of MI [8]. These critical findings and other recently reports underline the superiority of FFR/iFR-guided PCI over angiography-guided PCI in reduction of long-term death rate, restenosis rate and stent thrombosis.

Nonetheless, guidewire-based physiologic indices are still largely underused in practice [8-10] due to the concerns for prolonged procedural time, increased costs,

potential complications by pressure wire instrumentation, lack of training, and the belief (not supported by evidence) that angiography is the criterion standard. For these reasons, coronary angiography remains the predominant method for decision making for coronary revascularization in many centers across the world, including those located in emerging countries with limited health care resources.

Quantitative flow ratio (QFR) is a novel method assessing the physiologically significance of coronary artery stenosis. It enables fast computation of FFR by 3-dimensional reconstruction of coronary artery and computational fluid dynamics based on angiography, without the use of pressure wire or adenosine. FAVOR pilot study (Diagnostic Accuracy of Fast Computational Approaches to Derive Fractional Flow Reserve From Diagnostic Coronary Angiography) confirmed the feasibility of QFR in offline fashion. Accuracy of QFR in identifying hemodynamically significance of intermediate coronary stenosis was 86% with compared with invasive FFR, which was higher than that of quantitative coronary angiography (QCA) [11]. Subsequently, the FAVOR II studies (Diagnostic Accuracy of Angiography-Based Quantitative Flow Ratio Measurements for Online Assessment of Coronary Stenosis) carried out in China, Japan and Europe, aimed to assess the accuracy of online QFR in identifying physiologically significant stenosis. FAVOR II China [12] enrolled 308 cases of patients with at least one lesion with 30%-90% diameter stenosis (DS) by visual assessment and performed online QFR, online QCA and FFR measurements. Mean time for QFR measurement was 4.36±2.55 min, and the per-patient and per-vessel accuracy of QFR was 92.4% and 92.7%, respectively, with FFR as the reference standard. FAVOR II Europe Japan enrolled 310 patients and showed that sensitivity and specificity by QFR was significantly higher than those by QCA (sensitivity 87% vs. 44%, p<0.001; specificity 87% vs. 77%, p=0.002).

The aforementioned studies demonstrated the feasibility and accuracy of QFR in outlining functional stenosis severity, potentially enabling broader clinical applications. To date, however, there has been no prospective, randomized clinical trial comparing a QFR-augmented angiography-guided (QFR-guided) strategy with guidance based on angiography-only (angiography-guided) in terms of clinical outcomes and cost-effectiveness. We are therefore performing the pivotal

randomized FAVOR (Comparison of Quantitative Flow Ratio Guided and Angiography Guided Percutaneous Intervention in Patients with Coronary Artery Disease) III China trial.

5. Study Objective

The study objective is to assess the clinical outcomes and cost-effectiveness of a QFR-guided PCI strategy versus an angiography-guided PCI strategy in patients with CAD.

6. Study Design

The FAVOR III China is a prospective, multi-center, subjects and clinical assessors blinded, sham controlled, superiority test, randomized clinical trial. Eligible patients meeting all the inclusion and exclusion criteria will be recruited, and then 1:1 randomly assigned to either QFR-guided PCI strategy (test group) or angiography-guided PCI strategy (control group). In order to avoid the influence of subjective deviation between the executor and subjects on the test results, a common QFR calculation time is set in both groups. If the patient is assigned to QFR-guided PCI, QFR is measured in all coronary arteries with DS% ≥50% and ≤90% and ≥2.5mm reference vessel diameter. PCI treatment is performed in all lesions with QFR ≤0.80 and is not performed in lesions with QFR >0.80. If the patient is assigned to angiography-guided PCI strategy, the investigator will perform PCI according to the stenosis severity based on visual assessment of the angiogram following local standards of practice. The primary endpoint was 1-year rate of MACE, defined as a composite of all-cause death, all MI and any ischemia-driven revascularization. The major secondary endpoint was 1-year rate of MACE excluding perioperative MI, defined as a composite of all-cause death, spontaneous MI and any ischemia-driven revascularization at 1 year. All patients will be followed-up at 1 month, 6 months, 1 year, 2 years and 3 years. The blinding will be maintained until completion of the 1-year follow-up visit for all randomized subjects.

6.1 Study Flow

- Screening will be conducted in 2 phases. First, patients with CAD scheduled for coronary angiography are approached for consent after discussion of the trial design.
- Patients meeting all general inclusion/exclusion criteria who provide written informed consent will be enrolled.
- Second, coronary angiography is performed with standard projections with minimal overlap and foreshortening in each interrogated vessel. Enrolled patients will then be screened for final angiographic inclusion and exclusion criteria.
- Patients meeting all general and angiographic criteria are then randomly assigned in a 1:1 ratio to a QFR-guided strategy (test group) or an angiography-guided strategy (control group).
- If the patient is assigned to the QFR-guided strategy, QFR is first measured in all coronary arteries with any lesion with DS% ≥50% and ≤90% in which the reference vessel diameter is ≥2.5 mm. PCI treatment is performed in all lesions with QFR ≤0.80 and is not performed in lesions with QFR >0.80. No PCI is performed in patients with QFR >0.80 in all interrogated vessels; such patients are treated with optimal medical therapy (OMT) alone.
- The angiogram files for both groups will be saved for subsequent offline analysis and validation by an independent angiographic core laboratory (ACL).
- Follow up: all patients will be followed by either telephone or clinic visit at 1 month (30 days ± 7 days), 6 months (180 days ± 30 days), 1 year (365 days ± 30 days), 2 years (730 days ± 30 days), and 3 years (1095 days ± 30 days). Each patient will complete a quality-of-life EuroQol Five-Dimensional (EQ-5D) questionnaire at baseline, 1 month, 6 months, and 1 year. Clinical costs will be prospectively recorded at 1 month, 6 months, and 1 year.
- Study flowchart

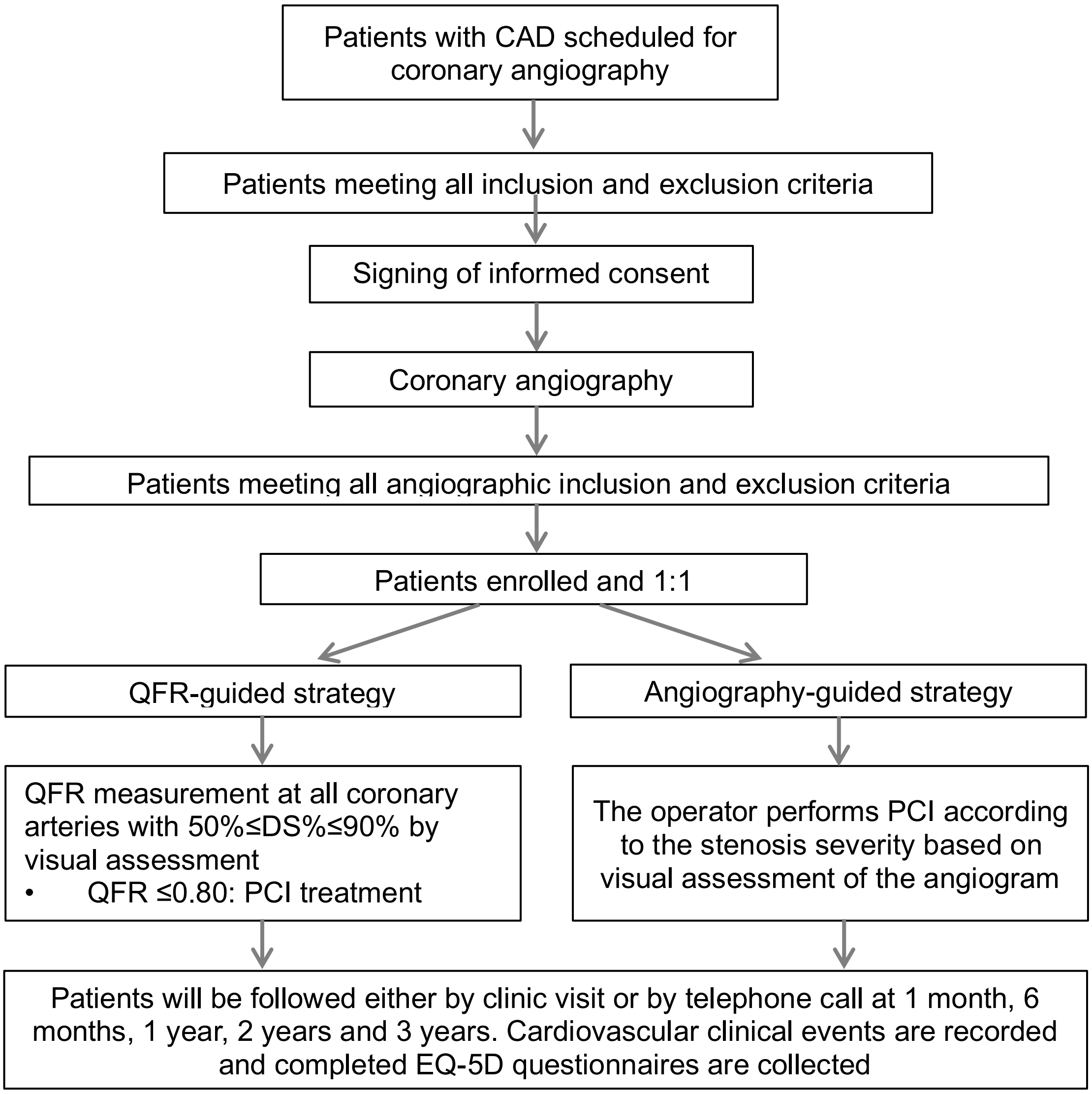

6.2 Study Population

The study population consists of adult subjects scheduled for coronary angiography with planned or possible PCI, including patients with stable or unstable angina pectoris, or post–acute MI (≥72 hours). Patients must have at least 1 lesion of 50% - 90% diameter stenosis in a coronary artery with ≥2.5 mm reference vessel diameter by visual assessment and be eligible for DES implantation as determined by the investigators.

6.3 Screen

Screening will be conducted in 2 phases. First, patients with CAD scheduled for coronary angiography are approached for consent after discussion of the trial design

by trained research staff. Patients meeting all general inclusion/exclusion criteria who provide written informed consent will be enrolled. All patients will provide signed and dated informed consent approved by the Ethics Committee (EC) of each site. Second, coronary angiography is performed with standard projections with minimal overlap and foreshortening in each interrogated vessel. Enrolled patients will then be screened for final angiographic inclusion and exclusion criteria.

6.4 Inclusion/Exclusion Criteria

Enrolled patients should meet all the following inclusion/exclusion criteria:

6.4.1 Criteria Prior to Informed Consent (General criteria)

Patients with CAD scheduled for coronary angiography are approached for consent after discussion of the trial design. Enrolled patients should meet all the following inclusion and exclusion criteria in general.

Inclusion (pre informed consent)

1. Age ≥ 18 years.
2. Stable or unstable angina pectoris, or with post-acute MI (≥ 72 hours).
3. Able to understand the trial design and provide written informed consent.
4. Eligible for PCI by operator assessment.

Exclusion (pre informed consent)

1. Cardiogenic shock or severe heart failure (NYHA ≥III).
2. Severely impaired renal function: creatinine >150μmol/L or Cockcroft-Gault calculated GFR <45 ml/kg/1.73 m^2 (calculated with Cockcroft-Gault formula).
3. Allergy to iodine-containing contrast agents which cannot be adequately premedicated.
4. Pregnancy or intention to become pregnant during the course of the trial.

5. Life expectancy less than 1 year.

6.4.2 Criteria After Enrollment (Informed Consent) and Prior to Randomization

Participants who provide informed consent and are clinically eligible will undergo coronary angiography assessments. Participants meeting the angiographic inclusion criteria, and not meeting any of the following angiographic exclusion criteria will be randomized.

Angiographic inclusion criteria

At least 1 lesion of 50%-90% diameter stenosis in a coronary artery with ≥ 2.5mm reference vessel diameter by visual assessment.

Angiographic exclusion criteria

1. Patients with only 1 coronary artery lesion with DS >90% with TIMI flow <3.
2. An interrogated lesion is at the site of a myocardial bridge.
3. An interrogated lesion is a culprit lesion related to acute MI.
4. An interrogated lesion is in a bypass graft.
5. Poor angiographic image quality precluding vessel contour detection or with suboptimal contrast opacification.
6. Severe overlap in the stenosed segment or severe tortuosity of any interrogated vessel deemed not amenable to QFR measurement.

6.5 Randomization

Patients meeting all clinical and angiographic entry criteria are then randomly assigned in a 1:1 ratio to a QFR-guided strategy (test group) or an angiography-guided strategy (control group) via an Internet-enabled Web-based response system (IWRS) in block sizes of 6. Randomization is stratified by center, the presence of diabetes mellitus, single versus multivessel disease, and the existence of any vessel with DS% >90% with TIMI flow <3. The lesions intention-to-treat must be declared by

the interventional team and are recorded in the patient case record form prior to randomization in both arms.

6.6 Measures Taken to Avoid and Minimize Bias

Multicenter, randomized sham-controlled design with powered sample size avoids the select bias between the two groups. The subjects and clinical assessors blinding design avoids the impact of subjective deviation between executor and subjects. Before the trial initiation, all QFR analysts should be systematically trained and have extensive experience in QFR analysis to minimize the inter-observer and intra-observer variability. All reported adverse events will be reviewed and adjudicated by the independent clinical events committee (CEC), consisting of independent experts in the cardiovascular field who are not participating in this trial.

6.7 Enrollment Time

It is planned that all subjects will be enrolled within approximate 12 months after the trial begins.

6.8 The Expected Duration of the Trial

The overall expected duration is about 5 years. The time of clinical trial documents preparation and improvement including clinical trial protocol, investigator's manual, informed consent, Electronic case report form (eCRF) will take approximate 2 months; ethical approval - approximate 3 months; subjects enrollment - approximate 1 year; follow-up - 3 years; data management, statistical analysis and report the results - approximate 3 months.

6.9 Follow-up

Follow-up is performed at prespecified time points by phone call or out-patient visits at 1 month, 6 months, 1 year, 2 years and 3 years after the index procedure. The table 1 lists the visit windows for endpoint and follow-up rate analysis.

Table 1. Visit Windows for Endpoint and Follow-up Rate Analysis

Follow-up interval	Study Time Window Post Procedure for endpoint	Study Time Window Post Procedure for follow-up rate
1 Month	30 days	30 ± 7 days
6 Month	180 days	180 ± 30 days
1 Year	365 days	365 ± 30 days
2 Years	730 days	730 ± 30 days
3 Years	1095 days	1095 ± 30 days

6.10 Subjects Number

Approximate 3,830 patients (1,915 in each group) are required in this trial.

7. Study Endpoints

7.1 Primary Endpoint

The primary endpoint is the 1-year rate of major adverse cardiovascular events (MACE), defined as a composite of all-cause mortality, any MI, or any ischemia-driven revascularization.

7.2 Major Secondary Endpoint

The major secondary endpoint is 1-year MACE excluding periprocedural MI. defined as all-cause mortality, spontaneous MI and any ischemia-driven revascularization.

7.3 Other Secondary Endpoints

- Acute success, including lesion success (lesion level) and procedural success (patient level)
- MACE (at 1 month, 6 months, 2 years, and 3 years)
- Death (at 1 month, 6 months, 1 year, 2 years and 3 years)
- MI (at 1 month, 6 months, 1 year, 2 years and 3 years)
- Target vessel revascularization (TVR) at 1 month, 6 months,1 year, 2 years and 3 years
- Any coronary artery revascularization at 1 month, 6 months,1 year, 2 years and 3 years
- Stent thrombosis (definite and probable) during acute, sub-acute, late, and very late phase according to the Academic Research Consortium (ARC)-2 [13] at 1 month, 6 months,1 year, 2 years and 3 years
- PCI strategy changes after three-dimension quantitative coronary angiography (3D-QCA): comparing the changes on the strategy including devices size after 3D-QCA guided versus angiography guided PCI
- Cost-effectiveness assessments at 1 month, 6 months, 1 year.

7.4 Endpoint Definitions

7.4.1 Acute Success

- Lesion success at lesion level, defined as residual stenosis less than 30% for patients treated with stents or 50% for patients treated with drug coated balloon by visual estimation, with TIMI flow grade 3, in the treated vessel.
- Procedural success at patient level, defined as attainment of lesion success in all the treated lesions, together with the absence of any in-hospital major adverse cardiac events (maximum of 7 days, including all-cause death, MI or any revascularization).

7.4.2 Death

- **All-cause death**, including cardiovascular death, non-cardiovascular death and undetermined death according to the ARC-2 consensus [13].
- **Cardiovascular death**, defined as death resulting from cardiovascular causes. The following categories may be collected:
  1) Death caused by acute MI
  2) Death caused by sudden cardiac, including unwitnessed, death
  3) Death resulting from heart failure
  4) Death caused by stroke
  5) Death caused by cardiovascular procedures
  6) Death resulting from cardiovascular hemorrhage
  7) Death resulting from other cardiovascular cause
- **Non-cardiovascular death**, defined as any death that is not thought to be the result of a cardiovascular cause. The following categories may be collected:
  1) Death resulting from malignancy
  2) Death resulting from pulmonary causes
  3) Death caused by infection (sepsis included)
  4) Death resulting from gastrointestinal causes
  5) Death resulting from accident/trauma
  6) Death caused by other non-cardiovascular organ failure
  7) Death resulting from other non-cardiovascular cause

- **Undetermined death,** defined as a death not attributable to any other category due to the absence of any relevant source documents. Such deaths will be classified as cardiovascular death during endpoint determination.

7.4.3 Myocardial Infarction

The term of MI should be used when there is evidence of myocardial necrosis in a clinical setting. The definition of MI is defined and classified as follows (see Table 2):

Table 2. The Definitions of Myocardial Infarction

**Categories**	**Patients**	**Criteria**
Periprocedural MI *	Stable patients<br><br>(CK-MB normal)	CK-MB > 3x ULN
	Patients with acute coronary syndrome (CK-MB elevation)	If the CK-MB is stable or falling:<br><br>>3x CK-MB increment above the baseline level;<br><br>If the peak value has not been reached<br><br>Additional >3x CK-MB increment above the baseline level, with either new EKG changes or persistent angiographic complications.<br><br>* If for some reason CK-MB is not available, then a troponin elevation of >21x ULN will be

		used.
**Spontaneous MI**	Events occurring after the first 48 hours beyond the index PCI	cTn > ULN or CK-MB > 99th percentile URL (or ULN) with at least one of the following conditions:<br>- Symptoms of ischemia;<br>- ECG changes indicative of new ischemia (new ST-T changes or new LBBB);<br>- Development of pathological Q-waves in the ECG;<br>- Image evidence of new loss of viable myocardium or new regional wall motion abnormalities.
There must be data on preoperative baseline CK-MB level and assumed that it typically rises and falls after surgery to diagnose peri-procedural MI. CK-MB = creatine kinase-MB, cTn = cardiac troponin, LBBB = Left bundle branch block, MI = myocardial infarction, ULN = upper limit of normal (ULN values will be collected from each hospital laboratory before the trial).		

- **Definitions of subtype of myocardial infarction**

1) Periprocedural MI after PCI: The periprocedural period refers to the first 48 hours after PCI.

2) Periprocedural MI after CABG: The periprocedural period refers to 7 days after CABG.

3) Spontaneous MI: Post-periprocedural MI may be secondary to late stent

complications or the progression of primary disease. In most cases, ECG and angiography can lead to classification of the MI as target-vessel-sourced or non-target-vessel-sourced. Given this unique problem and the pathophysiological mechanisms behind the effect of those events on short-term and long-term prognosis, a more sensitive definition should be used instead of the one for periprocedural MI, which is defined as any increase of cTn or CK-MB that exceeds 99th percentile of ULN (ULN can be used if URL is missing). All late events that are not related to revascularization will be considered as spontaneous MI.

4) For each MI adjudicated by the CEC, the type will also be described as:

Q-wave MI: Appearance of new pathological Q waves (≥ 40 ms in duration and ≥ 1 mm in depth) in ≥2 contiguous leads, with or without postprocedural CK or CK-MB levels higher than normal levels.

Non-Q wave MI: All MIs not classified as Q-wave MI.

5) MI can also be adjudicated based on the relationship with the target vessel: all MI which cannot be attributed to blood vessels other than the target vessels will be considered as target vessels related.

- Laboratory tests

  The CK-MB and cardiac troponin (cTnI or cTnT) levels will be measured in all patients at baseline, 6 to 12 hours, and 18 to 24 hours after PCI is completed. If there is elevation of CK-MB or cardiac troponin levels, total CK-MB or cardiac troponin levels should be remeasured 24 to 48 hours after PCI is completed.

7.4.4 Repeat Revascularization

- **Planned Revascularization,** defined as revascularization planned at the time of the first PCI procedure after patient inclusion, based on the results of angiography and functional testing. Planned revascularization could be performed at the first PCI procedure, within 60 days after first PCI or at the

time decided by the investigators. Planned revascularization will not be considered as an endpoint.

- **Unplanned Revascularization,** defined as revascularization not performed as part of standard care during the first PCI procedure, or it is not planned as a staged procedure to occur within 60 days or at the time decided by the investigator. Unplanned revascularization is PCI or CABG treatment based on persisting symptoms of ischemia. For unplanned revascularization, both target vessel revascularization (TVR) and target lesion revascularization (TLR) should be evaluated carefully.

- **Target Vessel Revascularization (TVR),** defined as any repeat PCI or surgical revascularization of any segment of the target vessel. All target vessel revascularization events are classified by investigators into clinically indicated or non-clinically indicated. The target vessel is defined as all vessel segments of the main epicardial vessel proximal and/or distal to the target lesion, inclusive of all branches upstream and downstream of the target lesion as well as the target lesion itself.

- **Ischemia-driven (ID) Revascularization,** defined as ischemia-driven if it is associated with any of the following conditions:

  - ACL-reported QFR or site-reported FFR ≤0.80 or iFR ≤0.89;

  - Patients with ischemic symptom or positive non-invasive functional diagnosis result, whose angiography delineated a diameter stenosis ≥50% (based on the average of multiple views QCA result);

  - Patients without ischemic symptom or positive non-invasive functional diagnosis result, whose angiography delineated a diameter stenosis ≥70% (based on the average of multiple views QCA result).

7.4.5 Stent Thrombosis

Stent thrombosis is stipulated according to the ARC-2 definition [13] and further classified into definite thrombosis and probable stenosis.

- **Definite stent thrombosis,** defined as occlusion in the stenting site caused by thrombosis confirmed by angiographic or other pathological evidence and accompanied by any of the following criteria:
  - Presence of an acute myocardial ischemia symptom;
  - Ischemic cardiogram changes;
  - Elevated myocardial enzymes.
- **Probable stent thrombosis,** defined as conditions including any of the following:
  - Any unexplained sudden death occurring 30 days after PCI;
  - Any acute MI related to acute ischemia in the target vessel but without angiographic confirmation for thrombosis;
  - Any acute MI related to any other clear causes.

Stent thrombosis can also be classified into acute thrombosis (occurring 24 h after PCI), subacute thrombosis (occurring 1-30 days after PCI), late thrombosis (occurring 31d-1 year after PCI), or very late thrombosis (occurring more than 1 year after PCI).

7.4.6 PCI Strategy Changes After 3D-QCA

3D-QCA-based device sizing is recommended in the QFR-guided arm. The device sizes selected by the operator will be recorded before and after 3D-QCA measurements or angiographic visual evaluation, to compare the changes on the size decision between the two groups.

7.4.7 Health Economics Assessment Endpoints

The cost and effectiveness of the QFR-guided PCI strategy and the standard angiography-guided PCI strategy is collected to compare the economics of the two intervention strategies.

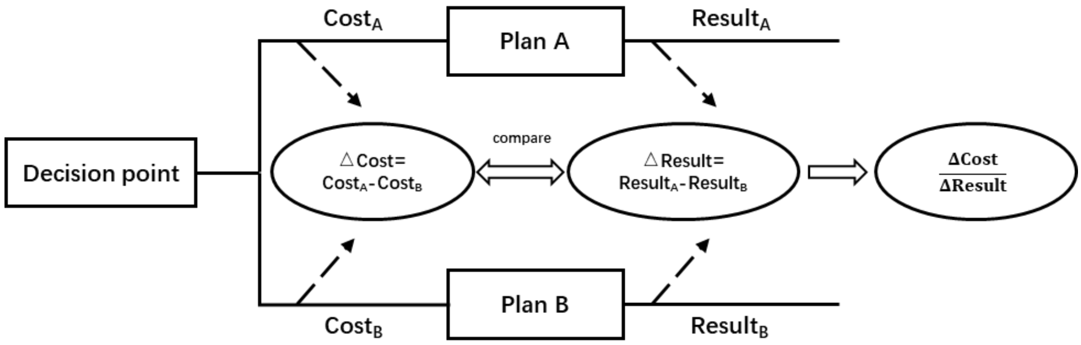

- **Cost-utility analysis**

Utility is evaluated by quality-adjusted-life-years (QALYs) index transformed by Japanese TTO formula. EQ-5D in official Chinese version is adopted for QALY data collection. Cost indicators include:

1) Initial hospitalization related expenses, including:

   a) Interventional treatment expenses: include expenses on high-value consumables (stent, balloon, guide wire, guide catheter, micro-catheter, etc.), other consumables, examination/treatment expenses(coronary angiography, percutaneous coronary intervention, intraluminal imaging and other examination/treatment), intraoperative medication (contrast agent, nitroglycerin and other medication), care and other items; total interventional treatment expenses refer to sum of all expenses mentioned above.

   b) Ward related expenses: expenses on ward medication, examination (imaging, laboratory test and other examination), treatment, consumables, care and other items; all said expenses constitute total ward related costs during initial hospitalization.

   c) Other expenses during initial hospitalization.

   d) Total expenses during initial hospitalization: sum of Interventional treatment expenses, ward related expenses and other expenses during initial hospitalization.

2) Main cardiovascular medication expenses at 1 month, 6 month and 1 year: expenses on aspirin, clopidogrel, other antiplatelet drugs, statins, angiotensin converting enzyme/angiotensin II receptor blocker, beta receptor blocker, calcium channel blocker and nitrates; total main cardiovascular medication costs at 1 month, 6 month and 1 year.

3) Outpatient and/or hospitalization expenses associated with MACE at 1 month, 6 month and 1 year, including:

   a) Outpatient related expenses: include expenses on registration, examination, treatment, consumables, medicine and other items; all said expenses constitute total outpatient expenses.

   b) Interventional treatment expenses: the composition is the same as listed above.

   c) Ward related expenses: the composition is same as listed above.

   d) Other expenses.

   e) MACE related total medical expenses.

- **Effectiveness and utility indexes**

1) Effectiveness

   a) Incidence of PCI treatment, stent use per patient and average length of stay during initial hospitalization;

   b) Incidence of TVR (ischemia driven and non-ischemia driven) at 1 month, 6 month and 1 year;

   c) Incidence of any coronary artery revascularization (ischemia driven and non-ischemia driven) at 1 month, 6 month and 1 year.

2) Utility

   a) EQ-5D and QALYs during initial hospitalization;

b) EQ-5D and QALYs at 1 month, 6 month and 1 year

3) Safety

a) incidence of MACE during initial hospitalization;

b) Incidence of MACE at 1 month, 6 month and 1 year;

c) Incidence of death (cardiogenic, vascular-genic and non-cardiovascular-genic) at 1 month, 6 month and 1 year;

d) Incidence of MI (target vessel related and non-target vessel related) at 1 month, 6 month and 1 year;

e) Incidence of stent thrombosis defined by the ARC-2 at 1 month, 6 month and 1 year.

8. Angiography and QFR Measurements

8.1 Angiography

Patients enrolled in the study will perform coronary angiography according to standard operation procedure to acquire projections with minimal overlap in the target vessel. Two angiographic image runs with at least 25° deviation in projection angles are required. In both groups, to ensure that subjects are blinded, 10 minutes of QFR calculation time is reserved after coronary angiography and before interventional treatment.

8.2 QFR System

QFR is measured through AngioPlus system (Pulse Medical Imaging Technology, Shanghai Co., Ltd., Shanghai, China), consists of a display screen, a host and QFR measurement software, according to the standard operation procedure (Appendix 1). Two angiographic image runs with at least 25° deviation in projection angles are transferred through local network to AngioPlus system. After completing the QFR analysis, save the results for subsequent research.

8.3 QFR Measurements

8.3.1 Measurement Principle

The principle of QFR measurement system: The AngioPlus system receives two angiographic image runs with at least 25° deviation in projection angles, uses the centerline and contour detection algorithms to complete the delineation of the target vessel and 3D reconstruction automatically. The QFR value of the target vessel is obtained based on the TIMI frame count and optimized coronary hemodynamic model, and the analysis results can be summarized in the report to provide the doctor with auxiliary diagnosis and treatment information.

8.3.2 Analysis Procedure

The workflow of QFR measurement software is as follows：

Step1: Select a pair of calibration points with significant anatomical features to perform geometric position calibration on the two image sequences;

Step2: Use the verified centerline and contour detection algorithms to complete the delineation of the target vessel automatically, and combine the 3D reconstruction algorithm to reconstruct and display the target vessel and its reference lumen;

Step3: The analyst selects the image where the contrast enters the target vessel segment as the starting frame, and the contrast flowing out of the target vessel segment as the end frame. The QFR measurement system automatically calculates QFR value at each position of the target vessel and displays pull back curve;

9. Management Strategies

9.1 QFR-guided strategy (test)

If the patient is assigned to the QFR-guided strategy, QFR is first measured in all coronary arteries with any lesion with DS% ≥50% and ≤90% in which the reference vessel diameter is ≥2.5 mm. PCI treatment is performed in all lesions with QFR ≤0.80 and is not performed in lesions with QFR >0.80. No PCI is performed in

patients with QFR <0.80 in all interrogated vessels, such patients are treated with OMT alone. It is strongly recommended to select the device size based on the 3D-QCA measurements. In addition, a final QFR measurement is encouraged but not mandated in all QFR-guided patients after completion of PCI.

9.2 Angiography-guided strategy (control)

If the patient is assigned to the angiography-guided strategy (control), PCI is performed based on visual assessment of the angiogram following local standards of practice.

9.3 Medical Therapy

9.3.1 Before PCI

1) Aspirin 300mg, started at least 24 hours before the procedure;

2) Clopidogrel 300-600mg load, at least 6 hours before the procedure, followed by 75mg/d; if preoperative clopidogrel (75mg/d) has been taken for more than 72 hours, the loading dose will be not required.

3) Ticagrelor 180 mg load, at least 6 hours before the procedure, followed by 90mg twice daily; if preoperative ticagrelor (90 mg bid) has been taken for more than 72 hours, the load will be not required.

9.3.2 During PCI

Unfractionated heparin for intravenous injection before stent implantation at 100U/kg or by maintaining ACT level at 250-350 seconds (HemoTec method). For patients who have taken low molecular weight heparin (LMWH) prior to the procedure with sufficient amount subcutaneously injected for more than twice daily, if the last injection was within 8 hours, unfractionated heparin is not required; if the last injection was within 8-12 hours, intravenously supplemented of unfractionated heparin with half dosage will be required; if the last injection was conducted over 12 hours, the patients should be treated as without usage of LMWH. At this situation, unfractionated heparin or LMWH will be required with full dosage.

9.3.3 After PCI

- **For Patients Not Requiring PCI**

In subjects planned to receive OMT alone determined by QFR results, the recommended treatment is as follows:

1) Aspirin 75-100 mg/d (clopidogrel 75 mg/d or any other $P2Y_{12}$ receptor inhibitor, only if aspirin intolerant)

2) Metoprolol (or any other selective beta-1 receptor blocker) 25-100 mg/d.

   ± Calcium channel blocker

   ± Long-acting nitrates

3) Atorvastatin 20, 40 or 80 mg/d or other statin at similar potency according to the LDL-cholesterol levels (target level is <2.0 mmol/dL).

   ± Ezetimibe or niaspan

4) Lisinopril 5-10 mg/d or any other ACE-inhibitor or angiotensin receptor blocker at a therapeutic dosage with similar potency.

5) Optimal diabetes control (for diabetics).

- **For Patients Requiring PCI**

  − After PCI

1) LMWH: subcutaneous injection if necessary.

2) Clopidogrel 75 mg once daily or ticagrelor 90 mg twice daily.

3) Aspirin: 100-300 mg once daily.

  − After discharge

1) Aspirin: 100-300mg once daily, 100mg daily after 30 days for long-term use.

2) Clopidogrel 75 mg once daily or ticagrelor 90 mg twice daily for at least 12

months.

3) Other medical therapy is the same as medication for patients not requiring PCI.

9.4 PCI Procedure

In patients planned to receive PCI treatment determined by the operator or QFR results, a DES is recommended for revascularization. A bare metal stents (BMS) could be used only when the DES was not available. The PCI procedures were performed according to standard techniques following the clinical practice.

Other physiological assessments such as iFR or FFR cannot be used in either arm for further evaluation of the lesion before stenting; however, intravascular imaging is permitted as per operator discretion but should not be differentially applied. If necessary, PCI may be staged in patients with multivessel disease and completed within 60 days after the index procedure.

The special lesion specification:

- Long lesions: Long lesions should be treated according to routine clinical practice of the sites and a stent-to-stent overlap of 4mm is recommended.

- Bifurcation lesions: Bifurcation lesions should be treated according to routine clinical practice of the sites and a provisional one-stent technique is recommended.

- Ostial lesions: Ostial lesions should be treated according to routine clinical practice of the sites, and it is recommended that the stent fully cover the ostium and the minimum struts protruding into the aorta.

- Staged procedure: For subjects with multivessel disease who need staged PCI, the procedure should be completed within 60 days after the index procedure and documented in the eCRF.

10. Blinding

In the FAVOR III China trial, all the subjects and clinical assessors (including the follow-up research personnel, CEC, and ACL) will be blinded to randomization arm. All study site personnel will be trained not to disclose the treatment assignment to the subject at any unplanned time before trial initiation. In addition to standard procedural sedation, music-playing headphones will be worn by the patient during the whole procedure, and patients in both groups will undergo a preset 10-minute delay for QFR calculation before the PCI procedure, and a lesion/device evaluation form is required to be filled during said period in both groups, to reduce the possibility of unblinding. A common follow-up template will be used for subjects in both arms to minimize bias and maintain the blinding. The blinding will be maintained until completion of the 1-year follow-up visit for all registered subjects.

The operators and personnel involved in the PCI procedure will not be blinded and therefore will not take part in clinical follow-up. Clinical follow-up visits will be conducted by the site personnel blinded to the randomized results and using the same visit form. In addition, the QFR or QFR results are not allowed to be listed in any medical notes, billing information, or other related patient information, but instead should use “depending on evaluation during the procedure” or other non-revealing language to maintain the blind. The CEC and the Data Safety Monitoring Board (DSMB) will be blinded to the subject’s randomization. Independent statisticians will generate blinded tables for review by the DSMB. The DSMB may request unblinded data if a safety signal is observed. At post-procedure, 6 months and 1-year follow-up, subjects will be required to complete a blinding and perception analysis questionnaire (Appendix 2) to assess randomization concealment and the perception of treatment allocation. The blinding assessments will be reported in the primary endpoint article.

The study unblinded personnel will be the independent biostatisticians involved in generating and verifying the randomization code, clinical safety monitors, site monitors, clinical data management and information system personnel working on the trial. All these unblinded personnel will be trained not to disclose the treatment

assignment to the subject. Restricted access of blinded personnel to the clinical database will be maintained until unblinding of the study at one year.

11. Monitoring

Before starting this study, monitors need to confirm and collect trial protocol and amendments confirmed by the investigators, the EC and the clinical trial institutions.

Monitors of each clinical trial center should arrange training for trial-related personnel in the following aspects: clinical trial protocol, screening procedure, inclusion/exclusion criteria, informed consent procedure, reporting procedure of (serious) adverse events, completion and amendment guide for eCRF, information to be collected in the trial, monitoring scope and requirements. Meanwhile, it is necessary for monitors to hand over trial documents to trial center personnel, obtain laboratory qualification certificate, and inform the trial-related personnel of the requirements, frequency and necessary documents for monitoring during every visit.

Investigator of each center should maintain regular communication with monitor before starting enrollment. Once the first subject is enrolled, monitor shall be notified immediately to conduct the first visit for the first patient (FPFV). After FPFV, monitors should communicate with the clinical trial center at least once a week and inquiry the enrollment progress and related problems. Monitors may communicate with investigators through face-to-face interviews, telephone calls, text messages and/or e-mails. The frequency of communication can be adjusted according to the actual trial schedule.

Monitors should confirm that all data in case report forms are correctly recorded in line with source data, and all mistakes, omission have been corrected with signature of the corresponding investigator. Monitors should also ensure that basic information (such as gender, age etc.), therapy and evaluation result is all recorded correctly. A monitor report should be prepared after each monitor, which should include monitor name, monitor date and time, hospital, contents of monitor, investigators visited, trial progress, present problems, conclusion and corrective measures for mistakes and omission.

12. Statistical Consideration

12.1 Statistics Design (Study Hypothesis)

The objective of the FAVOR III China study is to investigate whether a strategy of QFR-guided PCI shows superior clinical outcome and cost-effectiveness compared with a strategy of standard angiography-guided PCI. Primary endpoint is the rate of 1-year MACE, and corresponding hypothesis is being tested:

H_0: P_T-$P_C \geq 0$;

H_1: P_T-$P_C < 0$;

where P_T and P_C denotes MACE at 1 year in QFR-guided PCI group and angiography-guided PCI group, respectively. The results of this clinical trial will be based on the difference of MACE at 1 year between QFR- and angiography-guided PCI group. If results show that the upper limit of 95% CI of the difference in MACE at 1 year between QFR-guided and angiography-guided group is below 0%, then the superiority conclusion is confirmed.

12.2 Sample Size

Sample size calculation is based on primary endpoint. The corresponding null hypothesis to be tested is that the difference of 1-year MACE between test group and control group (P_T-P_C) is equal or greater than 0, versus the alternative hypothesis is that the difference is less than 0.

H_0: P_T-$P_C \geq 0$;

H_1: P_T-$P_C < 0$;

where P_T and P_C denotes MACE at 1 year in QFR-guided PCI group and angiography-guided PCI group, respectively.

Assume a 1-year rate of MACE in standard angiography-guided PCI group of 8.7% according to multicenter, entire population clinical trials such as PANDA III trial (Comparison of BuMA eG Based BioDegradable Polymer Stent With EXCEL Biodegradable Polymer Sirolimus-eluting Stent in “Real-World” Practice) [14] and I-

LOVE-IT 2 trial (Evaluate Safety and Effectiveness of the Tivoli DES and the Firebird 2 DES for Treatment of Coronary) [15]. And assume a 1-year risk of MACE in QFR-guided PCI group of 6.1% according to clinical trials such as DEFINE-FLAIR trial (Functional Lesion Assessment of Intermediate Stenosis to Guide Revascularization) [9] and iFR-SWEDEHEART trial (Instantaneous Wave-free Ratio versus Fractional Flow Reserve in Patients with Stable Angina Pectoris or Acute Coronary Syndrome) [8]. Using a 2.5% level of one-sided significance and a maximum possible loss to follow-up rate of 5%, 3,830 participants (1,915 in each group) will provide 85% power to demonstrate that the QFR-guided strategy is superior to the angiography-guided strategy for the primary endpoint.

For the major secondary endpoint, the rate of MACE excluding periprocedural MI (ie, all-cause death, spontaneous MI, and any ischemia-driven revascularization) was 6.0% based on PANDA III [14] and I-LOVE-IT 2 trials [15]. Assuming an incidence in the QFR-guided arm of 4.0% (33% relative risk reduction), using a 2.5% level of 1-sided significance and a maximal loss to follow-up rate of 2.5%, a total of 3,830 patients will provide 80% power to demonstrate superiority.

12.3 Number of Enrollments for Each Site

This trial will be carried out simultaneously in a number of clinical institutions. In principle, the number of enrollments in each center will be distributed as evenly as possible to ensure adequate central representation. However, considering the feasibility and progress of the selection, the number of enrollments will be adjusted according to the actual situation, and the size of each center will be ensured to be relatively balanced. The final enrollment size of a particular center should not exceed 50% of the total number of enrollments.

12.4 Dropout Rate

The maximum dropout rate during the follow-up period is expected to be 5%, which includes all cases that are ultimately not included in the primary analysis, usually including serious violation of the trial protocol (affecting the main efficacy evaluation) adjudicated by the primary investigator, incomplete follow-up and missing of the time window of follow-up.

12.5 Analysis Population

The statistical analysis will be carried out on the basis of the following analysis population. Analysis population is clearly defined before starting the analysis. The analysis population includes:

Intention-to-treat (ITT): The intention-to-treat population consists of patients undergoing randomization but excluding those with randomization error or informed consent withdrawal prior to index PCI.

Per-protocol Set (PPS): The per-protocol population consists of patients who were successfully randomized and underwent the treatment strategy excluding those with major protocol deviations (including key inclusion/exclusion criteria violation, any intended vessels not treated in the control group, treatment not performed based on QFR measurements, the QFR measurement not performed per protocol in the test group, etc). Major protocol deviations will be finally adjudicated by steering committee and statistician by discussion.

As Treated Set (ATS): The as treated population consists of patients who are analyzed according to the actual strategy (angiography or QFR guidance) used for treatment rather than their randomization assignment.

Primary and major secondary endpoints will be analyzed in ITT, PPS and ATS (if applicable). The principal analyses of the primary and secondary endpoints will be performed by ITT.

12.6 Indicators and Methods of the Statistical Analysis

12.6.1 Demographic and Baseline Characteristics

Demographic information (age, gender, and nation), medical history, vital signs, preoperative examination, angiographic and procedural characteristics and other baseline data will be analyzed based on ITT.

Categorical variables will be summarized with counts and percentages from each category. Continuous variables will be summarized using the number of observations, number of missing data, mean, standard deviation (SD), median,

minimum, maximum, the 25th and the 75th percentiles, as well as confidence intervals (CIs) or confidence limits where applicable. Based on the descriptive analysis, categorical data will be compared between the two groups using likelihood ratio chi-square test, and when more than 25% of the cells of the contingency table have a frequency less than 5, then Fisher's exact test will be used. Continuous variables with normal distribution will be tested using two sample t-test, and non-normal continuous data will be tested with Wilcoxon Rank Sum test.

12.6.2 Lesion and Treatment Information

The descriptive analyses of lesion and corresponding treatment information will be performed on ITT. The total number of treated lesions and stents will be analyzed based on patient level. The detailed lesion information (length of lesion, reference vessel diameter etc.) will be analyzed based on lesion level. The detailed stent information (length of stent, stent diameter, etc.) will be analyzed based on stent level. The statistical analysis method is referred to 12.6.1.

12.6.3 Primary Endpoint

The analysis of MACE at 1 year will be performed on ITT, PPS and ATS (if applicable), and the principal analysis will be performed on ITT. The event count and percentage of MACE at 1 year will be provided. The analyses based on Kaplan-Meier survival analysis and Cox proportional hazards model will be provided as the primary analysis. The cumulative incidence curves for each group will be provided using Kaplan-Meier estimates and compared by the log-rank test. Estimates of the hazard ratios (HRs) and 2-sided 95% CIs will be calculated using a Cox proportional hazards model. Meanwhile, Cochran-Mantel-Haenszel $\chi 2$ test with center effect adjustment will be used to calculate event rate differences and corresponding 2-sided 95% CI between test and control group as the sensitivity analysis. The analyses based on multivariate Cox proportional hazards model with center, diabetes mellitus, single branch, single lesion, stenosis degree of more than 90% and TIMI blood flow <3 adjustment will also be as the sensitivity analysis. If the upper limit of the 2-sided 95% CI of the hazard ratio (HR) in MACE at 1 year

between QFR-guided and angiography-guided group is below 1, then the superiority conclusion will be confirmed.

In addition, if the actual results show that other baseline variables (such as age, gender, etc.) are significantly different between the test group and the control group, the statistician will fully communicate with investigator during data analysis stage. The true confounding factors will be further clarified by the univariate analysis result of baseline variables and primary endpoint, previous published literature and combined with the experience of clinical experts. These indicators will be considered to be included in the model for adjustment as a sensitivity analysis for the analysis of primary endpoint.

12.6.4 Secondary Endpoints

1) Major Secondary Endpoint: the analysis will also be performed on ITT, PPS and ATS (if applicable), and the principal analysis will be performed on ITT. The event count and percentage of MACE at 1 year, event rate differences and corresponding 95% CIs between test and control group will also be provided. The analysis strategy of major secondary endpoint will be the same as primary endpoint.

2) Other Secondary Endpoints: the descriptive analysis results will be provided, and the statistical analysis method is referred to 12.6.1. For the time-to-event outcomes, the cumulative incidence curves for each group will be provided using Kaplan-Meier estimates and compared by the log-rank test. Estimates of the HRs and 95% CIs will be calculated using a Cox proportional hazards model.

12.7 Missing, Unused or Erroneous Data

Missing data will not be imputed or replaced. Missing event data for the primary and secondary endpoints will be “censored” at the time of last follow-up in Kaplan-Meier survival analyses and Cox proportional hazards model analyses.

Erroneous or spurious data will be cleaned before performing statistical analysis. The data of withdrawal subjects will also be included in the final statistical

analysis. Reasons for withdrawal will be specified in the statistical report. The missing of primary endpoint data caused by early withdrawal will be handled with the methods for missing data as mentioned above.

Screening and acceptance testing of these data will be carried out in accordance with Data Management Plan. To this end, all data involved in the determination of endpoints will be screened for missing and unusual values. Any missing data that affect the ability to determine or analyze any endpoint will be queried by Data Management for confirmation of irretrievability. Unusual values, such as outliers, will also be queried, and if confirmed, will be used as recorded.

12.8 Statistic Analysis Plan

Statistical analysis is performed according to the provisions in ICH E9 and the relevant requirements in the guidelines for biostatistics of clinical trials promulgated by the China Food and Drug Administration (CFDA). And statistical analysis processes will be performed strictly following the Standard Operating Procedures (SOP) approved by the Medical Statistics Department of the National Center for Cardiovascular Diseases. All statistical analysis is implemented with SAS® version 9.4 (SAS Institute, Inc.). For details, please refer to the relevant documents.

The statistical analysis plan should be confirmed by the primary investigator and finalized before the data is locked. Prior to finalization, the initial statistical analysis plan may be modified considering actual conditions during the trial. In principle, major analysis principles, methods and analysis sets are not encouraged to be substantially modified, and all modifications will be recorded.

13. Prespecified Subgroups

The study includes a subgroup analysis for primary endpoint and major secondary endpoint. The prespecified subgroups including:

1) Age: ≥65 vs. <65

2) Gender: Male vs. Female

3) Diabetes mellitus: Yes vs. No

4) Acute coronary syndrome: Yes vs. Non-ACS

5) Smoking history: current vs. former vs. never

6) Body mass index (BMI): ≥30 Kg/m^2 vs. <30 Kg/m^2

7) Left ventricular ejective fraction (LVEF): >45% vs. ≤45%

8) Lesion location: Left main/ proximal anterior descending artery vs. others

9) Lesion length: ≥20 mm vs. <20 mm

10) Reference vessel diameter: ≥2.5 mm vs. < 2.5 mm by QCA

11) Stenosis severity: DS ≥70% vs. <70% by QCA

12) Multivessel disease: Yes vs. No

13) Calcified lesion: Severe calcification vs. non-severe calcification

14) Bifurcation lesion: Yes vs. No

15) Tandem lesion: Yes vs. No

16) Bending/tortuous lesion: Yes vs. No

17) QFR gray zone: QFR < 0.75 vs. = 0.75-0.85 vs. > 0.85 by ACL analysis

18) QFR based functional SYNTAX score (FSS_{QFR}): low tertile vs. intermediate tertile vs. high tertile by ACL

19) Residual QFR (rQFR): ≥ cutoff value vs. < cutoff value by ACL

20) QFR based residual functional SYNTAX score ($rFSS_{QFR}$): $rFSS_{QFR}$ = 0 vs. ≥1 by ACL

21) Center experience for invasive physiology: experienced centers vs. less experienced centers, experienced center defined as the number of FFR/ iFR > 100 per year

22) Learning experience with QFR: first-half enrolled cases in each center vs.

second-half enrolled cases in each center

14. Data Management

14.1 Fill-in of eCRF

eCRF via Electronic Data Collection (EDC) system is used in this trial. The eCRF should be filled in by investigators and completed for each enrolled patient. The completed eCRF should be checked by the monitors.

14.2 Data Entry and Modification

For questions or doubts concerning data in eCRF, data administrator will try to clarify them by delivering a question form to investigator, and the investigator is responsible for resolving the question. Data administrator will confirm with the answer provided by the investigator and may issue the question form again if necessary.

14.3 Database Lock-up

After confirmation of the established database and data review, the data management personnel, primary investigators, statistical analysts, sponsor personnel, and monitor management should review the data and complete the final definition and judgment of the analysis population; then the database is locked by the data administrator. After the database is locked, it is submitted to statistical analysis.

15. Trial Termination or Withdrawal

CFDA retain the right to terminate the study. Besides this, in each of the following situation, it is up to the sponsors and the primary investigators to jointly decide whether the trial should be terminated or not.

- Adverse events might result in unanticipated, significant, or unacceptable risk to subjects selected for the study.
- Clinical trial institutions and investigators do not comply with relevant laws, standard for clinical trial quality management, or clinical trial protocol, and the violation is still serious or persistent after promptly indication and correction.
- The risk has exceeded the possible benefits, or there are sufficient data to determine the safety and efficacy of the treatment strategy for testing, the trial should be suspended or terminated.
- If the CEC finds that the rights of subjects cannot be guaranteed after follow-up supervision of clinical trial institutions, they might provide written request to suspend or terminate the clinical trial.

In any of the above cases, the investigators shall notify clinical trial institutions of each site within 5 days with a written explanation. Subjects who have been enrolled will continue to enjoy the rights they have in accordance with the trial protocol until the trial is terminated.

16. Quality Control of the Trial

Investigators participating in this clinical trial should complete Good Clinical Practice (GCP) training, be qualified, and experienced in multicenter clinical trials. Participating personnel should maintain relatively stable. All research personnel participating the FAVOR III China study will also receive the SOP training for the clinical trial. Operators performing PCI are supposed to have sufficient relevant clinical experience.

All centers should be equipped with online QFR setup with capacity to transfer images during angiography, together with documented experience and qualified expertise in QFR analysis and the capacity to follow the study protocol and timely complete scheduled tasks.

Monitors will visit all trial centers regularly according to the monitoring plan to verify compliance with the protocol and regulatory requirements, check the original

data and assist the investigators to conduct the trial. The sponsor, the regulatory authorities and their agents may conduct independent inspections of the sites at any time during or after the research period to determine whether it is in accordance with the protocol and SOP, to perform the research-related activities, records, analysis, and accurately reporting the data.

17. Ethical Consideration and Informed Consent

17.1 Ethical Consideration

This clinical study was designed and shall be implemented and reported in accordance with the international conference on harmonization (ICH) Harmonized Tripartite Guidelines for Good Clinical Practice, with applicable local regulations. The study is conducted in accordance with the ethical principles laid down in the Declaration of Helsinki (2013).

The potential risks of this study come from adverse events caused by coronary angiography and PCI, including but not limited to vascular injury at the puncture site, contrast agent allergy, renal damage, arrhythmia, myocardium Infarction, death, etc. However, the potential risks of this study do not come from QFR measurement.

Participating in this study will not increase the cost of diagnosis and treatment. The cost of coronary angiography, possible interventional treatment (and its potential complications) is not free of charge. This study will enhance the understanding of the guidance value of QFR for PCI, which may help the operators choosing more appropriate treatment strategy in future practice. After signing this informed consent form, the subjects will not lose any legal rights and interests. The sponsor has provided the insurance for all subjects participating in this clinical trial in accordance with Chinese laws and regulations.

17.2 Informed Consent Process

Investigators must ensure that participants are clearly and fully informed about the purpose, potential risks, and other critical issues regarding clinical studies in

which they volunteer to participate. The participants are notified of their voluntary participation and of their right to withdraw from the study at any time. The participants are also informed that withdrawal from the study does not influence their future treatment. Eligible participants will be provided with sufficient time for consideration after initial oral information and are enrolled only when they fully understand the trial and sign the informed consent form. Freely given written informed consent must be obtained from every participant or, in those situations where consent cannot be given by participants, their legally acceptable representative, prior to clinical study participation. The rights, safety, and well-being of the study participants are the most important considerations and should prevail over interests of science and society. If there is any question that the prospective participant will not reliably comply with study procedures and/or follow-up, they should not be entered in the study.

Informed consent form is signed by investigators and patients or their guardians in duplicate and each party should keep a copy of the informed consent with signature of both parties.

17.3 Responsibilities of the Investigator and IRB/EC

The protocol and the proposed informed consent form will be reviewed and approved by a properly constituted Institutional Review Board / Ethics Committee (IRB/EC) at each site. A signed and dated statement that the protocol and informed consent have been approved by the IRB/EC is required before site initiation. Prior to study start, the site PI is required to sign a protocol signature page confirming his/her agreement to conduct the study in accordance with these documents and all of the instructions and procedures found in this protocol and to give access to all relevant data and records to monitors, auditors, IRBs/ECs, and regulatory authorities as required. Investigators must agree to apply due diligence to avoid protocol deviations.

18. Regulations for Adverse Events

18.1 Adverse Events

Adverse events (AE) refer to unfavorable medical events that occur during clinical trials, including but not limited to discomfort, signs or laboratory abnormalities. Abnormal laboratory results that are obviously unrelated is not considered AEs, except in the following cases:

- PI believes that laboratory abnormalities have clinical significance,
- Medical intervention must be taken due to laboratory abnormalities, or
- Subject must withdraw from the trial due to laboratory abnormalities.

18.2 Serious Adverse Events

Serious adverse events (SAE) refer to deaths or serious deterioration of physical conditions of patients during clinical trials, including fatal diseases or injuries, permanent defects in body structure or body functions, hospitalization or extended hospital stay, medical or surgical intervention required to avoid permanent defects in body structure or body functions and events causing fetal distress, fetal death, congenital anomalies or congenital defects, etc.

18.3 Severity of Adverse Events

For each adverse event, severity or changes in severity should be recorded in each follow-up. The following definitions of severity can be applied:

Mild	The adverse event is learned by asking the patient.
Moderate	The adverse event is learned by the active narration by the patient and is endurable to the patient.
Severe	The adverse event is learned by objective symptoms and is unendurable to the patient.

*Pay attention to the difference between severe adverse events and serious adverse events. Please refer to the definition of serious adverse events.

Adverse events with a severe severity should be periodically collected and summarized, and should be reported to the investigators, the sponsor and clinical trial institutions.

18.4 Records of Adverse Events

During the clinical trial, the investigator should record all directly observed and self-reported adverse events on the case report form. Investigators should use standard medical terms instead of colloquial expression, including at least the items listed below:

- Start and end dates
- Severity
- Causal relationship
- Measures
- Outcomes of adverse events

All adverse events that occur before the end of operation (withdrawal from the catheterization laboratory) should be collected and recorded. The events associated with this study are adverse events occurred during coronary angiography or PCI. If follow-up interventions are required, detailed records of the (serious) adverse events that occurred during the procedure are also required.

18.5 Adverse Events Reporting

In case of serious adverse events, the investigator should immediately take appropriate treatment measures for the subject and send written report to the sponsors, primary investigators, ECs, or local clinical trial institution within 24 hours. For death events, investigators should provide the EC and the sponsor with all the information required. The sponsor shall report to the corresponding CFDA within 5 working days after being informed. The sponsor shall also inform other clinical

institutions and investigators participating in the trial and notify the corresponding Ethics Committee in a timely manner.

19. Protocol Amendments

Researchers should strictly follow the trial protocol, and should not deviate from the protocol or substantially change the protocol without the approval of the sponsor and EC. In case of emergency, such as the subject facing immediate danger, it can also be reported in written form afterwards. The EC should strictly consider whether deviations from the protocol may affect the rights, safety, and health of the subjects, or the scientificity and integrity of the trial. After the protocol approved by the EC, any major change during the study must be reported and get approval from the EC before implementation.

20. Direct access to source data and files

Source data refers to the original records of clinical findings, observations and other activities in clinical trials and all the information in their approved copies, which can be used for clinical trial reconstruction and evaluation.

Source documents refer to printed documents, visual documents or electronic documents that contain source data.

The researcher/institution will allow relevant personnel to directly access the source data/documents in order to cooperate with the inspections, inspections, EC evaluations and inspections of the regulatory authorities related to the trial. The signing of the informed consent by the subject means that the sponsor or designated person is allowed to view and copy the information related to participating in the trial in the medical record (the researcher should ensure that the subject's identity information will be deleted from the copy of the medical record). As part of the informed consent form, the investigator will ask the subject to allow the trial monitor or regulatory agency to review any records that identify the subject in this clinical trial at the research center, but it must be kept confidential. This information can be shared with regulatory agencies. However, the sponsor should not disclose the

subject’s personal and private information in other ways in accordance with local data protection laws.

21. Insurance

If subjects suffer from any trial-related harm, the sponsor will provide an insurance to give an appropriate compensation to the subjects or their beneficiaries, based on the severity and duration of the injury. When the harm is caused intentionally or due to a major negligence of the site, the sponsor will consult with the sites according to the investigator’s agreement and study contract.

22. Confidentiality Principle

The medical information of each subject obtained in this study is confidential and is prohibited from being disclosed to third parties. Such information can only be provided to the subject’s doctor or other relevant medical personnel responsible for the subject’s health after the subject’s consent is obtained.

23. Publication Policy

Primary and secondary reports of study findings will be published in peer-reviewed journals. Proposals for any presentation and publication incorporating data obtained from subjects involved in the FAVOR III China trial must be submitted for review and approval by the publications committee. The primary publication will be authored by the trial’s writing committee. No site is permitted to present or publish data obtained during the conduct of this trial without prior approval from the publications committee. Until the results of primary outcome published and/or reported, any site is not allowed to publish and/or report its results. Authorship for FAVOR III China trial-related publications will be determined by the publications committee considering the contribution to the trial and the relevant analyses.

24. Responsibilities

The study Steering Committee, Study Chair, and Co-Chair maintain responsibility for the overall conduct of the study, including preparing and submitting study protocol, sites management and monitoring, data analysis and reporting. The

International Advisory Board will be responsible for the study design, protocol preparing, and data reporting. The Technological Cooperative Organization is responsible for training and qualification of QFR measurements. The Health Economics Center is responsible for the conduct of the quality of life and the economics and cost effectiveness portions of this study. CEC will review and adjudicate all primary endpoint events, major secondary endpoint, and other selected secondary endpoints in a blinded fashion based on study definitions. The DSMB is responsible for recommending the Steering Committee to modify or stop the trial if there are any safety issue. The ACL will characterize coronary anatomy for participants undergoing coronary angiography and procedural outcomes for those undergoing PCI, provide off-line QCA analysis and the QFR calculation for both groups. The Data Management Center is responsible for the treatment allocations of eligible participants, receipt and processing of data collected by the clinical sites, ACL and coordinating centers. Statistical Analysis Center is responsible for statistical analysis and reporting.

25. Funding Source

The FAVOR III China trial is an investigator-initiated study, without any external sources of finance.

References

1. Bech GJ, De Bruyne B, Pijls NH, et al. Fractional flow reserve to determine the appropriateness of angioplasty in moderate coronary stenosis: a randomized trial. Circulation 2001;103(24):2928-34. DOI: 10.1161/01.cir.103.24.2928.

2. Tonino PA, De Bruyne B, Pijls NH, et al. Fractional flow reserve versus angiography for guiding percutaneous coronary intervention. N Engl J Med 2009;360(3):213-24. DOI: 10.1056/NEJMoa0807611.

3. De Bruyne B, Pijls NH, Kalesan B, et al. Fractional flow reserve-guided PCI versus medical therapy in stable coronary disease. N Engl J Med 2012;367(11):991-1001. DOI: 10.1056/NEJMoa1205361.

4. Neumann FJ, Sousa-Uva M, Ahlsson A, et al. 2018 ESC/EACTS Guidelines on myocardial revascularization. European heart journal 2018. DOI: 10.1093/eurheartj/ehy394.

5. Al-Lamee R, Howard JP, Shun-Shin MJ, et al. Fractional Flow Reserve and Instantaneous Wave-Free Ratio as Predictors of the Placebo-Controlled Response to Percutaneous Coronary Intervention in Stable Single-Vessel Coronary Artery Disease. Circulation 2018;138(17):1780-1792. DOI: 10.1161/CIRCULATIONAHA.118.033801.

6. Xaplanteris P, Fournier S, Pijls NHJ, et al. Five-Year Outcomes with PCI Guided by Fractional Flow Reserve. N Engl J Med 2018;379(3):250-259. DOI: 10.1056/NEJMoa1803538.

7. Volz S, Dworeck C, Redfors B, et al. Survival of Patients With Angina Pectoris Undergoing Percutaneous Coronary Intervention With Intracoronary Pressure Wire Guidance. Journal of the American College of Cardiology 2020;75(22):2785-2799. DOI: 10.1016/j.jacc.2020.04.018.

8. Gotberg M, Christiansen EH, Gudmundsdottir IJ, et al. Instantaneous Wave-free Ratio versus Fractional Flow Reserve to Guide PCI. N Engl J Med 2017;376(19):1813-1823. DOI: 10.1056/NEJMoa1616540.

9. Davies JE, Sen S, Dehbi HM, et al. Use of the Instantaneous Wave-free Ratio or Fractional Flow Reserve in PCI. N Engl J Med 2017;376(19):1824-1834. DOI: 10.1056/NEJMoa1700445.

10. Mallet ML. Proarrhythmic effects of adenosine: a review of the literature. Emerg Med J 2004;21(4):408-10. DOI: 10.1136/emj.2004.016048.

11. Tu S, Westra J, Yang J, et al. Diagnostic Accuracy of Fast Computational Approaches to Derive Fractional Flow Reserve From Diagnostic Coronary Angiography: The International Multicenter FAVOR Pilot Study. JACC Cardiovascular interventions 2016;9(19):2024-2035. DOI: 10.1016/j.jcin.2016.07.013.

12. Xu B, Tu S, Qiao S, et al. Diagnostic Accuracy of Angiography-Based Quantitative Flow Ratio Measurements for Online Assessment of Coronary Stenosis. Journal of the American College of Cardiology 2017;70(25):3077-3087. DOI: 10.1016/j.jacc.2017.10.035.

13. Garcia-Garcia HM, McFadden EP, Farb A, et al. Standardized End Point Definitions for Coronary Intervention Trials: The Academic Research Consortium-2 Consensus Document. European heart journal 2018;39(23):2192-2207. DOI: 10.1093/eurheartj/ehy223.

14. Xu B, Gao R, Yang Y, et al. Biodegradable Polymer-Based Sirolimus-Eluting Stents With Differing Elution and Absorption Kinetics: The PANDA III Trial. Journal of the American College of Cardiology 2016;67(19):2249-58. DOI: 10.1016/j.jacc.2016.03.475.

15. Han Y, Xu B, Jing Q, et al. A randomized comparison of novel biodegradable polymer- and durable polymer-coated cobalt-chromium sirolimus-eluting stents. JACC Cardiovascular interventions 2014;7(12):1352-60. DOI: 10.1016/j.jcin.2014.09.001.

Appendix 1. QFR Standard Operating Procedure

QFR Standard Operating Procedure

Step-by-step manual

The AngioPlus system (Pulse Medical Imaging Technology, Shanghai Co., Ltd., Shanghai, China) is used for computation of QFR in this trial. The procedure of QFR analysis is described step-by-step in below.

1. Coronary angiography acquisition

In order to obtain accurate QFR measurement, analysts are required to follow the SOP for angiographic image acquisition as follows:

1) Identify the interrogated vessel by (previously acquired) coronary angiography or other imaging modalities, e.g., coronary computed tomography angiography (coronary CTA).
2) Choose angiographic imaging catheter or guiding catheter (minimum size 5F, preferably ≥6F) and ensure a co-axiality of catheter with respect to the vessel.
3) Inject nitroglycerin before the acquisition to eliminate arterial spasm.
4) Set angiographic imaging frame rate higher than or equal to 15 frame/sec.
5) Acquire two angiographic image runs according to the recommended angiographic projections and ensure that two angiographic image runs are acquired at different angles ≥25º.
6) Start recording angiographic images at least 1 second prior to contrast injection.
7) Inject the contrast at constant speed of 4 ml/sec until the distal vessel is filled.

Besides, a few details below need to be noted:

1) Acquire angiographic images using the recommended angiographic projections (Table 1). Adjust angiographic projections 5-10 degree if the recommended projection has no clear presentation of the lesion segment.

Table 1. Recommended angiographic projections for QFR

**Interrogated Vessel**	**1st view**	**2nd view**
LM and LM bifurcation	RAO 20, CAU 45	AP, CAU 10
LAD and diagonal artery	AP, CRA 45	RAO 30, CRA 20
LCX and OM	LAO 10, CAU 25	RAO 25, CAU 35
RCA/PLA/PDA	LAO 45, CAU 10	LAO 20, CRA 20

2) Ensure that saline and nitroglycerin be completely washed out from the catheter, before the angiographic runs.

3) If the image is not sufficient for QFR analysis (Figure 1), additional angiography needs to be acquired to reach the requirement of QFR analysis.

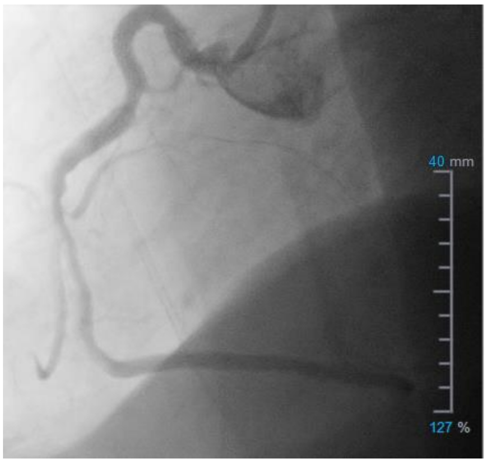

Figure 1. Suboptimal angiographic image quality due to unstable injection of contrast medium

4) Criteria of good quality of contrast injection: It can be clearly visualized in the angiographic image runs that the contrast medium enters the vessel via catheter and gradually fills the entire vessel until it reaches the distal segment.

5) Take the 2nd angiographic projection at least 15 seconds after acquiring the 1st one to ensure the contrast medium completely washed out.
6) Do not move bed during imaging acquisition.

2. Angiographic image transfer

The angiographic data are transferred to the AngioPlus system by local network of catheter laboratory. Depend on the various network configuration, the data is manually transferred directly from angiography system to AngioPlus through DICOM network, or the data is automatically transferred to local PACS system and then AngioPlus manually retrieve it. The data transfer is required to be completed in one minute in this trial.

3. Angiographic run selection

Optimal requirements for selecting appropriate angiographic image runs for analysis (Figure 2):

1) Clear images with good contrast filling;
2) The run has at least one frame with minimal overlap or foreshortening, especially at stenotic segment.

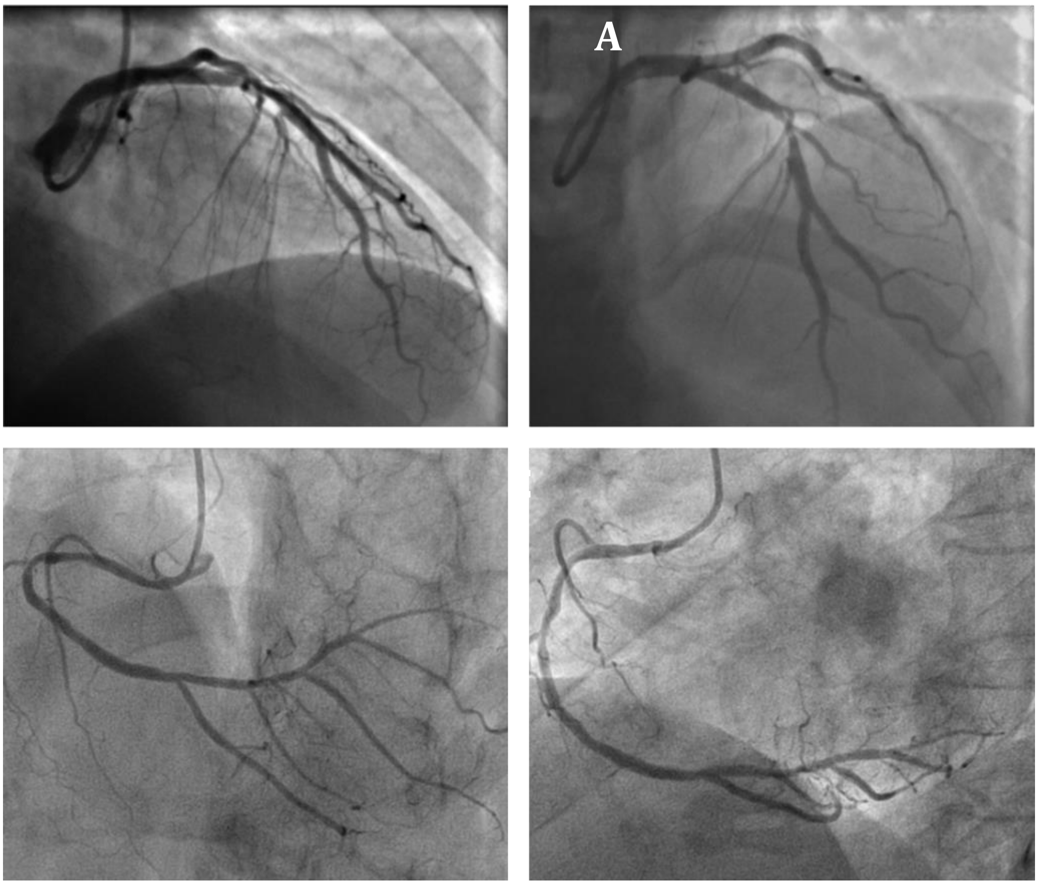

Figure 2. Preferably choose optimal angiography runs with less vascular overlaps and shortening, especially in lesion segment. Suboptimal angiography with vessel overlaps (A) and serious shortening (C). Optimal angiography with less vascular overlaps (B) and less shortening (D).

Choose Series Selection (Figure 3, red box) and load two optimal angiographic image runs. If the angulation between two projections is less than 25 degrees, the second image run is not allowed to be loaded by AnigoPlus software.

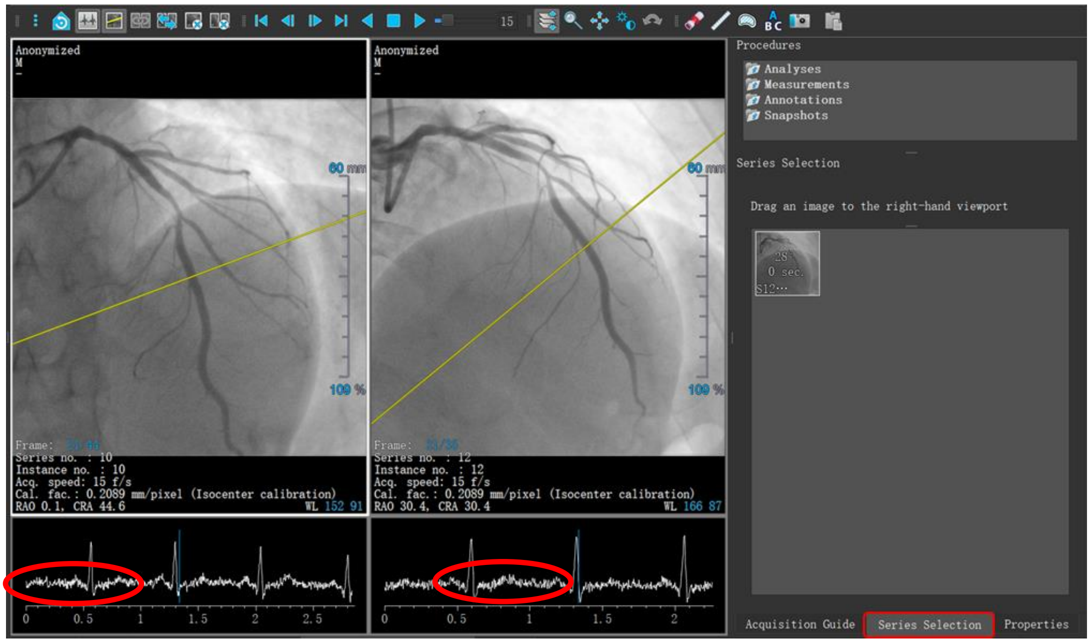

Figure 3. Select series (red box) and two angiographic image runs acquired at different angles ≥25° (red circle).

4. Frame selection

The frame selected for analysis must satisfy the following requirements:

1) The lesion(s) is clearly exposed without overlapping or foreshortening;
2) The contrast fill is good for the interrogated vessel;
3) The interrogated vessel should have no motion artifact;
4) Optimal to select the frame with sharp lumen contours.
5) The frame selected in the two projections should be in the same cardiac phase.

After selecting optimal frames for QFR analysis in both angiographic projections, select the Create single vessel analysis-button (**Error! Reference source not found.**, white arrow) in the top panel for 3D vessel reconstruction.

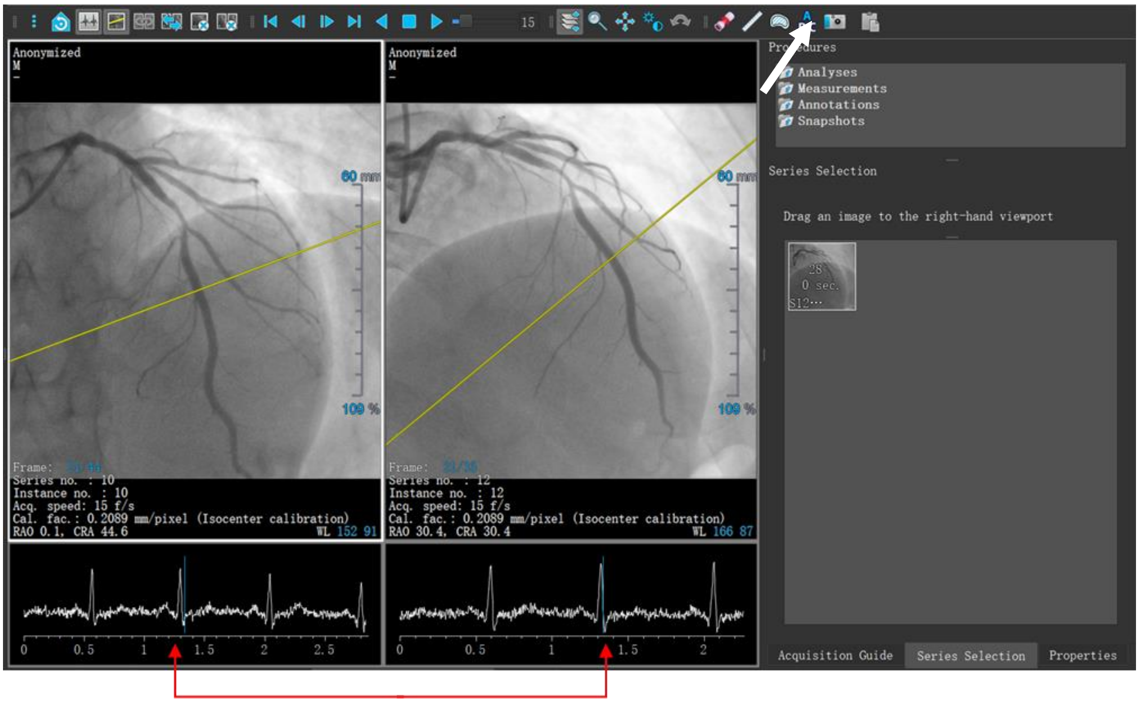

Figure 4. Frame selection and “Create single vessel analysis” activates the 3D reconstruction. Note that both runs are in the same end-diastolic phase

5. Offset correction

For a three-dimensional reconstruction of the target vessel, offset correction between two angiographic projections is achieved by selecting one corresponding landmark near the lesion in each selected frame (Figure 5). Recommended anatomical landmarks can be a bifurcation, a localized stenosis or the off-spring of a side branch.

There are a few preferences of landmark selection:

1) The bifurcation close to the lesion on the interrogated vessel.
2) Landmark on the segment with less vessel overlap.
3) Landmark with small angle between epipolar line and target vessel.

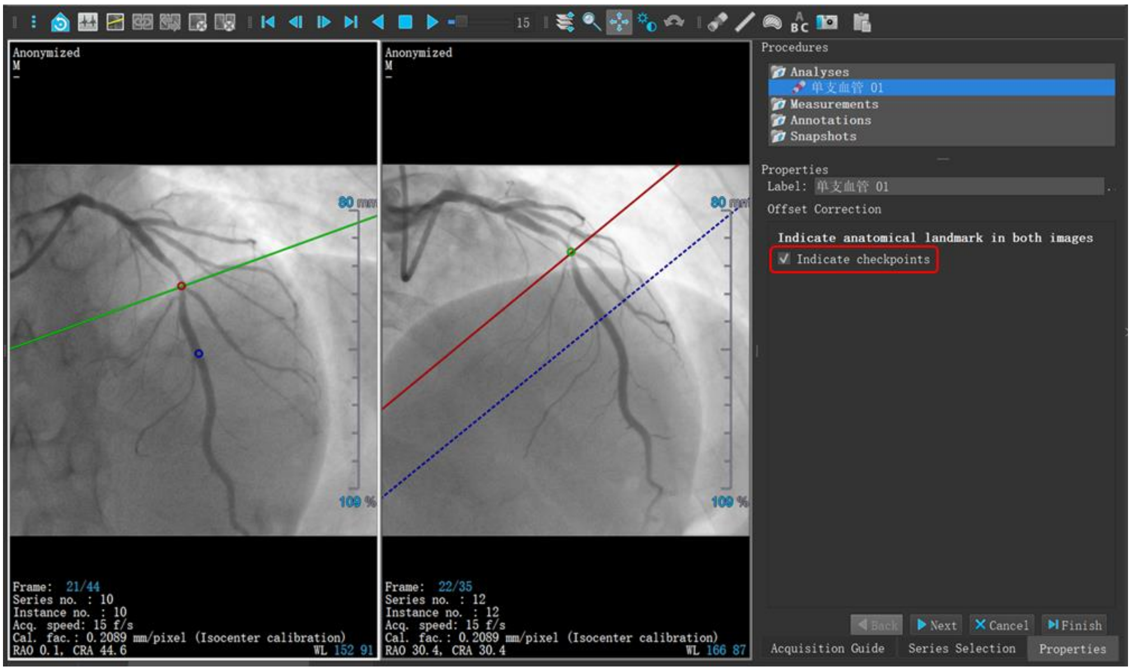

Figure 5. Corresponding point is marked by the red and green spot in the left and right panel, respectively. Users can use the Indicate checkpoints option, to check if the projections are registered properly or not.

After selecting corresponding landmarks, users can use the Indicate checkpoints option, to check if the projections are registered properly. The procedure is described as follows:

1) Tick off the Indicate checkpoints box (Figure , red box).
2) Place another identifiable corresponding landmark proximal or distal to the previously selected corresponding point in one projection, and then check if the blue epipolar line is crossing the correspondent anatomic landmark on the other projection.
3) Adjust the offset point if the checkpoints are not consistent in the two projections.

6. Defining interrogated vessel segment

Definition of the vessel region of interest for analysis should follow the principles in below:

1) The segment should contain all stenosed lesions and be no less than 20 mm.

2) The proximal (start) point should be placed in the healthy segment. If the proximal LAD is completely diseased (diffuse lesions), the start point should be placed at the left main coronary artery. And the start point should be placed at the ostial of LAD in other situations.
3) The distal (end) point is placed at the healthy segment distal to the lesion.
4) Ensure that the start and end points on both frames are anatomically corresponded to each other. If not, user need to manually adjust them.

After selecting both proximal and distal points, the vessel path line is automatically identified in both projections (Figure 6). If the path line deviates from the interrogated vessel, users need to drag it back into correct position using supporting points. User can use the lock pathlines function to lock the path line when path line position is accepted (Figure , red box).

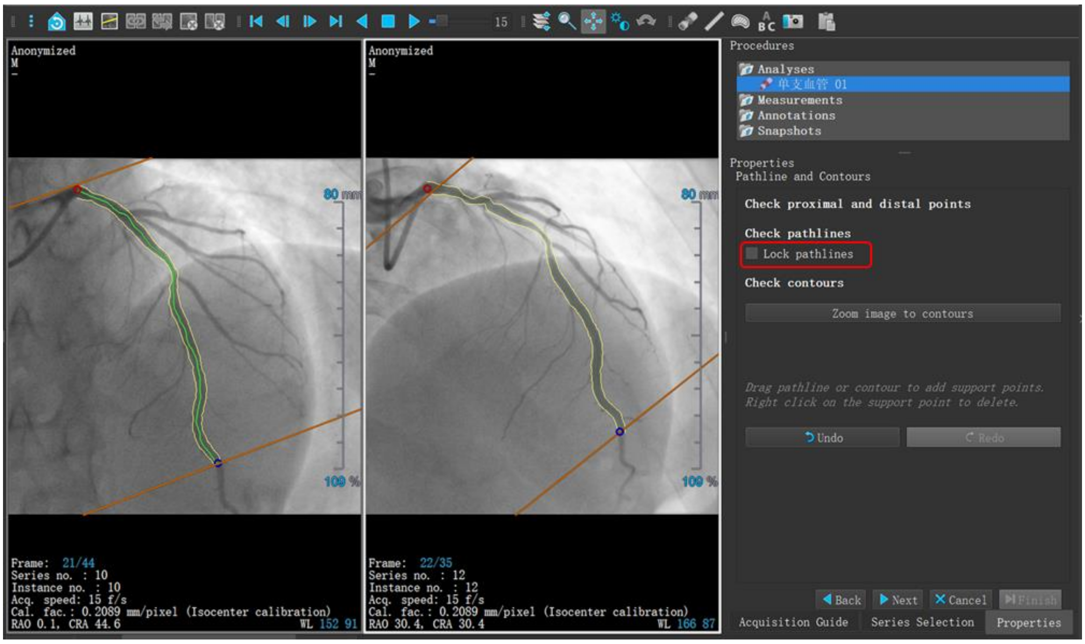

Figure 6. Defining interrogated vessel segment. The proximal point is marked by red circles while the distal point is marked by blue circles. The interrogated vessel path line is indicated by the green line. The path line is locked by ticking Lock path lines (red box)

7. Checking the lumen contour

The vessel lumen contour is automatically delineated in both projections (Figure). Click on Zoom image to contours button to automatically zoom the two views for

contour checking. When the detected lumen borders deviates from the true vessel lumen, it is necessary to manually adjust the contours by adding correction points at the true lumen borders. Right-click on the correction point enables point deletion.

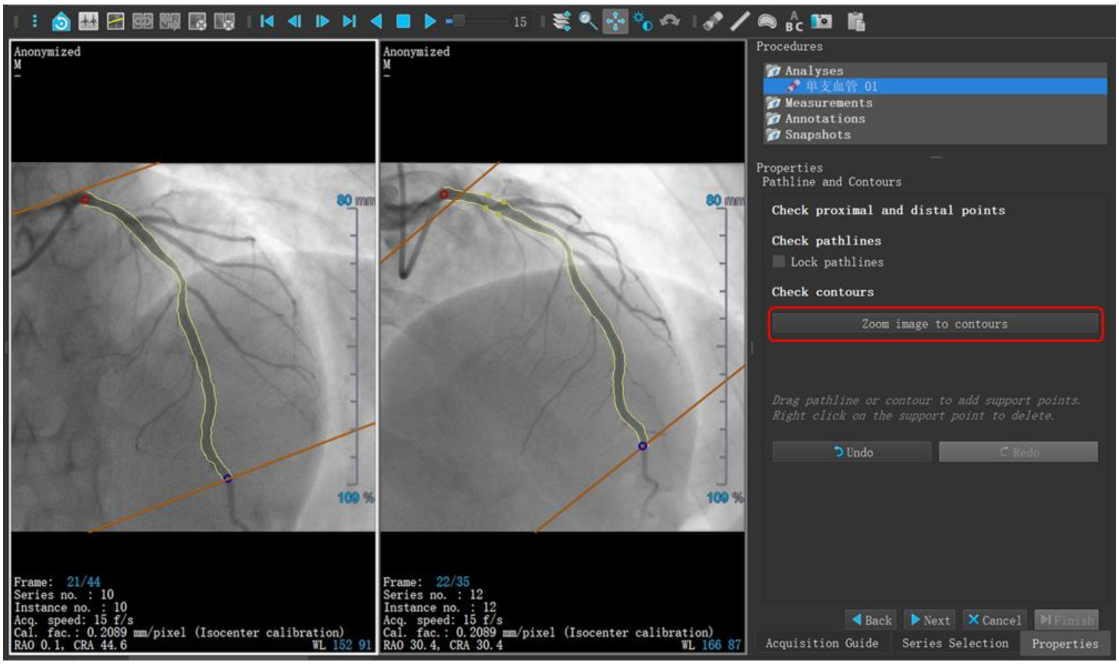

Figure 7. Check the segmented lumen contour. The yellow lines indicate the lumen contour and can be corrected by adding yellow points at the true lumen borders. For improved view, click “Zoom image to contours” (red box).

8. Defining corresponding points (optional)

In cases of suboptimal projection correspondence (Figure 8), user can manually define an additional corresponding point to improve calibration. Corresponding point is defined as follows:

1) Tick on the Corresponding points;
2) Select an identifiable landmark point in occlusion position with both projections;
3) Adjust the position of the markers until the curves of long and short diameter curves are best aligned.

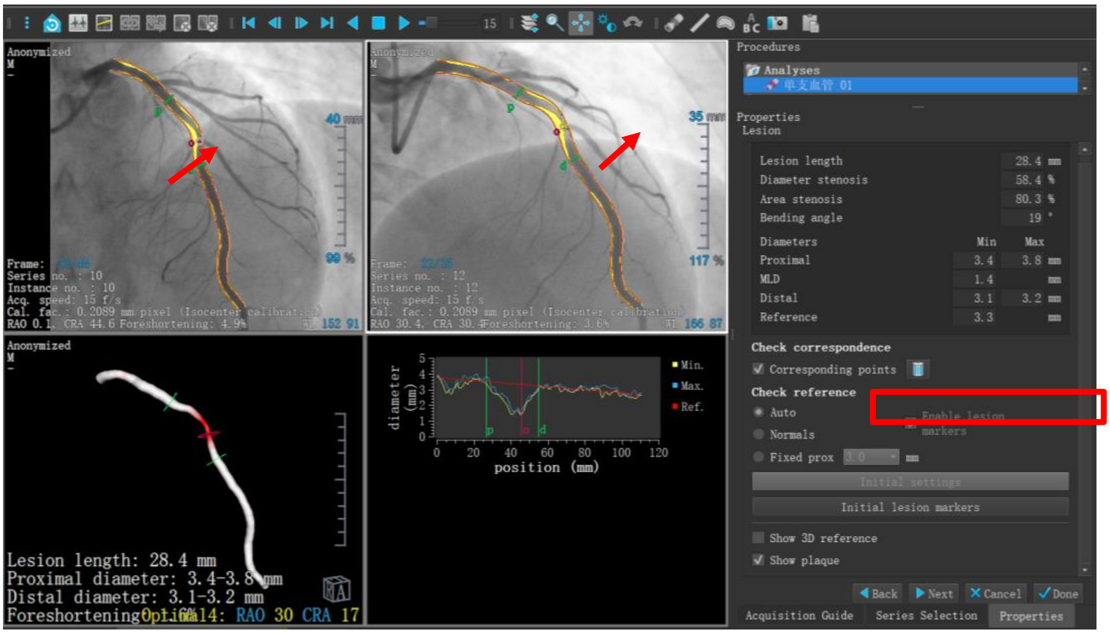

Figure 8. Define correspondence point. Tick on the Corresponding points (red box) and add an identifiable landmark point (red arrow) in occlusion position with both projections.

Diameter curve mismatch

If the diameter curves are not aligned (Figure), please check if former steps are not correct. Poor correspondence of the diameter curves may be caused by the following reasons:

1) Two frames are selected in different cardiac phases;
2) Wrong corresponding point defined at the step Offset correction;
3) Mismatch of start point or end point between the two projections;
4) Reversed start- and end-point in one of the projections;
5) Errors in lumen contouring.

It is noted that some eccentric lesions may cause difference between the short and long diameter curves.

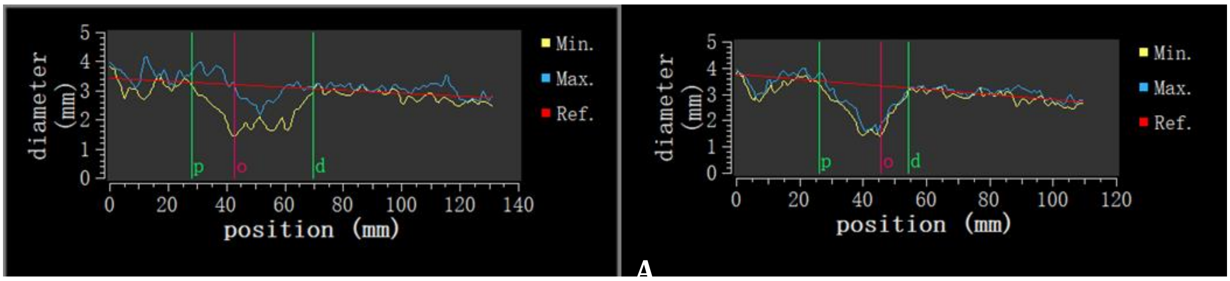

Figure 9. Diameter curve mismatch. A) Poor correspondence of diameter graphs. The stenosis segments are shifted sideways. B) Analysis improved after changes was made in correspondence of the two projections.

9. Reference vessel

After confirming the vessel contouring, the reference lumen (i.e. as if the vessel is healthy) is subsequently reconstructed. The “Plaque” is marked (in yellow) in the segments when actual lumen contour is narrower than its reference lumen (Figure).

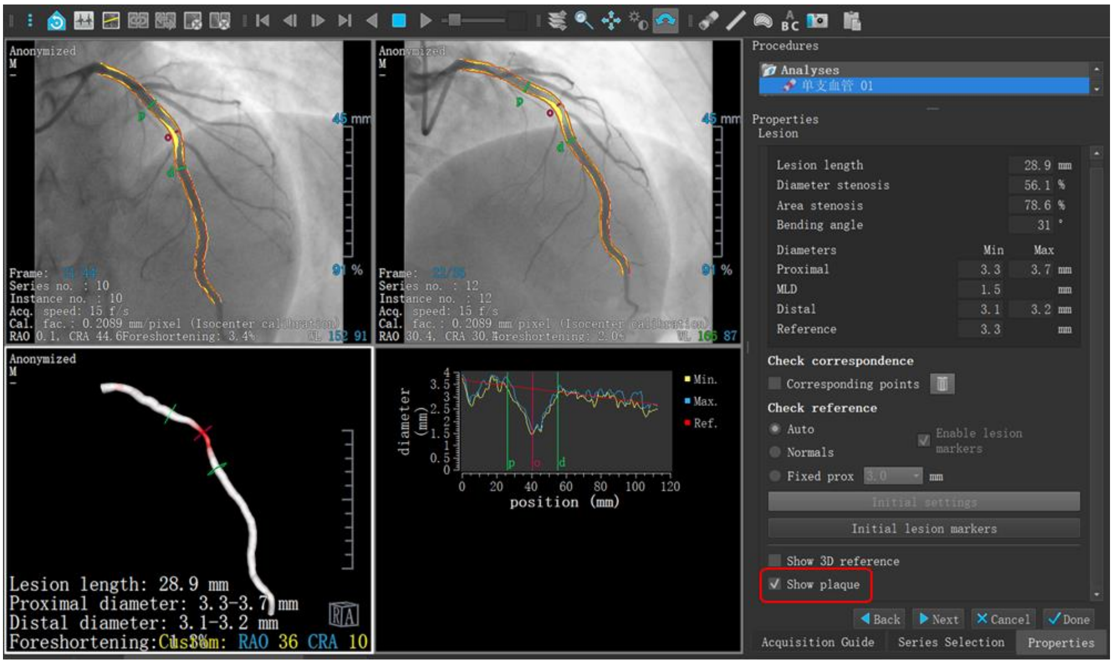

Figure 10. Reference lumen contour (red contours) and true lumen contour. Show/hide yellow plaque (red box)

The software offers three ways to define the reference vessel:

Auto

By default, the reference vessel is computed by this Auto method. Automatic reference vessel is accepted if

1) The reference vessel is tapered from the proximal to distal vessel segment;

2) The reference vessel delineates normal (healthy) segment;

3) The diameter of the reference vessel is smaller than that of segments of positive remodeling but larger than that of stenotic segments.

Pay special attention to diffusely diseased vessel where automatically generated reference vessel is much smaller than it is supposed to be.

If the automatic reference fails to fulfill the above requirements, users shall choose Normal parts or Fixed prox to adjust the reference lumen contour.

Normal parts

In choosing Normal parts, the user manually identifies two normal segments represented by green areas (Figure 1). The reference lumen is calculated by linear regression on the basis of the selected segments.

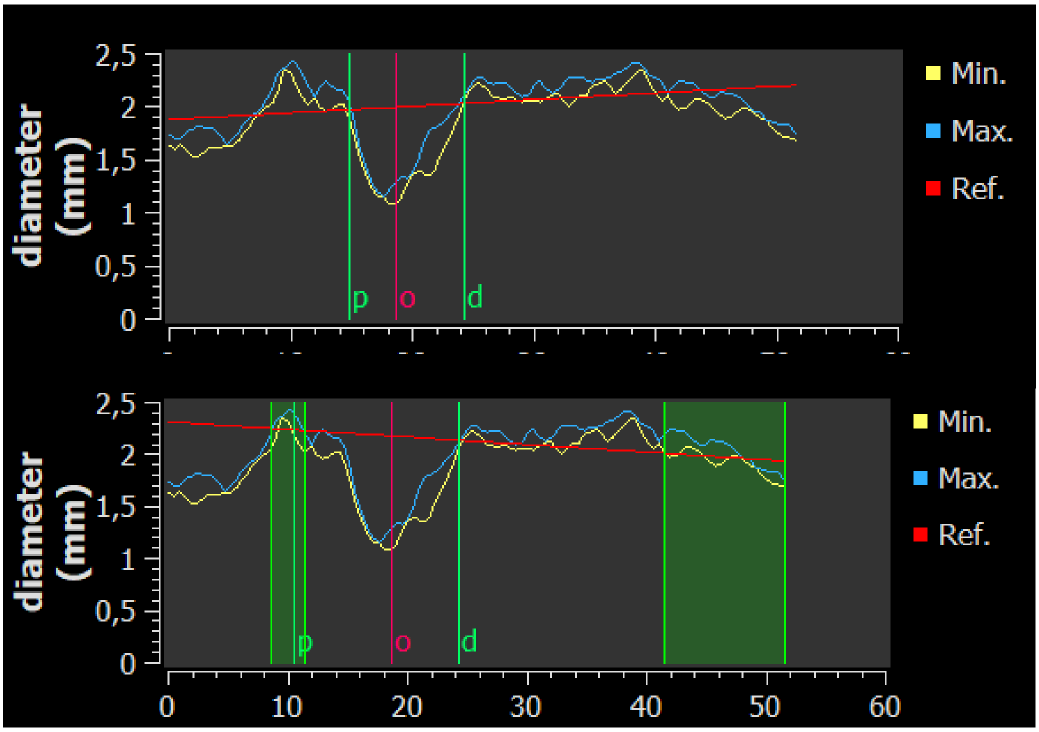

Figure 11. Illustrative case with adjusted reference diameter after using Normal parts as adjustment. Reference lumen contour changed from Automatic generated, non-tapering reference (Auto) (A) to a tapering reference lumen contour based on Normal parts (B).

Fixed prox

In diffusely diseased vessels with parts of segment exceeding the proximal reference size that cannot be corrected by Normal parts, Fixed proximal is recommended.

The Fixed prox enables user to specify the proximal reference lumen size at an identified location with a 0.25 mm interval in range of 2 to 5 mm. The slope of the reference lumen can be adjusted by selecting a normal distal segment (Figure 1).

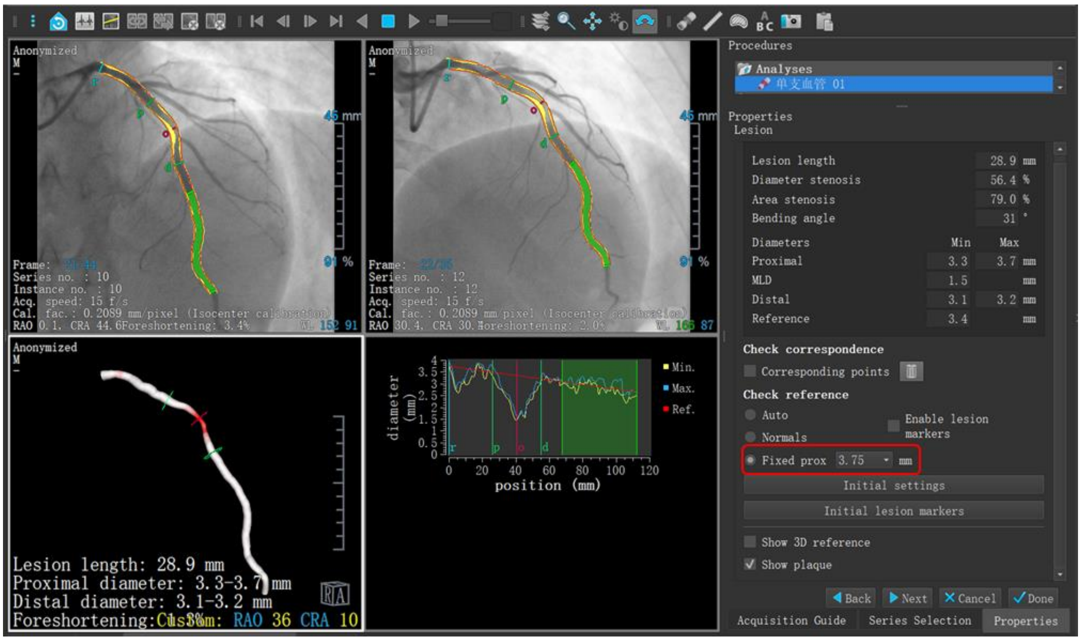

Figure 1. Reference lumen contour editing using the ”Fixed Prox” function (red box).

10. QFR computation

Press Next, after confirming the reference lumen contour. According to study protocol, nitroglycerin is required to be adminstered before both acqusitions. Therefore select Yes for the question ”Nitro adminstered before both injection” .

And then select the type of coronary artery, Left Main/LAD or Other coronary accordingly.

Fixed-flow QFR (fQFR) computation

fQFR (fixed-flow QFR) is automatically calculated (Figure 13, red box).

Frame count-based QFR (cQFR) computation

Frame count-based QFR (cQFR) requires additional step of frame count to compute patient’s specific flow velocity. Frame count (Figure , yellow box) is performed as follows:

1) Select one of the two projections for frame count with good contrast filling and a constant contrast flow/speed. It is not recommended to choose the projection where contrast flow velocity alter too dynamtically;
2) Specify patient state that whether the chosen projection is acquired at rest or during hyperaemia. According to FAVOR III study protocol, “Contrast” state is always used;
3) Choose the start frame in which the contrast arrives at the proximal path line point;
4) Choose the end frame in which the contrast arrives at the distal path line point;
5) If the proximal or distal point path line is reached by the contrast between two frames, the first of the two frames are selected, and the +0.5 checkbox is checked. Optionally user can also move the proximal or distal vessel delimiters (Figure 13, red arrow) forwards and backwards, so that the updated delimiters align with contrast fronts.

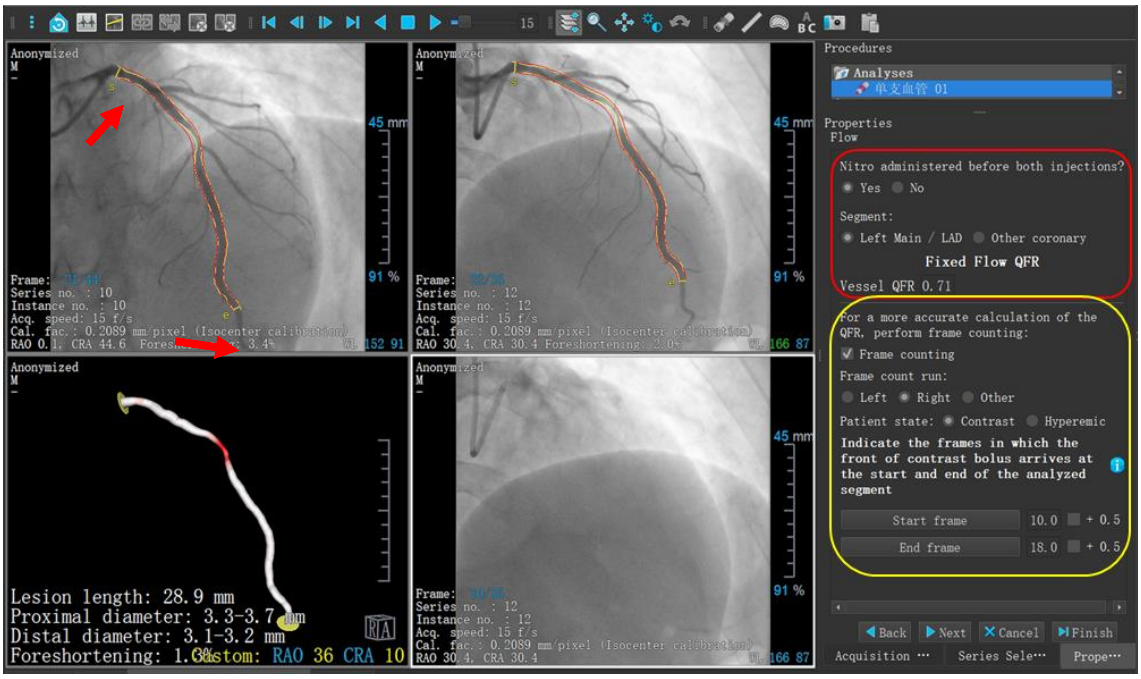

Figure 13. Vessel for fixed flow QFR computation (red box) and frame count QFR analysis (yellow box) by indicating the start- and end frame for contrast flow through the segmented vessel part.

The following QFR values are calculated and listed after completing frame count (Figure):

1) Vessel QFR: QFR value of the entire interrogated segment. Segments proximal to the contoured segment are considered non-stenotic. **Vessel QFR is the value used for clinical decision making in this study and should be entered in the eCRF**
2) Lesion QFR: QFR value of the lesion segment between the two green lesion delimiters.
3) Residual vessel QFR: QFR value of the entire interrogated segment assuming that the lesion segment is treated with stents and becomes healthy segment.
4) Index QFR: QFR value at the white index line placed by the user.

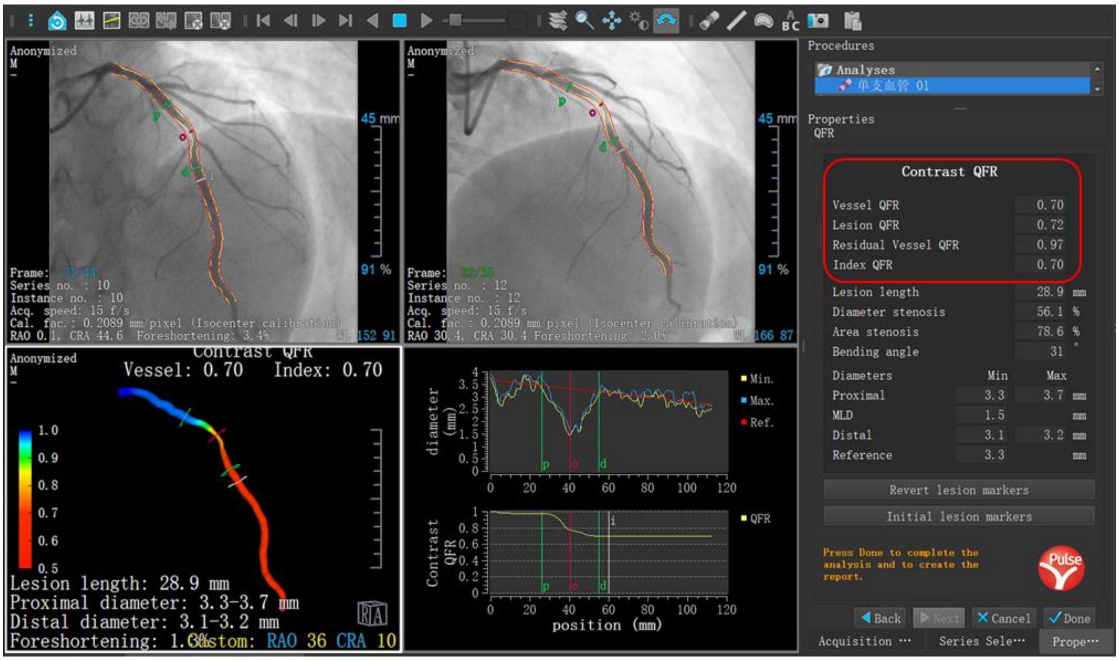

Figure 14. QFR results (red box) including vessel QFR , lesion QFR, residual QFR and index QFR.

Side branch QFR computation:

If there are stenoses in LM/LAD - diagonal branch, we can calculate the vessel QFR by 3 steps:

1) Calculate the vessel QFR of LM/LAD and move white index line to bifurcation of diagonal branch, define the index QFR as QFR1.
2) Calculate the vessel QFR of diagonal branch and define it as QFR2.
3) The final QFR is QFR1+QFR2-1.

The principle is also applicable to LM – LCX lesions.

11. Analysis check

Analyst need to check longitudinal and transverse lumen size curve, QFR pull-back curve and morphology of three-dimensional reconstruction vessel to achieve a good quality of QFR measurement. The general principles are as follows:

1) The variation tendency curve of long-axis and short-axis of lumen size should keep consistent as much as possible (Figure 15);
2) Check the QFR pull-back curve to avoid the “false” positive lesion detection due to inaccuracy of segmentation. Ensure that the QFR value descends at lesion segment and is stable at normal segment;
3) Check if three-dimensional reconstruction vessel is stretched naturally.

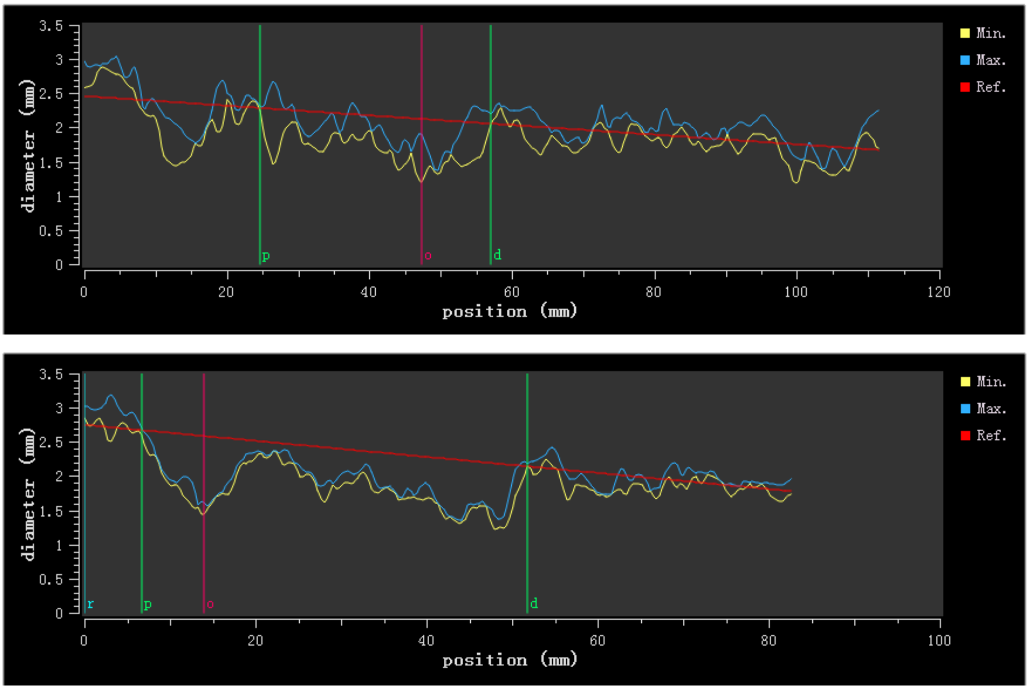

Figure 15. The variation tendency curve of long-axis and short-axis of lumen size should keep consistency as much as possible. (Upper) Inconsistent variation tendency between long-axis and short-axis lumen size curve; (Lower) Consistency variation tendency between long-axis and short-axis lumen size curve.

12. Documentation and report

The final QFR analysis is saved in AngioPlus system by clicking Done in the lower right corner and clicking “Save session as” in the upper panel

(

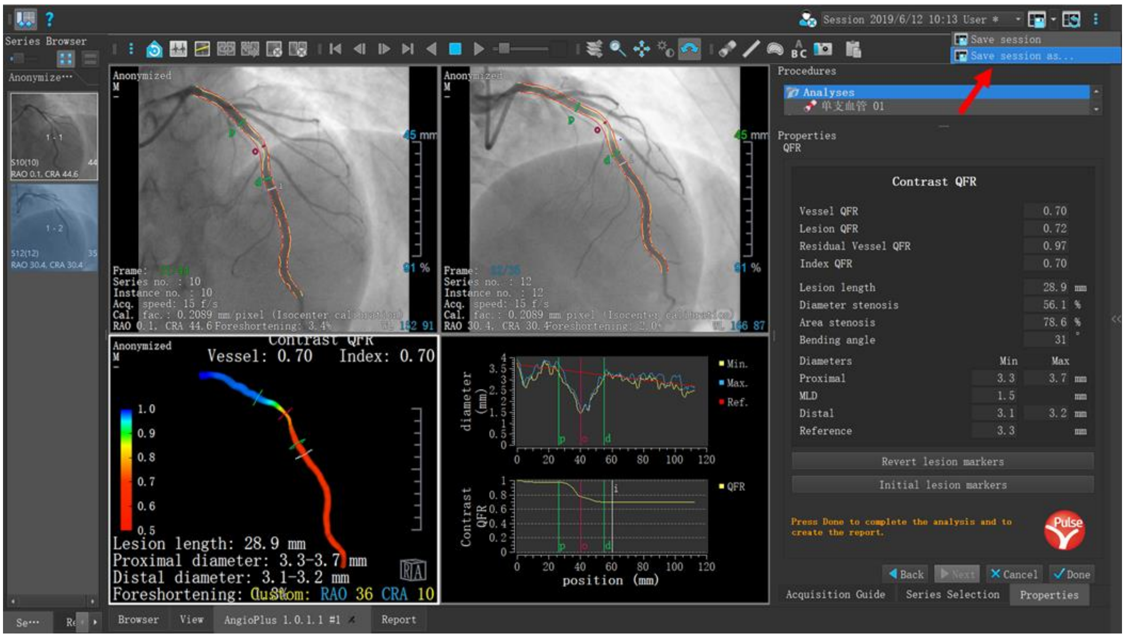

Figure, red marker). A report summarizing the analysis is subsequently generated, including 2D images of the selected projection, the 3D vessel reconstruction and measurements (

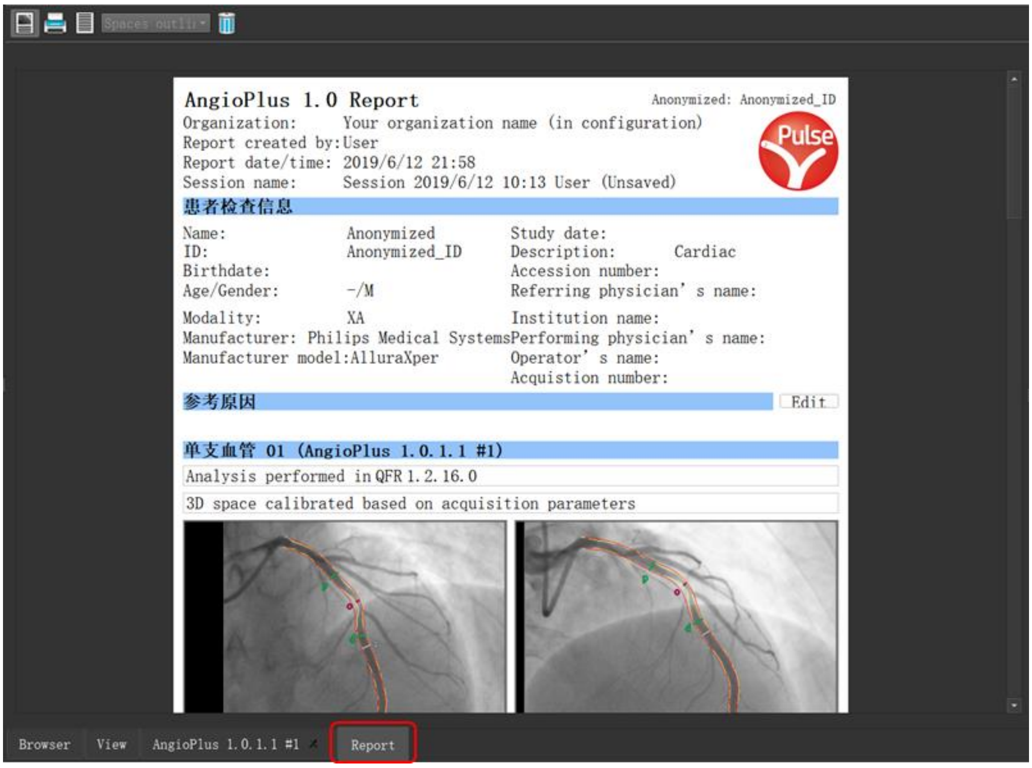

Figure). Analyst should save the analysis process (session format) and analysis report (PDF format), and name it according to the “subject ID + CASS number” format, such as “R0001+01".

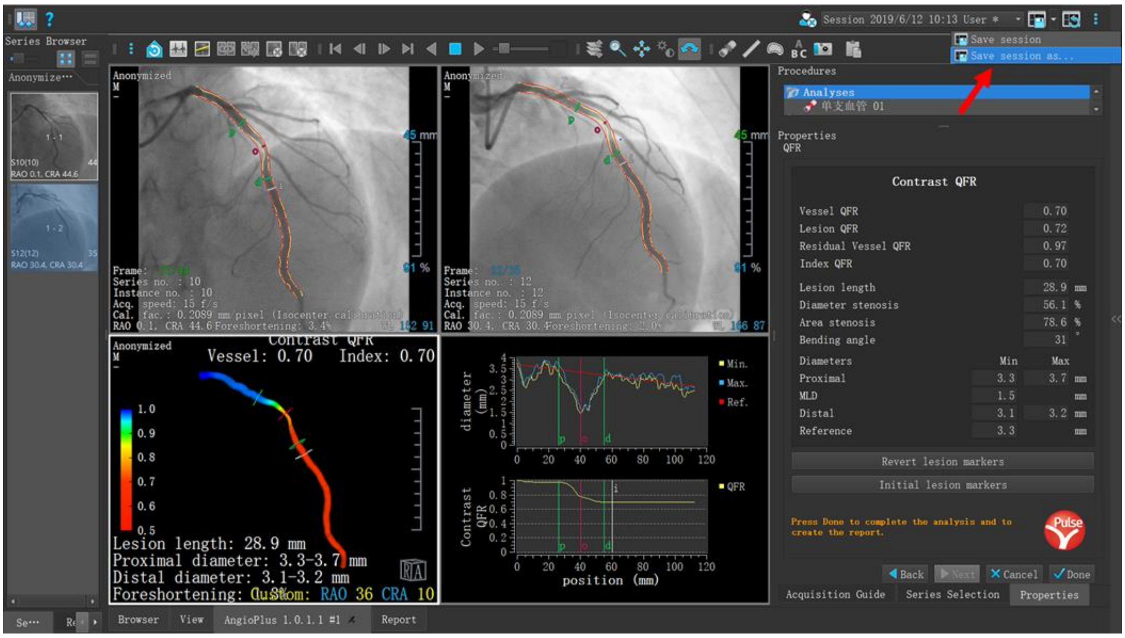

Figure 16. Documentation. How to save the analysis in AngioPlus system.

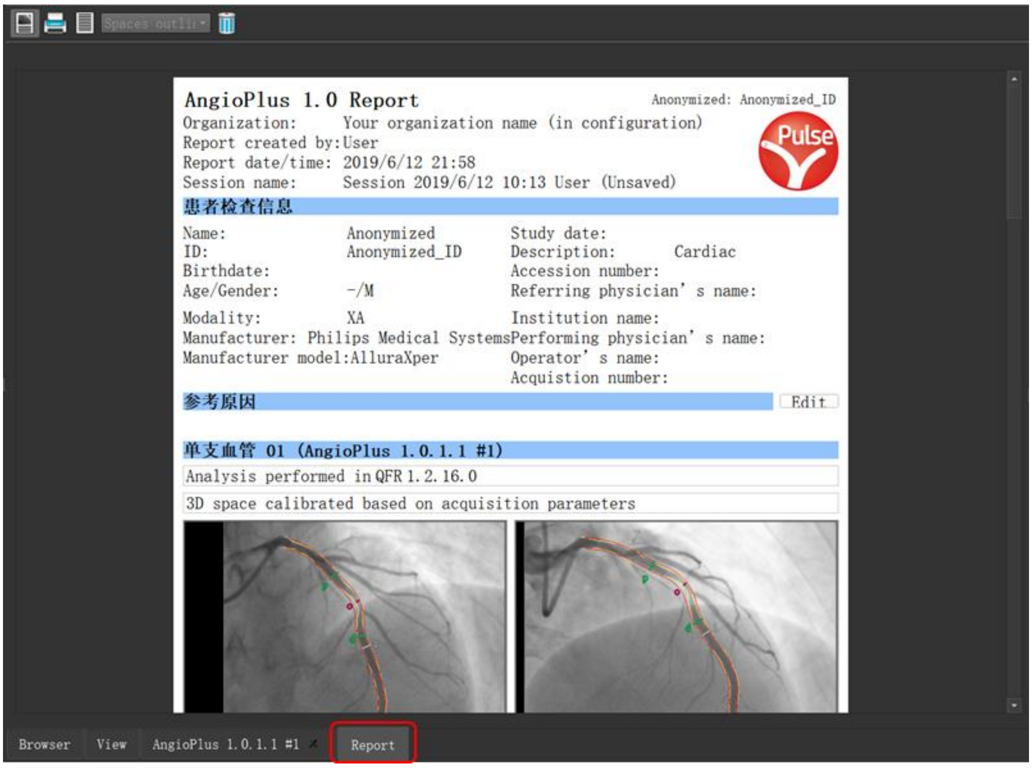

Figure 17. Report. Access the report by selecting the Report pane (red box).

Appendix 2. Blinding and Perception Analysis Questionnaire

Blinding and perception analysis questionnaire administered to the patient at the time of hospital discharge, 6-month, and 1-year follow-up to assess randomization concealment and the perception of randomization allocation
1. Do you think you know to which group you were assigned?<br>a. Yes<br>b. No
If you answered “Yes” to Question #1, then answer the following questions. If you answered “No” to Question #1, do not answer the following questions.
2. Which treatment do you think you received?<br>a. QFR-guided PCI<br>b. Angiography-guided PCI<br>3. Are you certain?<br>a. Yes<br>b. No<br>4. Why do you think you know?<br>a. I was told by/overheard the doctor who did the procedure<br>b. I was told by/overheard another person in the procedure room/cath lab<br>c. I was told by/overheard another person in the hospital before discharge<br>d. I was told by/overheard a family member or friend who was told<br>e. I believe so because I am feeling better<br>f. I believe so because I am not feeling better<br>g. Other (write in)

Appendix 3. Investigator's Agreement

INVESTIGATOR'S AGREEMENT

As an investigator I ensure the following agreement：

1. The present clinical trial will be strictly conducted in accordance with the principles of the Declaration of Helsinki, with the laws and regulations in China, as well as study protocol.

2. The full study dataset will be correctly collected into the web-based electronic data capture (EDC) system, complete the clinical report on time.

3. The tested instruments should be only used in this study, accurately and completely record the storage and usage information during the study.

I have read the attached protocol entitled "Comparison of Quantitative Flow Ratio Guided and Angiography Guided Percutaneous Intervention in Patients with Coronary Artery Disease: FAVOR III China" and agree to abide by all provisions set forth therein.

Signature
Date

Comparison of Quantitative Flow Ratio Guided and Angiography Guided Percutaneous Intervention in Patients with Coronary Artery Disease (FAVOR III China)

Statistical Analysis Plan

V1.0 - Final

STATISCAL ANALYSIS PLAN VERSION AND AMMENDMENT TRACKING

Version Number / Amendment	Approval Date
1.0	May 17, 2021

Statistical Analysis: Medical Research & Biometrics Center, National Center for Cardiovascular Disease, CHINA

Sponsor: Fuwai hospital, Chinese Academy of Medical Sciences

Primary Investigators: Dr. Bo Xu and Dr. Shubin Qiao

SIGNATURE PAGE

Title:	Comparison of Quantitative Flow Ratio Guided and Angiography Guided Percutaneous Intervention in Patients with Coronary Artery Disease (FAVOR III China)	
Sponsor:	Fuwai hospital, Chinese Academy of Medical Sciences	
Protocol Number:	CRFH20180065	
Version Date:	May 17, 2021	
Version No.:	V1.0	
Principal Investigator	Title: Director of Center for Diagnosis and Treatment of Coronary Artery Disease<br>Signature: Shubin Qiao	Date: 30-May-2021
	Title: Director of Catheterization Laboratories<br>Signature: Xu Bo	Date: 30-May-2021
Chair of Statistics	Title: Director of Statistics<br>Signature: Yang Wang	Date: 26-May-2021
Statistician	Title: Statistician<br>Signature: Yanyan Zhao	Date: 26-May-2021

LIST OF ABBREVIATIONS

**Abbreviations**	**Definition**
AE	Adverse Event
ATS	As Treated Set
CEC	Clinical Event Committee
CIs	Confidence Intervals
CRF	Case Report Form
DES	Drug Eluting Stents
DS	Diameter Stenosis
DSMB	Data Safety Monitoring Board
FFR	Fractional Flow Reserve
FPFV	First Visit for the First Patient
HRs	Hazard Ratios
MACE	Major Adverse Cardiovascular Events
MI	Myocardial Infarction
ITT	Intention-to-treat Set
PCI	Percutaneous Coronary Intervention
PPS	Per-protocol Set
QCA	Quantitative Coronary Angiography
QFR	Quantitative Flow Ratio
SAE	Serious Adverse Events
SD	Standard Deviation
SOP	Standard Operation Procedure
TLR	Target Lesion Revascularization
TVR	Target Vessel Revascularization

CONTENTS

SIGNATURE PAGE_ 1
LIST OF ABBREVIATIONS........ 2
CONTENTS 3
1. Introduction 4
2. Trial Design and Objectives 5
2.1 Study Objectives 5
2.2 Study Design 5
2.3 Primary Endpoint 5
2.4 Major Secondary Endpoint 5
2.5 Other Secondary Endpoints 5
3. Inclusion and Exclusion Criteria 7
3.1 Criteria Prior to Informed Consent (General criteria) 7
3.2 Criteria After Enrollment (Informed Consent) and Prior to Randomization .. 7
4. General Statistical Considerations 9
4.1 Sample Size Determination and Assumption 9
4.2 Analysis Population 9
4.3 Identification Details and Flowcharts of Analysis Population 10
4.4 Handling of Missing Data, Unused or Erroneous Data 14
5. Blinding and Unblinding 15
6. Significance Level and Statistical Analysis Software 17
7. Subgroup Analysis 18
8. Interim Analysis 20
9. Indicators and Methods of the Statistical Analysis 21
9.1 Demographic and Baseline Characteristics 21
9.2 Lesion and Treatment Information 21
9.3 Primary Endpoint 21
9.3 Major Secondary Endpoint 22
9.4 Other Secondary Endpoints 22
9.5 Safety Analysis 22
10. Validation Requirements 23
11. References 24
12. Appendix 1. Blinding and Perception Analysis Questionnaire 25

1. Introduction

This document provides details of the statistical analysis plan (SAP) for the FAVOR III China (comparison of quantitative flow ratio guided and angiography guided percutaneous intervention in patients with coronary artery disease) [1]. For the 1-year results and subsequent annual statistical analysis report:

1) The baseline to 1-year follow-up statistical analysis report will be provided after the subjects complete 1 year follow-up or terminate the trial early and the database is hard locked. Subjects and clinical assessor will be blinded to the assignment results until the 1-year follow-up visit for all randomized subjects are completed.

2) The annual statistical analysis report will be subsequently provided after the subjects complete the subsequent annual follow-up (2 years, 3 years) and the database is hard locked.

2. Trial Design and Objectives

2.1 Study Objectives

The objective of the FAVOR III China trial is to investigate whether quantitative flow ratio (QFR)-guided percutaneous coronary intervention (PCI) yields superior clinical outcome and cost-effectiveness compared to standard coronary angiography-guided PCI in patients with coronary artery disease [1].

2.2 Study Design

The FAVOR III China is a prospective, multi-center, subjects and clinical assessors blinded, sham-controlled, superiority test, randomized clinical trial. Participants meeting all the inclusion and exclusion criteria are randomized in a 1:1 ratio to QFR-guided PCI strategy or angiography-guided PCI strategy.

2.3 Primary Endpoint

The primary endpoint is the 1-year rate of major adverse cardiovascular events (MACE), defined as a composite of all-cause mortality, any myocardial infarction (MI) and any ischemia-driven revascularization.

2.4 Major Secondary Endpoint

The major secondary endpoint is the rate of MACE excluding periprocedural MI, defined as a composite of all-cause mortality, any spontaneous MI and any ischemia-driven revascularization at 1 year.

2.5 Other Secondary Endpoints

1) The rate of MACE at 1 month, 6 month, 2 years and 3 years

2) The rate of death (cardiovascular, non-cardiovascular and undetermined) at 1 month, 6 months, 1 year, 2 years and 3 years

3) The rate of MI (target vessel related and non-target vessel related) at 1 month, 6 months, 1 year, 2 years and 3 years

4) The rate of target lesion revascularization (TLR) (ischemia-driven and non-ischemia-driven) at 1 month, 6 months,1 year, 2 years and 3 years

5) The rate of target vessel revascularization (TVR) (ischemia-driven and non-ischemia-driven) at 1 month, 6 months,1 year, 2 years and 3 years

6) The rate of any coronary artery revascularization (ischemia-driven and non-ischemia driven) at 1 month, 6 months,1 year, 2 years and 3 years

7) The rate of definite or probable stent thrombosis during acute, sub-acute, late, and very late phase according to the Academic Research Consortium (ARC)-2 definition [2], at 1 month, 6 months,1 year, 2 years and 3 years

8) PCI strategy changes after three-dimension quantitative coronary angiography (3D-QCA): comparing the changes on the strategy including devices size after 3D-QCA guided versus angiography guided PCI

9) Health economics endpoints: Cost-effectiveness analysis at 1 month, 6 months, 1 year

3. Inclusion and Exclusion Criteria

Patients enrolled should meet all the following inclusion/exclusion criteria:

3.1 Criteria Prior to Informed Consent (General criteria)

Patients will be screened for the following inclusion and exclusion criteria:

- **Inclusion (pre informed consent)**

1. Age ≥ 18 years.
2. With stable or unstable angina pectoris, or with post-acute myocardial infarction (≥ 72 hours).
3. Able to understand the trial design and provide written informed consent.
4. Eligible for PCI by the operator assessment.

- **Exclusion (pre informed consent)**

1. Cardiogenic shock or severe heart failure (NYHA ≥III).
2. Severely impaired renal function: creatinine >150μmol/L or Cockcroft-Gault calculated GFR <45 ml/kg/1.73 m^2 (calculated with Cockcroft-Gault formula).
3. Allergy to iodine-containing contrast agents which cannot be adequately premedicated.
4. Pregnancy or intention to become pregnant during the course of the trial.
5. Life expectancy less than one year.

3.2 Criteria After Enrollment (Informed Consent) and Prior to Randomization

Participants who provide informed consent and are clinically eligible will undergo coronary angiography assessments. Participants meeting the angiographic inclusion criteria, and not meeting any of the following exclusion criteria will be randomized.

- **Angiographic inclusion criteria**

With at least one lesion of 50%-90% diameter stenosis (DS) in 1 coronary artery with ≥ 2.5mm reference vessel diameter by visual assessment.

- **Angiographic exclusion criteria**

1. Patients with only one coronary artery lesion with DS >90% with TIMI flow <3.

2. An interrogated lesion is at the site of a myocardial bridge.
3. An interrogated lesion is a culprit lesion related to acute MI.
4. An interrogated lesion is in a bypass graft.
5. Poor angiographic image quality precluding vessel contour detection or with suboptimal contrast opacification.
6. Severe overlap in the stenosed segment or severe tortuosity of any interrogated vessel deemed not amenable to QFR measurement.

4. General Statistical Considerations

4.1 Sample Size Determination and Assumption

Sample size calculation is based on primary endpoint as the 1-year rate of MACE.

The corresponding null hypothesis to be tested is that the difference of 1-year MACE between test group and control group (P_T-P_C) is equal or greater than 0, versus the alternative hypothesis is that the difference is less than 0.

H_0: $P_T - P_C \geq 0$;
H_1: $P_T - P_C < 0$;

where P_T and P_C denotes MACE at 1 year in QFR-guided PCI group and angiography-guided PCI group, respectively.

Assume a 1-year risk of MACE in standard angiography-guided PCI group of 8.7% according to a conservative estimate of 1-year MACE from a 5,000-patient cohort of 2 multicenter, all-comers clinical trials, PANDA III (Comparison of BuMA eG Based BioDegradable Polymer Stent With EXCEL Biodegradable Polymer Sirolimus-eluting Stent in “Real-World” Practice) [3] and I-LOVE-IT 2 (Evaluate Safety and Effectiveness of the Tivoli DES and the Firebird 2 DES for Treatment of Coronary) [4] trials, as in those 2 trials <1% FFR was used. And assume a 1-year risk of MACE in QFR-guided PCI group of 6.1% according to clinical trials such as DEFINE-FLAIR trial [5] and iFR-SWEDEHEART trial [6]. Using a 2.5% level of one-sided significance and a maximum possible loss to follow-up rate of 5%, 3,830 participants (1,915 in each group) will provide 85% power to demonstrate that the QFR-guided strategy is superior to the angiography-guided strategy for the primary end point.

For the major secondary end point, the rate of MACE excluding periprocedural MI (ie, all-cause death, spontaneous MI, and any ischemia-driven revascularization) was 6.0% based on PANDA III [3] and I-LOVE-IT 2 trials [4]. Assuming an incidence in the QFR-guided arm of 4.0% (33% relative risk reduction), using a 2.5% level of 1-sided significance and a maximal loss to follow-up rate of 2.5%, a total of 3,830 patients will provide 80% power to demonstrate superiority.

4.2 Analysis Population

The statistical analysis will be carried out on the basis of the following analysis population. Analysis population is clearly defined before starting the analysis. The analysis population includes:

Intention-to-treat (ITT): The intention-to-treat population consists of patients undergoing randomization but excluding those with randomization error or informed consent withdrawal prior to index PCI.

Per-protocol Set (PPS): The per-protocol population consists of patients who were successfully randomized and underwent the treatment strategy excluding those with major protocol deviations (including key inclusion/exclusion criteria violation, any intended vessels not treated in the control group, treatment not performed based on QFR measurements, the QFR measurement not performed per protocol in the test group, etc). Major protocol deviations will be finally adjudicated by steering committee and statistician by discussion.

As Treated Set (ATS): The as treated population consists of patients who are analyzed according to the actual strategy (angiography or QFR guidance) used for treatment rather than their randomization assignment.

Primary and major secondary endpoints will be analyzed in ITT, PPS and ATS (if applicable). The principal analyses of the primary and secondary endpoints will be performed by ITT.

4.3 Identification Details and Flowcharts of Analysis Population

1) Informed consent withdrawal: subjects obtained randomization number, but withdrawal prior to index PCI and did not receive any treatment based on protocol.

   ITT= Number of the randomized subjects – Number of patients who informed consent withdrawal.

2) Major protocol deviations including:

   a) Key inclusion/exclusion criteria violation: subjects did not meet the inclusion criteria or meet the exclusion criteria set in the protocol, and this protocol deviation severely affect the results of primary endpoint.

   b) Any intended vessels were not treated in the control group, or

   c) Any intended vessels were not treated based on QFR measurements in the test group (including treatment not performed based on QFR measurements, or the QFR measurement not performed per protocol).

3) Cross-over: the subjects were randomly assigned to the test group, but treated based on control strategy actually; or subjects were randomly assigned to the control group, but treated based on test strategy actually.

PPS = ITT - Number of subjects who had major protocol deviations.

ATS = PPS + Number of subjects who were only cross-overed. If there are no cross-over, the ATS will not be provided.

Number of subjects who had major protocol deviations = Number of subjects who had key inclusion/exclusion criteria violation + Any intended target lesions not treated + Cross-over.

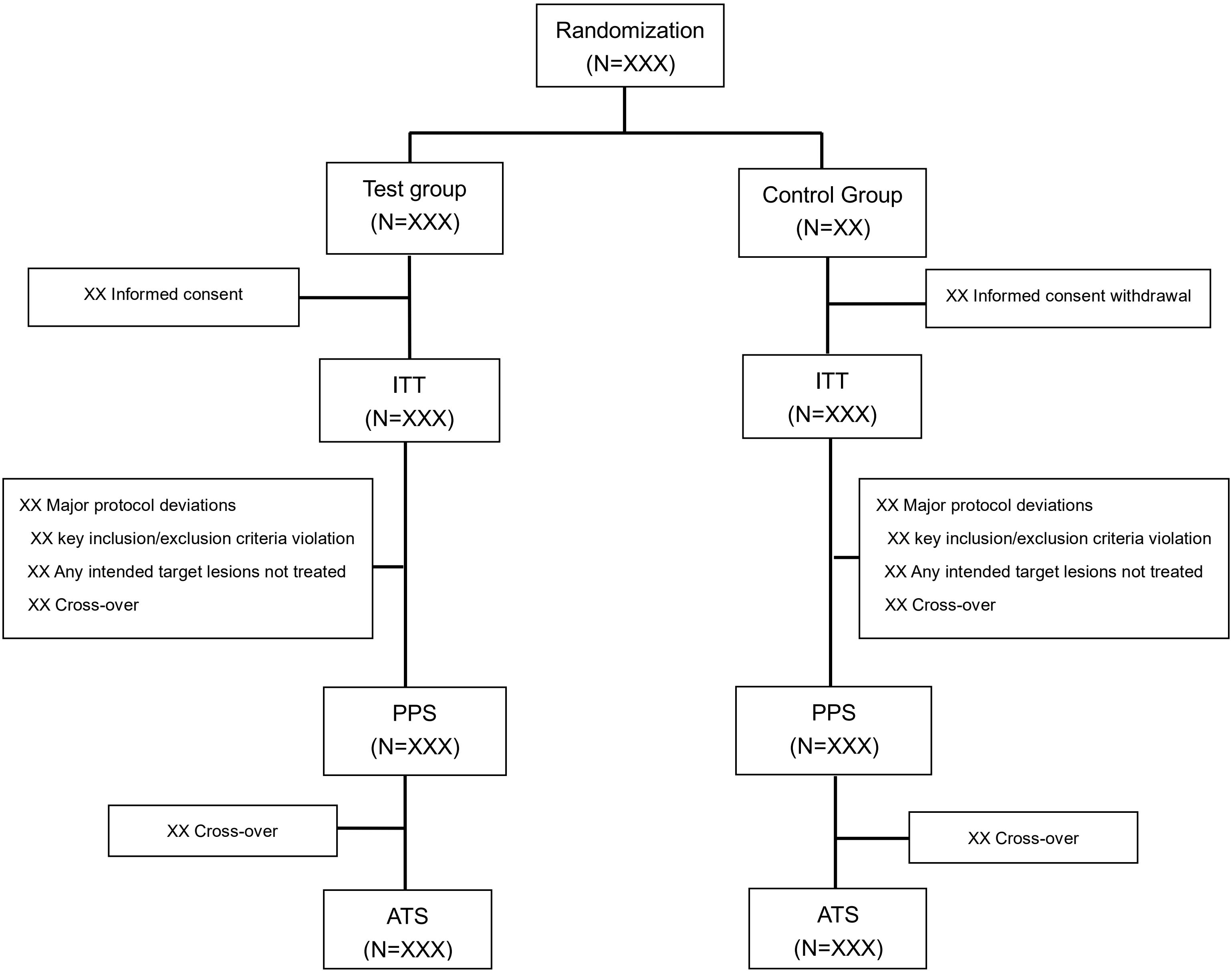

Figure 1 Flow Chart of Analysis Population Determination

Note: Test group: QFR-guided PCI, Control group: Angiography-guided PCI.

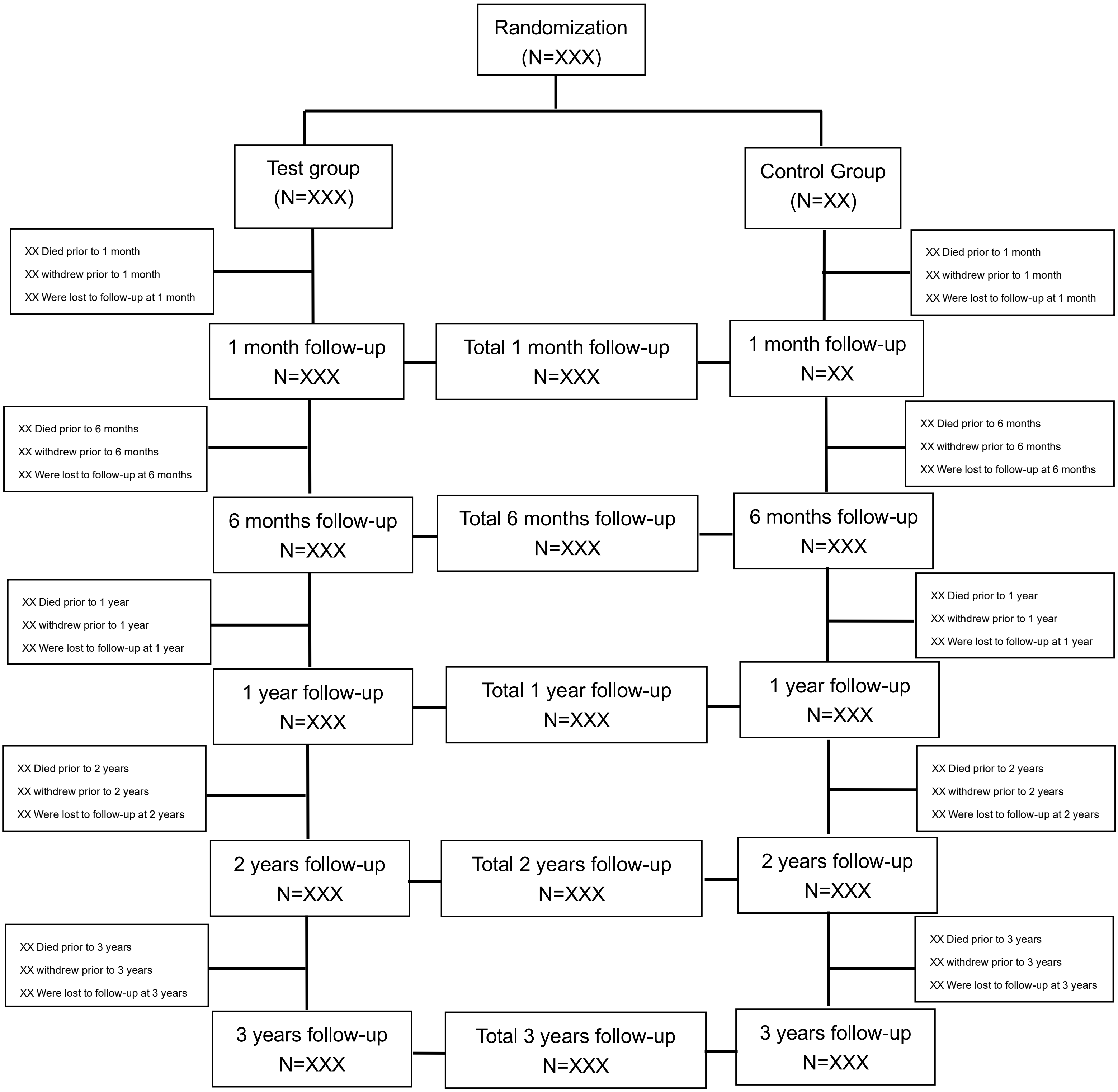

Figure 2 Subject Disposition Flow Chart Post-Procedure – Clinical Follow-up

Note: Test group: QFR-guided PCI, Control group: Angiography-guided PCI.

Lost to follow-up at 1 month/6 months/1 year/2 years/3 years: subjects had no 1 month/6 months/ 1 year/2 years/3 years contact.
Withdrew prior to 1 month/6 months/1 year/2 years/3 years: subjects had premature discontinuation prior to 1 month/6 months/ 1 year/2 years/3 years contact.

The table below is the follow-up visit windows for endpoint and follow-up rate analysis.

Follow-up interval	Study Time Window Post Procedure for endpoint	Study Time Window Post Procedure for follow-up rate
1 Month	30 days	30 ± 7 days
6 Month	180 days	180 ± 30 days
1 Year	365 days	365 ± 30 days
2 Years	730 days	730 ± 30 days
3 Years	1095 days	1095 ± 30 days

4.4 Handling of Missing Data, Unused or Erroneous Data

Missing data will not be imputed or replaced. Missing event data for the primary and secondary endpoints will be “censored” at the time of last follow-up in Kaplan-Meier survival analyses and Cox proportional hazards model analyses.

Erroneous or spurious data will be cleaned before performing statistical analysis. The data of withdrawal subjects will also be included in the final statistical analysis. Reasons for withdrawal will be specified in the statistical report. The missing of primary endpoint data caused by early withdrawal will be handled with the methods for missing data as mentioned above.

Screening and acceptance testing of these data will be carried out in accordance with Data Management Plan. To this end, all data involved in the determination of endpoints will be screened for missing and unusual values. Any missing data that affect the ability to determine or analyze any endpoint will be queried by Data Management for confirmation of irretrievability. Unusual values, such as outliers, will also be queried, and if confirmed, will be used as recorded.

5. Blinding and Unblinding

In the FAVOR III China trial, subjects and clinical assessors (including the follow-up research personnel, CEC, and imaging core laboratory) will be blinded to randomization arm. All study site personnel will receive training on the blinding measures before trial initiation. In addition to standard procedural sedation, music-playing headphones will be worn by the patient during the whole procedure, and patients in both groups will undergo a preset 10-minute delay for QFR calculation before the PCI procedure, and a lesion/device evaluation form is required to be filled during said period in both groups, to reduce the possibility of unblinding. Results of QFR calculations will not be reported in the patient file.

The operators and personnel involved in the PCI procedure will not be blinded and therefore will not take part in clinical follow-up. Clinical follow-up visits will be conducted by the site personnel blinded to the randomized results and using the same visit form. The blinding will be maintained until completion of the 1-year follow-up visit for all registered subjects. All subjects were required to complete a questionnaire (Appendix 1) at post-procedure, 6 months and 1-year follow-up to assess the success of the blinding procedures. The blinding assessments will be reported when the 1-year follow-up visit completed.

In addition, the statistician who writes the statistical analysis plan before data analysis, the statistician/SAS programmer who take part in data analysis during data analysis stage will not know the grouping information in advance to avoid the influence of statistical analysts on the trial results and possible bias. During the 1-year follow-up analysis, the following unblinding procedure will be followed.

1) The data manager exports the test data and sends to statistician/SAS programmer during data cleaning stage for preparing programming of data analysis. The test datasets will contain only a few observations and will not contain group information or other indicators information related to group. The statistician/SAS programmer simulates group information and performs data analysis.

2) The data manager exports the database containing all observations after the database is cleaned up and hard locked, and sends to statistician/SAS programmer for programming debugging and data analysis. The datasets will also not contain group information or other indicators information related to group.

3) The statistician/SAS programmer performs statistical analysis based on cleaned datasets and simulated group information according to the SAP until related tables, listings and figures are completed and the SAS programming codes are fine.

4) The unblinding procedure will be performed in an independent data review meeting which the steering committee, international advisory board, statisticians, data managers participate. The statistician who generates the randomization form will send the true randomized group information to statistician/SAS programmer who take part in data analysis to provide the true analysis result of primary endpoint in this meeting. The difference and 95% CI of primary endpoint between test group and control group will be provided on the spot to determine whether the superiority conclusion will be obtained.

5) Complete the signature page and minutes of the data review meeting, unblinding signature page, analyses of other indicators and other subsequently related matters.

6. Significance Level and Statistical Analysis Software

The primary and major secondary end point analyses will be performed at a significance level of 1-sided 0.025. The other statistical analyses will be performed at a significance level of 2-sided 0.05. All tests will be performed using SAS software, version 9.4 (SAS Institute, Cary, NC).

7. Subgroup Analysis

The study includes a prespecified subgroup analysis of the primary endpoint. Analyses will be based on the ITT population.

1) Age: ≥65 vs. <65

2) Gender: Male vs. Female

3) Diabetes mellitus: Yes vs. No

4) Acute coronary syndrome: Yes vs. Non-ACS

5) Smoking history: current vs. former vs. never

6) Body mass index (BMI): ≥30 Kg/m^2 vs. <30 Kg/m^2

7) Left ventricular ejective fraction (LVEF): >45% vs. ≤45%

8) Lesion location: Left main/ proximal anterior descending artery vs. others

9) Lesion length: ≥20 mm vs. <20 mm

10) Reference vessel diameter: ≥2.5 mm vs. < 2.5 mm by QCA

11) Stenosis severity: DS ≥70% vs. <70% by QCA

12) Multivessel disease: Yes vs. No

13) Calcified lesion: Severe calcification vs. non-severe calcification

14) Bifurcation lesion: Yes vs. No

15) Tandem lesion: Yes vs. No

16) Bending/tortuous lesion: Yes vs. No

17) QFR gray zone: QFR < 0.75 vs. = 0.75-0.85 vs. > 0.85 by core laboratory analysis

18) QFR based functional SYNTAX score (FSS_{QFR}): low tertile vs. intermediate tertile

vs. high tertile by core lab

19) Residual QFR (rQFR): ≥ cutoff value vs. < cutoff value by core laboratory

20) QFR based residual functional SYNTAX score ($rFSS_{QFR}$): $rFSS_{QFR}$ = 0 vs. ≥1 by core laboratory

21) Center experience for invasive physiology: experienced centers vs. less experienced centers, experienced center defined as the number of FFR/ iFR > 100 per year

22) Learning experience with QFR: first-half enrolled cases in each center vs. second-half enrolled cases in each center

8. Interim Analysis

Interim analysis and corresponding early termination criteria are not planned for this trial. So, this section is not applicable.

9. Indicators and Methods of the Statistical Analysis

9.1 Demographic and Baseline Characteristics

Demographic information (age, gender), medical history, preoperative examination and other baseline data will be analyzed based on ITT population.

Categorical variables will be summarized with counts and percentages from each category. Continuous variables will be summarized using the number of observations, number of missing data, mean, standard deviation (SD), median, minimum, maximum, the 25th and the 75th percentiles, as well as confidence intervals (CIs) or confidence limits where applicable. Based on the descriptive analysis, categorical data will be compared between the two groups using likelihood ratio chi-square test, and when more than 25% of the cells of the contingency table have a frequency less than 5, then Fisher's exact test will be used. Continuous variables with normal distribution will be tested using two sample *t*-test, and non-normal continuous data will be tested with Wilcoxon Rank Sum test.

9.2 Lesion and Treatment Information

The descriptive analyses of lesion and corresponding treatment information will be performed on ITT at patient, vessel, or lesion level. The total number of treated lesions and stents will be analyzed based on patient level. The detailed lesion information (length of lesion, reference vessel diameter etc.) will be analyzed based on lesion level. The detailed stent information (length of stent, stent diameter, etc.) will be analyzed based on stent level. The statistical analysis method is referred to 9.1.

9.3 Primary Endpoint

The analysis of 1-year MACE will be performed on ITT, PPS and ATS (if applicable). The principal analyses of the primary and secondary endpoints will be performed by ITT. The event count and percentage of MACE at 1 year will be provided. The analyses based on Kaplan-Meier survival analysis and Cox proportional hazards model will be provided as the primary analysis. The cumulative incidence curves for each group will be provided using Kaplan-Meier estimates and compared by the log-rank test. Estimates of the hazard ratios (HRs) and 2-sided 95% CIs will be calculated using a Cox proportional hazards model. Meanwhile, Cochran-Mantel-Haenszel $\chi 2$ test with center effect adjustment will be used to calculate event rate differences and corresponding 2-sided 95% CI between test and control group as the sensitivity

analysis. The analyses based on multivariate Cox proportional hazards model with center, diabetes mellitus, single vessel disease, the presence of DS >90% and TIMI blood flow <3 adjustment will also be performed as the sensitivity analysis. If the upper limit of the 2-sided 95% CI of the HR in 1-year MACE between QFR-guided and angiography-guided group is below 1, then the superiority conclusion will be confirmed.

In addition, if the actual results show that other baseline variables (such as age, gender, etc.) are significantly different between the test group and the control group, the statistician will fully communicate with investigator during data analysis stage. The true confounding factors will be further clarified by the univariate analysis result of baseline variables and primary endpoint, previous published literature and combined with the experience of clinical experts. These indicators will be considered to be included in the model for adjustment as a sensitivity analysis for the analysis of primary endpoint.

9.3 Major Secondary Endpoint

The analysis of 1-year rate of MACE excluding periprocedural MI will also be performed on ITT, PPS and ATS (if applicable). The event count and percentage of MACE at 1 year, event rate differences and corresponding 95% CIs between test and control group will also be provided. The analysis strategy of major secondary endpoint will be the same as primary endpoint.

9.4 Other Secondary Endpoints

For other secondary endpoints, the descriptive analysis results will be provided and the statistical analysis method is referred to 9.1. For the time-to-event outcomes, the cumulative incidence curves for each group will be provided using Kaplan-Meier estimates and compared by the log-rank test. Estimates of the HRs and 95% CIs will be calculated using a Cox proportional hazards model.

9.5 Safety Analysis

Adverse events will be reported with event counts and percentages, and will be compared using likelihood ratio chi-square test or Fisher exact test. Meanwhile, the symptom, severity and the relation to investigational devices of all adverse events will be described in a detailed manner.

10. Validation Requirements

All the output of analysis described above need to be validated by independent biostatistician and/or SAS programmer and all the output can be available to be delivered as long as validated.

11. References

1. Song L, Tu S, Sun Z, et al. Quantitative flow ratio-guided strategy versus angiography-guided strategy for percutaneous coronary intervention: Rationale and design of the FAVOR III China trial. American heart journal 2020;223:72-80.

2. Garcia-Garcia HM, McFadden EP, Farb A, et al. Standardized End Point Definitions for Coronary Intervention Trials: The Academic Research Consortium-2 Consensus Document. European heart journal 2018;39:2192-207.

3. Xu B, Gao R, Yang Y, et al. Biodegradable Polymer-Based Sirolimus-Eluting Stents With Differing Elution and Absorption Kinetics: The PANDA III Trial. Journal of the American College of Cardiology 2016;67:2249-58.

4. Han Y, Xu B, Jing Q, et al. A randomized comparison of novel biodegradable polymer- and durable polymer-coated cobalt-chromium sirolimus-eluting stents. JACC Cardiovascular interventions 2014;7:1352-60.

5. Davies JE, Sen S, Dehbi HM, et al. Use of the Instantaneous Wave-free Ratio or Fractional Flow Reserve in PCI. N Engl J Med 2017;376:1824-34.

6. Gotberg M, Christiansen EH, Gudmundsdottir IJ, et al. Instantaneous Wave-free Ratio versus Fractional Flow Reserve to Guide PCI. N Engl J Med 2017;376:1813-23.

12. Appendix 1. Blinding and Perception Analysis Questionnaire

Blinding and perception analysis questionnaire administered to the patient at the time of hospital discharge, 6-month, and 1-year follow-up to assess randomization concealment and the perception of randomization allocation
1. Do you think you know to which group you were assigned?<br>a. Yes<br>b. No
If you answered “Yes” to Question #1, then answer the following questions. If you answered “No” to Question #1, do not answer the following questions.
2. Which treatment do you think you received?<br>a. QFR-guided PCI<br>b. Angiography-guided PCI<br>3. Are you certain?<br>a. Yes<br>b. No<br>4. Why do you think you know?<br>a. I was told by/overheard the doctor who did the procedure<br>b. I was told by/overheard another person in the procedure room/cath lab<br>c. I was told by/overheard another person in the hospital before discharge<br>d. I was told by/overheard a family member or friend who was told<br>e. I believe so because I am feeling better<br>f. I believe so because I am not feeling better<br>g. Other (write in)
