# Supplementary material for: Prognostic impact of guideline-directed medical therapy after functionally complete revascularisation in patients with obstructive coronary artery diseases
Source: Heart. 2025 Aug 7;112(5):e325670. doi: 10.1136/heartjnl-2025-325670 (PMC13271866; doi:10.1136/heartjnl-2025-325670)
Supplement: online supplemental file 2 [file heartjnl-112-5-s002.docx]

**Supplement**

**Full title: Prognostic Impact of Guideline-Directed Medical Therapy After Functionally Complete Revascularization in Patients with Obstructive Coronary Artery Diseases**

**Authors:** Yingyang Geng, MD, Changdong Guan, MSc, Yao Jiang, MD, Weixian Yang, MD, Bo Yu, MD, Guosheng Fu, MD, Jun Pu, MD, Xinkai Qu, MD, Qi Zhang, DM, Yanyan Zhao, MSc, Lilei Yu, MD, Yunfei Huang, PhD, Shengxian Tu, PhD, Shubin Qiao, MD, Lei Song, MD, and the FAVOR III China Study Group

[Angiography and QFR Measurements 2](#_Toc193833854)

[Angiography Acquisition 2](#_Toc193833855)

[QFR System 2](#_Toc193833856)

[QFR Measurements 2](#_Toc193833857)

[eTable 1. Guideline-directed medical therapy following PCI 3](#_Toc193833858)

[eTable 2. Baseline Before and after propensity Score Matching 5](#_Toc193833859)

[eTable 3. Subgroup Analysis: GDMT's effects on clinical endpoints in ACS and CCS patients 6](#_Toc193833860)

[eTable 4. Sensitivity analysis of the effect of QFR guidance and angiography guidance on outcome events. 7](#_Toc193833861)

[eTable 5. Sensitivity analysis of the clinical outcome events over 3 years in patients achieving FCR after PCI. 8](#_Toc193833862)

[eFigure 1. Percent of participants adhered to GDMT through 3 years. 9](#_Toc193833863)

[eFigure 2. Effect of GDMT on MACCE at each follow-up timepoint. 10](#_Toc193833864)

[eFigure 3. Proportion of participants who achieved a given number of GDMT goals over the follow-up period. 11](#_Toc193833865)

[eFigure 4. Percent of participants adhered to each prescribed agents through 3 years. 12](#_Toc193833866)

[eFigure 5. Sensitivity analysis for varying GDMT intensity and its impact on MACCE. 13](#_Toc193833867)

[eFigure 6. Sensitivity analysis of prescribed medications on MACCE at each follow-up timepoint. 14](#_Toc193833868)

# Angiography and QFR Measurements

## Angiography Acquisition

Patients enrolled in the study will perform coronary angiography according to standard operation procedure to acquire projections with minimal overlap in the target vessel. After administration of nitroglycerin, two angiographic image runs with at least 25° deviation in projection angles are required. The recommended angiographic projections for angiographic image acquisition should be use:

| **Interrogated Vessel** | **1^st^ view** | **2^nd^ view** |
| --- | --- | --- |
| LM and LM bifurcation | 20º RAO, 45º Caudal | 0º LAO, 10º Caudal |
| LAD and diagonal artery | 0º LAO, 45º Cranial | 30º RAO, 20º Cranial |
| LCX and OM | 10º LAO, 25ºCaudal | 25º RAO, 35º Caudal |
| RCA/PLA/PDA | 45º LAO, 10º Caudal | 20º LAO, 20º Cranial |

## QFR System

QFR is measured through AngioPlus system (Pulse Medical Imaging Technology, Shanghai Co., Ltd., Shanghai, China), consists of a display screen, a host and QFR measurement software, according to the standard operation procedure. Two angiographic image runs with at least 25° deviation in projection angles are transferred through local network to AngioPlus system. After completing the QFR analysis, save the results for subsequent research.

## QFR Measurements

The principle of QFR measurement system: The AngioPlus system receives two angiographic image runs with at least 25° deviation in projection angles, uses the centerline and contour detection algorithms to complete the delineation of the target vessel and 3D reconstruction automatically. The QFR value of the target vessel is obtained based on the TIMI frame count and optimized coronary hemodynamic model, and the analysis results can be summarized in the report to provide the doctor with auxiliary diagnosis and treatment information. The workflow of QFR measurement software is as follows：

Step1: Select a pair of calibration points with significant anatomical features to perform geometric position calibration on the two image sequences;

Step2: Use the verified centerline and contour detection algorithms to complete the delineation of the target vessel automatically, and combine the 3D reconstruction algorithm to reconstruct and display the target vessel and its reference lumen;

Step3: The analyst selects the image where the contrast enters the target vessel segment as the starting frame, and the contrast flowing out of the target vessel segment as the end frame. The QFR measurement system automatically calculates QFR value at each position of the target vessel and displays pull back curve;

# eTable 1. Guideline-directed medical therapy following PCI

|  | **STEMI** | **Non-STE ACS** | **CCS** |
| --- | --- | --- | --- |
| **Antiplatelet drugs** |  |  |  |
| Aspirin | - Antiplatelet therapy with low-dose aspirin (75-100 mg) is indicated. *(Level of Evidence: IA)* | - Aspirin is recommended for all patients without contraindications at an initial oral LD of 150-300 mg (or 75-250 mg i.v.), and at a MD of 75-100 mg o.d. for long-term treatment. *(Level of Evidence: IA)* | - Aspirin 75-100 mg daily is recommended following stenting. *(Level of Evidence: IA)* |
| P2Y12 receptor inhibitor | - DAPT in the form of aspirin plus ticagrelor or prasugrel (or clopidogrel if ticagrelor or prasugrel are not available or are contraindicated), is recommended for 12 months after PCI, unless there are contraindications such as excessive risk of bleeding. *(Level of Evidence: IA)* | - A P2Y12 receptor inhibitor is recommended in addition to aspirin, and maintained over 12 months unless there are contraindications or an excessive risk of bleeding. *(Level of Evidence: IA)* | - Clopidogrel 75 mg daily following appropriate loading (e.g. 600 mg or >5 days of maintenance therapy) is recommended, in addition to aspirin, for 6 months following coronary stenting, irrespective of stent type, unless a shorter duration (1-3 months) is indicated due to risk or the occurrence of life-threatening bleeding; *(Level of Evidence: IA)* - Prasugrel or ticagrelor may be considered, at least as initial therapy, in specific high-risk situations of elective stenting (e.g. suboptimal stent deployment or other procedural characteristics associated with high risk of stent thrombosis, complex left main stem, or multivessel stenting) or if DAPT cannot be used because of aspirin intolerance. *(Level of Evidence: IIb C)* |
| **β-blocker** | - Oral treatment with beta-blockers is indicated in patients with heart failure and/or LVEF <40% unless contraindicated. *(Level of Evidence: IA)* | - Beta-blockers are recommended in patients with systolic LV dysfunction or heart failure with reduced LVEF (<40%); *(Level of Evidence: IA)* - In patients with prior MI, long-term oral treatment with a beta-blocker should be considered in order to reduce all-cause and cardiovascular mortality and cardiovascular morbidity. *(Level of Evidence: IIa B)* | - Beta-blockers are recommended in patients with LV dysfunction or systolic HF; *(Level of Evidence: IA)* - In patients with a previous STEMI, long-term oral treatment with a beta-blocker should be considered. *(Level of Evidence: IIa B)* |
| **ACEI/ARB** | - ACE inhibitors are recommended, starting within the first 24 h of STEMI in patients with evidence of heart failure, LV systolic dysfunction, diabetes, or an anterior infarct; *(Level of Evidence: IA)* - An ARB, preferably valsartan, is an alternative to ACE inhibitors in patients with heart failure and/or LV systolic dysfunction, particularly those who are intolerant of ACE inhibitors. *(Level of Evidence: IB)* | - ACE inhibitors (or ARBs in cases of intolerance to ACE inhibitors) are recommended in patients with heart failure with reduced LVEF (<40%), diabetes, or CKD unless contraindicated (e.g. severe renal impairment, hyperkalaemia, etc.) in order to reduce all-cause and cardiovascular mortality and cardiovascular morbidity. *(Level of Evidence: IA)* | - ACE inhibitors (or ARBs) are recommended in the presence of other conditions (e.g. HF, hypertension, or diabetes). *(Level of Evidence: IA)* |
| **Statin** | - It is recommended to start high-intensity statin therapy as early as possible, unless contraindicated, and maintain it long term. *(Level of Evidence: IA)* | - Statins are recommended in all NSTE-ACS patients. The aim is to reduce LDL-C by >50% from baseline and to achieve LDL-C <1.4 mmol/L (<55 mg/dL). *(Level of Evidence: IA)* | - Statins are recommended in all patients with CCS. *(Level of Evidence: IA)* |

ACE=angiotensin-converting enzyme; ACS=acute coronary syndromes; ARB=angiotensin II receptor blocker; CCS=chronic coronary syndrome; CKD=chronic kidney disease; DAPT=dual antiplatelet therapy; HF=heart failure; LD=loading dose; LDL-C=low-density lipoprotein cholesterol; LV=left ventricular; LVEF=left ventricular ejection fractions; MD=maintenance dose; PCI=percutaneous coronary intervention; NSTE=non-ST-segment elevation; STEMI=ST-segment elevation myocardial infarction.

# eTable 2. Baseline Before and after propensity Score Matching

|  | **SD before PSM** | **SD after PSM** |
| --- | --- | --- |
| Age(years) | 0.165 | 0.004 |
| Diabetes mellitus | 0.711 | 0.027 |
| Hypertension | 0.18 | 0.145 |
| Hyperlipidemia | 0.202 | 0.123 |
| Smoking | 0.119 | 0.041 |
| Family history of CAD | 0.117 | 0.047 |
| Previous MI | 0.185 | 0.052 |
| Stable angina | 0.109 | 0.013 |
| Acute coronary syndromes | 0.208 | 0.007 |
| PCI performed | 0.275 | 0.085 |

SD=Standardized Difference; PSM=propensity score matching; CAD=coronary artery disease; MI=myocardial infarction; PCI=percutaneous coronary intervention.

# eTable 3. Subgroup Analysis: GDMT's effects on clinical endpoints in ACS and CCS patients

|  | **ACS** | | **CCS** | | **P for interaction** |
| --- | --- | --- | --- | --- | --- |
|  | **Adjusted HR**  **(95% CI)** | ***P* value** | **Adjusted HR**  **(95% CI)** | ***P* value** |  |
| MACCE | 0.58 (0.42, 0.78) | <0.01 | 0.47 (0.32, 0.69) | <0.01 | 0.57 |
| MACCE excluding PMI | 0.50 (0.35, 0.71) | <0.01 | 0.34 (0.21, 0.55) | <0.01 | 0.28 |
| All-cause death | 1.43 (0.60, 3.44) | 0.42 | 0.68 (0.21, 2.25) | 0.53 | 0.42 |
| Myocardial infarction | 0.94 (0.58, 1.52) | 0.80 | 0.92 (0.55, 1.55) | 0.76 | 0.82 |
| Spontaneous MI | 0.75 (0.39, 1.45) | 0.40 | 0.81 (0.37, 1.77) | 0.59 | 0.89 |
| Ischemia-driven revascularization | 0.23 (0.14, 0.39) | <0.01 | 0.12 (0.06, 0.27) | <0.01 | 0.29 |
| Stroke | 1.03 (0.35, 3.03) | 0.96 | 0.18 (0.02, 1.54) | 0.12 | 0.16 |

The multivariable Cox regression model including the following variables: GDMT as a time-dependent variable, age, gender, diabetes mellitus, hypertension, hypercholesterolemia, current smoking status, family history of coronary artery disease, previous myocardial infarction, acute coronary syndromes, and PCI treatment.

GDMT=guideline-directed medical therapy; CI=confidence interval; HR=hazard ratio; ACS= acute coronary syndrome; CCS=chronic coronary syndromes; MACCE=major adverse cardiac and cerebrovascular events; MI=myocardial infarction; PMI= periprocedural myocardial infarction.

# eTable 4. Sensitivity analysis of the effect of QFR guidance and angiography guidance on outcome events.

|  | **QFR-guided** | | **Angio-guided** | | **P for interaction** |
| --- | --- | --- | --- | --- | --- |
|  | **Adjusted HR**  **(95% CI)** | ***P* value** | **Adjusted HR**  **(95% CI)** | ***P* value** |  |
| MACCE | 0.45 (0.31,0.65) | <0.01 | 0.65 (0.48,0.87) | <0.01 | 0.57 |
| MACCE excluding PMI | 0.33 (0.21,0.52) | <0.01 | 0.56 (0.40,0.79) | <0.01 | 0.28 |
| All-cause death | 1.09 (0.39,3.03) | 0.87 | 1.30 (0.51,3.30) | 0.58 | 0.42 |
| Myocardial infarction | 0.84 (0.47,1.50) | 0.56 | 1.01 (0.65,1.55) | 0.98 | 0.82 |
| Spontaneous MI | 0.38 (0.12,1.21) | 0.10 | 0.96 (0.54,1.71) | 0.90 | 0.89 |
| Ischemia-driven revascularization | 0.22 (0.12,0.41) | <0.01 | 0.19 (0.11,0.34) | <0.01 | 0.29 |
| Stroke | 0.34 (0.06,1.77) | 0.20 | 1.23 (0.41,3.67) | 0.71 | 0.16 |

The multivariable Cox regression model including the following variables: GDMT as a time-dependent variable, age, gender, diabetes mellitus, hypertension, hypercholesterolemia, current smoking status, family history of coronary artery disease, previous myocardial infarction, acute coronary syndromes, and PCI treatment.

CI=confidence interval; GDMT=guideline-directed medical therapy; HR=hazard ratio; MACCE=major adverse cardiac and cerebrovascular events; MI=myocardial infarction; PMI= periprocedural myocardial infarction.

# eTable 5. Sensitivity analysis of the clinical outcome events over 3 years in patients achieving FCR after PCI.

|  | **Adjusted HR**  **(95% CI)** | ***P* value** |
| --- | --- | --- |
| MACCE | 0.57(0.45,0.72) | <0.01 |
| MACCE excluding PMI | 0.47(0.36,0.62) | <0.01 |
| All-cause death | 1.28(0.64,2.56) | 0.49 |
| Myocardial infarction | 0.95(0.67,1.35) | 0.79 |
| Spontaneous MI | 0.82(0.50,1.36) | 0.45 |
| Ischemia-driven revascularization | 0.21(0.14,0.32) | <0.01 |
| Stroke | 0.77(0.32,1.86) | 0.57 |

† Among the patients who did not undergo PCI, 11 MACCE events occurred in the patients in the non-GDMT group, which accounted for 1.51% of the total number of these patients, and no MACCE events occurred in the GDMT group, so the results of these patients are not shown in this table.

The multivariable Cox regression model including the following variables: GDMT as a time-dependent variable, age, sex, diabetes mellitus, hypertension, hypercholesterolemia, current smoker，family history of CAD, previous MI, stable angina, acute coronary syndromes. Abbreviations as in Supplemental Table 2

# eFigure 1. Percent of participants adhered to GDMT through 3 years.

The proportion of participants who adhered to GDMT was calculated based on the analyzable population, as illustrated in Figure 1, at each follow-up visit time point. GDMT for the treatment of coronary artery disease following the index procedure includes the following four agents: 1.) Antiplatelet drugs; 2.) Beta-blockers; 3.) ACEI or ARBs; 4.) Statins.

GMDT = guideline-directed medical therapy.


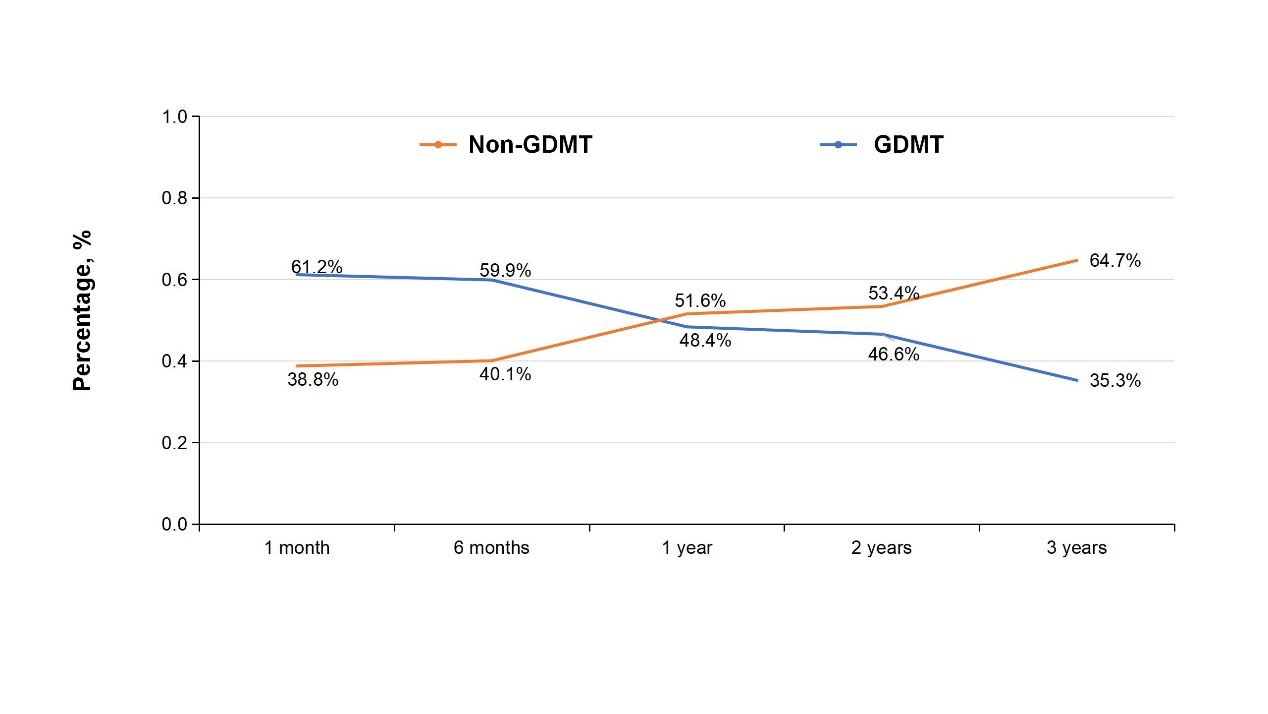


# eFigure 2. Effect of GDMT on MACCE at each follow-up timepoint.

Adjusted hazard ratios with 95% confidence intervals are shown. The multivariable Cox regression model including the following variables: GDMT as each time-point, age, gender, diabetes mellitus, hypertension, hypercholesterolemia, current smoking status, family history of coronary artery disease, previous MI, acute coronary syndromes, and PCI treatment.
GDMT was associated with significantly reduced MACCE begins at 2 years. MACCE defined as a composite of death, all myocardial infarction, ischemia-driven revascularization, or stroke.
GMDT=guideline-directed medical therapy; MACCE=major adverse cardiac and cerebrovascular events.


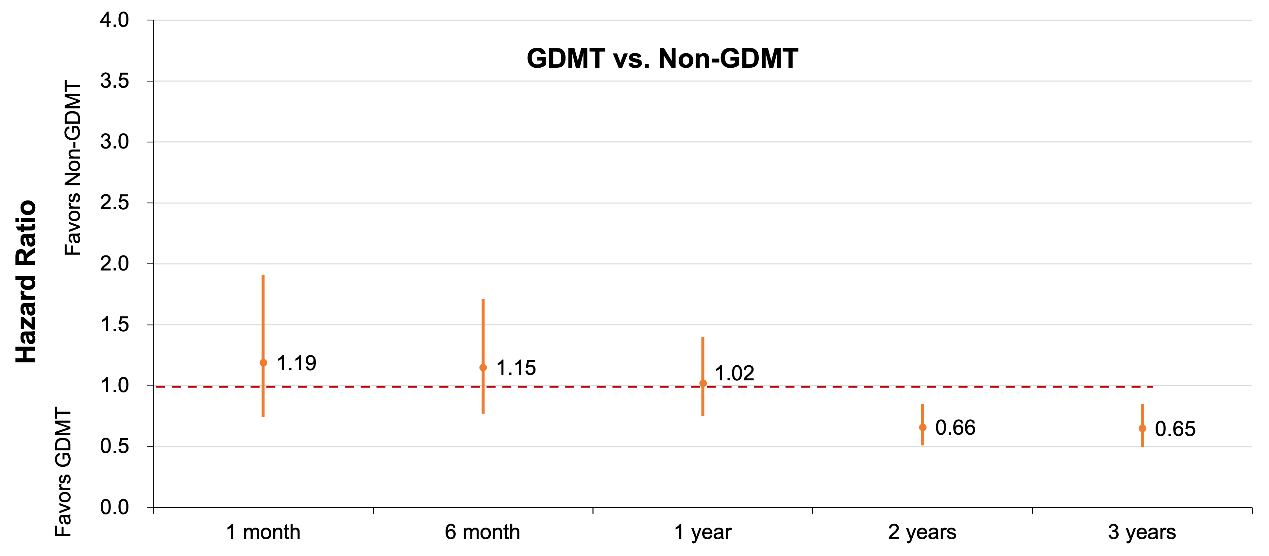


# eFigure 3. Proportion of participants who achieved a given number of GDMT goals over the follow-up period.

A total of 3,209 (99.6%), 3,206 (99.5%), 3,192 (99.1%), 3,180 (98.7%), and 3,073 (95.3%) patients, who completed clinical follow-ups and had complete medication information, were included in the analyses at 1 month, 6 months, 1 year, 2 years, and 3 years, respectively. GDMT for the treatment of coronary artery disease following the index procedure includes the following four agents: 1.) Antiplatelet drugs; 2.) Beta-blockers; 3.) ACE inhibitors (or ARBs); 4.) Statins.

The GDMT goals are illustrated as follows: 4 = adhered to all 4 prescribed agents; 3 = adhered to 3 prescribed agents; 2 = adhered to 2 prescribed agents; 1 = adhered to 1 prescribed agent; 0 = did not adhere to any prescribed agents.

GMDT = guideline-directed medical therapy.

**
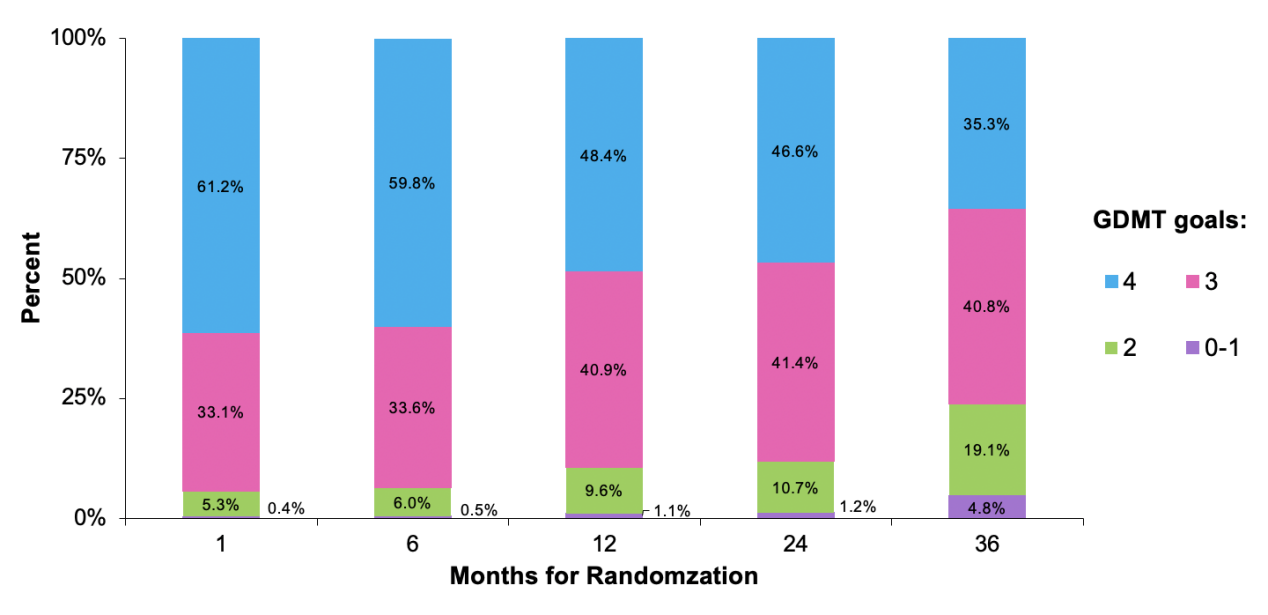
**

# eFigure 4. Percent of participants adhered to each prescribed agents through 3 years.

The proportion of participants who adhered to the following four agents: 1.) Antiplatelet drugs; 2.) Beta-blockers; 3.) ACE inhibitors (or ARBs); 4.) Statins.

**
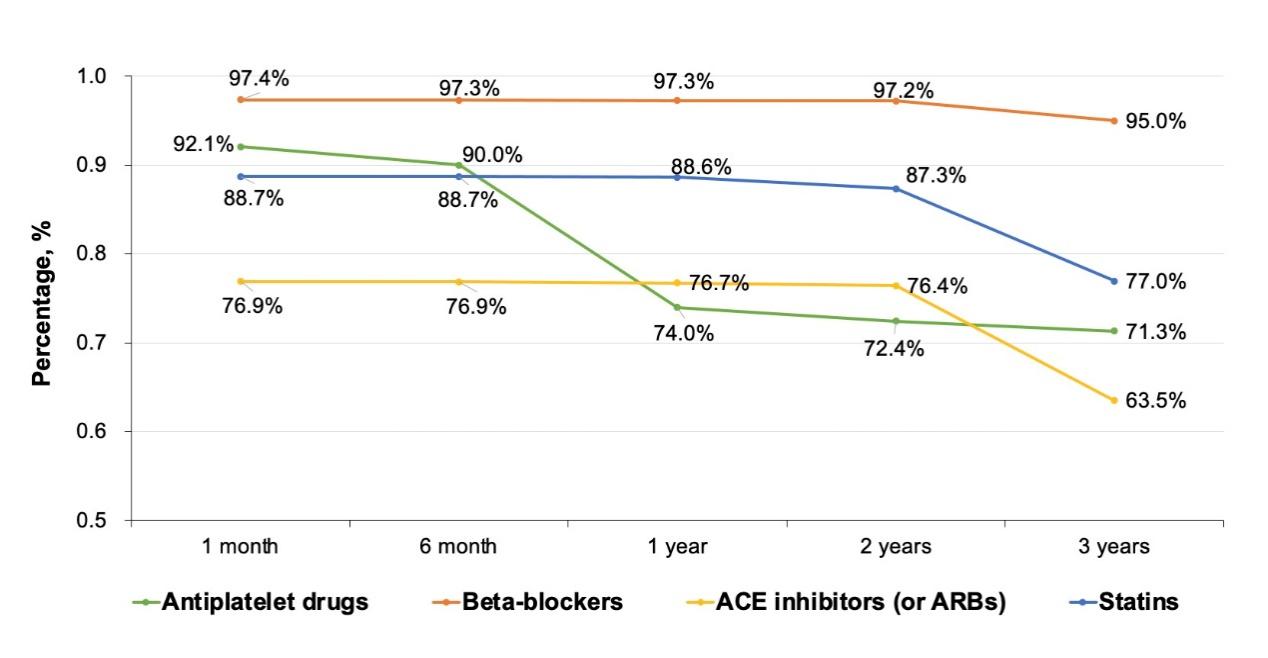
**

# eFigure 5. Sensitivity analysis for varying GDMT intensity and its impact on MACCE.

The forest plot showed the impact of different GDMT intensities on MACCE. Multivariable Cox regression adjusted hazard ratios with 95% confidence intervals are shown. The multivariable Cox regression model including the following variables: GDMT as each time-point, age, gender, diabetes mellitus, hypertension, hypercholesterolemia, current smoking status, family history of coronary artery disease, previous MI, acute coronary syndromes, and PCI treatment.

GDMT for the treatment of coronary artery disease following the index procedure includes the following four agents: 1.) Antiplatelet drugs; 2.) Beta-blockers; 3.) ACE inhibitors (or ARBs); 4.) Statins. The GDMT goals are illustrated as follows: 4 = adhered to all 4 prescribed agents; 3 = adhered to 3 prescribed agents; 2 = adhered to 2 prescribed agents; 1 = adhered to 1 prescribed agent; 0 = did not adhere to any prescribed agents.

GMDT=guideline-directed medical therapy; MACCE=major adverse cardiac and cerebrovascular events.


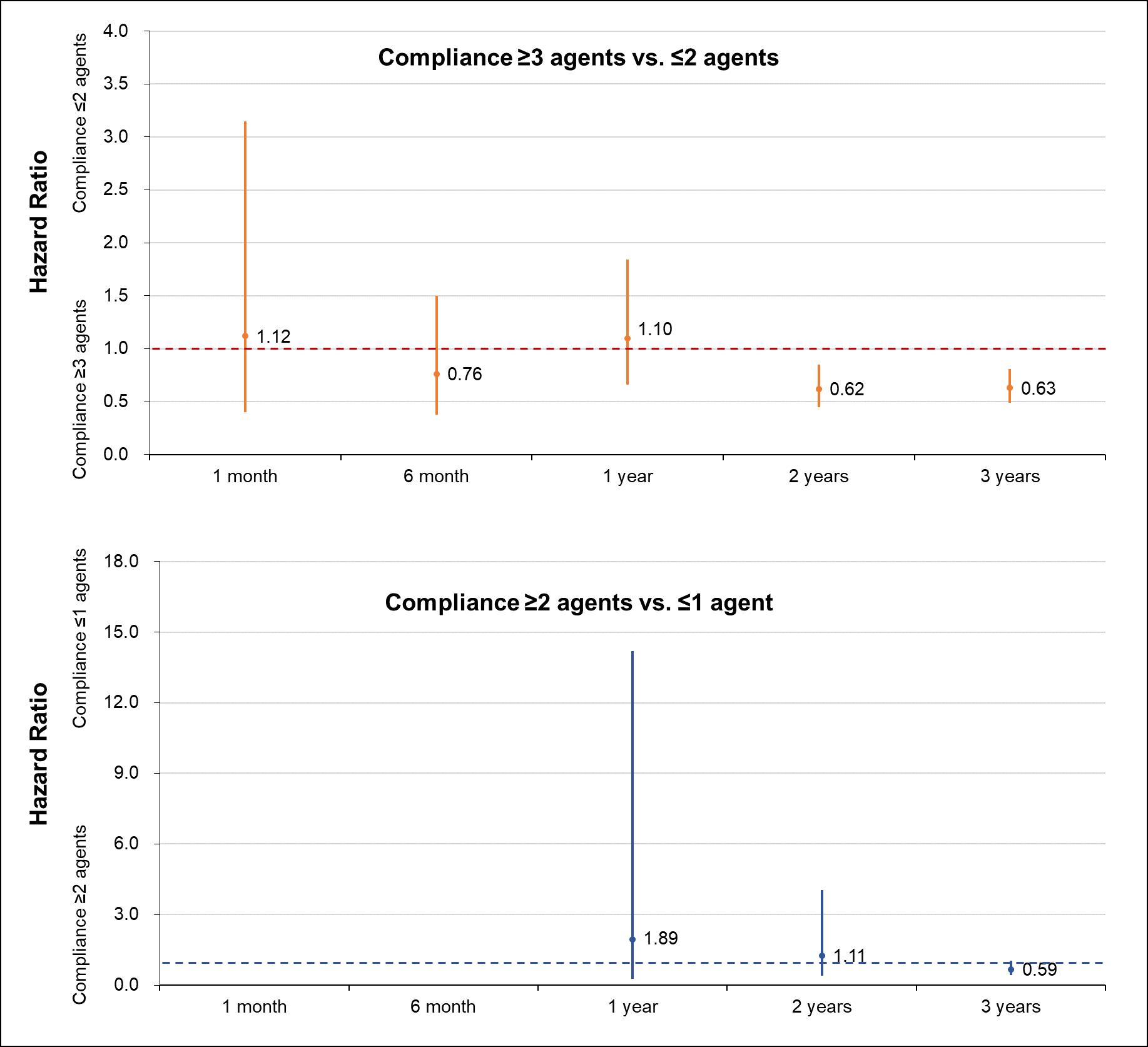


# eFigure 6. Sensitivity analysis of prescribed medications on MACCE at each follow-up timepoint.

The forest plot presents adjusted hazard ratios (HR) with 95% confidence intervals (CI) from multivariable Cox regression, illustrating the impact of individual medications on MACCE. The regression model was adjusted for prescribed medications at each follow-up timepoint, age, gender, diabetes mellitus, hypertension, hypercholesterolemia, current smoking status, family history of coronary artery disease, previous myocardial infarction (MI), acute coronary syndrome, and PCI treatment. MACCE = major adverse cardiac and cerebrovascular events.
**
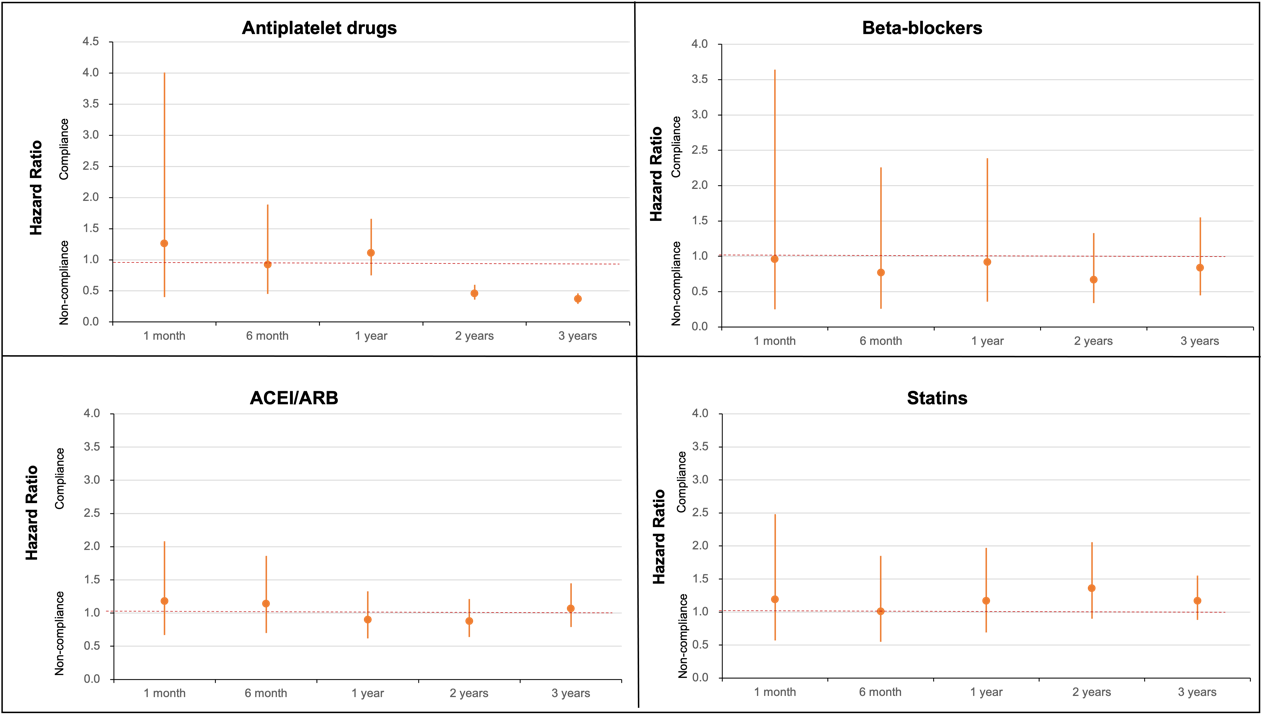
**
